# Targeting the E3 ubiquitin ligase RNF10 ameliorates metabolic dysfunction–associated steatohepatitis in mice

**DOI:** 10.1172/JCI194148

**Published:** 2026-08-06

**Authors:** Chunyuan Du, Yinliang Zhang, Hongkai Chang, Chaofan Xu, Sufang Sheng, Ke Xu, Wei Qiao, Yanjun Liu, Tongtong Zhang, Yong Gao, Peng Li, Yongsheng Chang

**Affiliations:** 1 Key Laboratory of Immune Microenvironment and Disease (Ministry of Education), Tianjin Key Laboratory of Cellular Homeostasis and Disease, Department of Physiology and Pathophysiology, School of Basic Medical Sciences, Eye Institute and School of Optometry, Tianjin Medical University Eye Hospital, Tianjin Medical University, Tianjin, China.; 2 Department of Gastroenterology, State Key Laboratory of Digestive Health, National Clinical Research Center for Digestive Diseases, Beijing Friendship Hospital, Capital Medical University, Beijing, China.; 3 Department of Endocrinology and Metabolism, Clinical Research Center, Shandong Provincial Key Medical and Health Discipline of Endocrinology, Affiliated Hospital of Shandong Second Medical University, Weifang, China.; 4 Obesity and Metabolism Medicine–Engineering Integration Laboratory and; 5 Center of Gastrointestinal and Minimally Invasive Surgery, Department of General Surgery, The Affiliated Hospital of Southwest Jiaotong University, The Third People’s Hospital of Chengdu, Chengdu, China.; 6 State Key Laboratory of Traditional Chinese Medicine Syndrome, Science and Technology Innovation Center, Guangzhou University of Chinese Medicine, Guangzhou, China.; 7 Tianfu Jincheng Laboratory, Chengdu, China.

**Keywords:** Hepatology, Metabolism, Fatty acid oxidation, Gene therapy, Hepatitis

## Abstract

Metabolic dysfunction–associated steatotic liver disease (MASLD) has emerged as a global health concern. Nevertheless, its underlying pathological mechanisms remain poorly understood. Here, we showed that E3 ubiquitin ligase RING finger protein 10 (RNF10) protein levels were positively correlated with MASLD in both mice and humans. Hepatocyte-specific *Rnf10* deletion attenuated liver steatosis, inflammation, and fibrosis. Conversely, adeno-associated virus–mediated hepatocyte-specific *Rnf10* overexpression exacerbated MASLD-related phenotypes. Mechanistically, RNF10 interacted with carnitine palmitoyltransferase 1A and facilitated its degradation through K48-linked ubiquitination, thereby inhibiting fatty acid oxidation, promoting hepatic lipid accumulation, and ultimately exacerbating liver inflammation and fibrosis. Moreover, we utilized triantennary *N*-acetylgalactosamine to deliver siRNA specifically targeting *Rnf10* to hepatocytes. This approach effectively ameliorated diet-induced liver steatosis, inflammation, and fibrosis in mice. Therefore, interfering with the expression or function of RNF10 may be a promising therapeutic strategy for MASLD.

## Abstract 2

RNF10 drivers MASLD by promoting CPT1A degradation via K48-linked ubiquitination. GalNAc-si*Rnf10* targeting hepatic RNF10 effectively mitigates MASLD.

## Graphical abstract

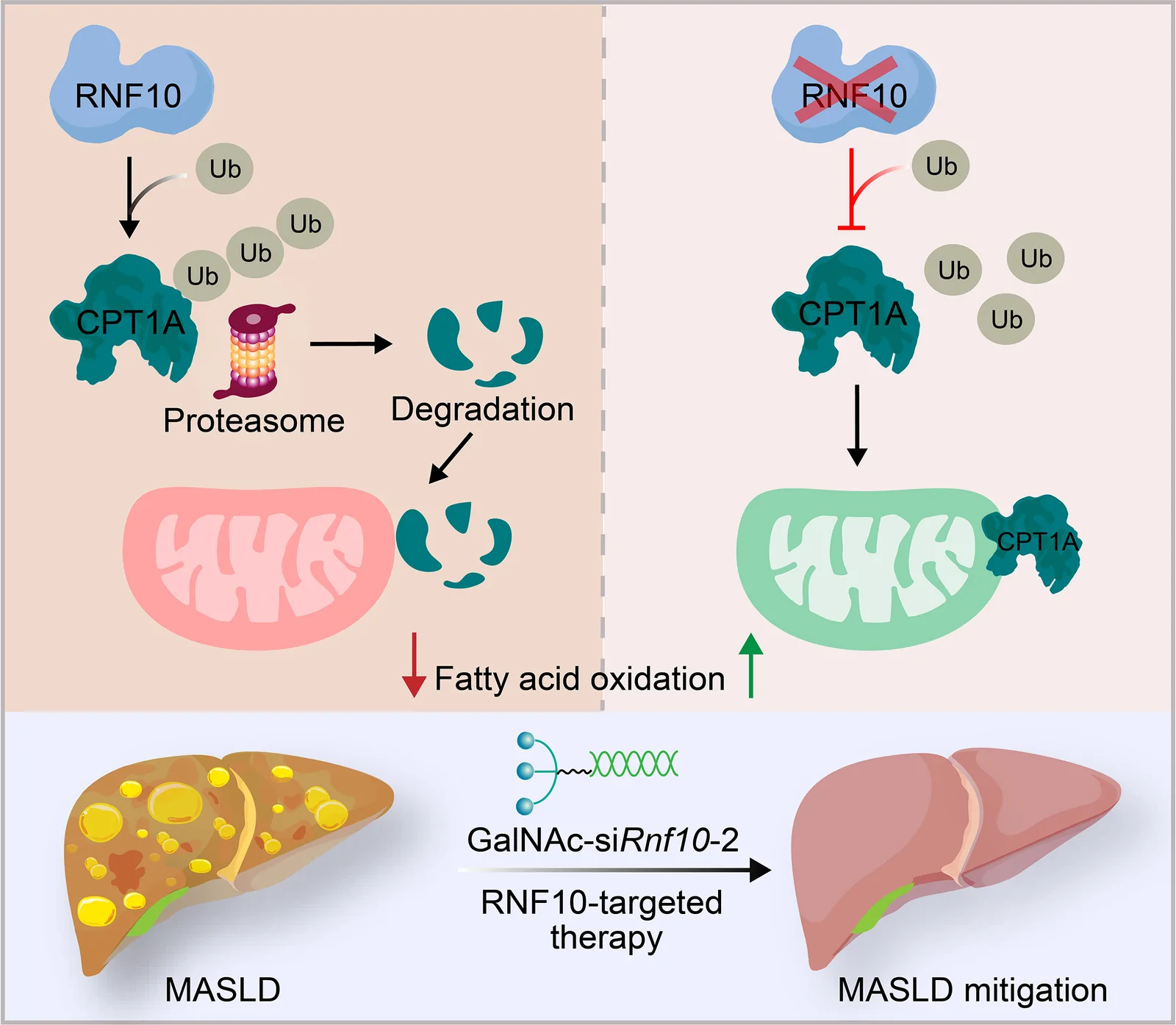

## Introduction

Metabolic dysfunction–associated steatotic liver disease (MASLD), previously termed nonalcoholic fatty liver disease (NAFLD), is the most prevalent chronic liver disease worldwide, affecting more than 30% of the world’s population (1–3). MASLD is closely associated with multiple metabolic risk factors, including obesity, dyslipidemia, and type 2 diabetes, and is an independent risk factor for cardiovascular diseases (4, 5). MASLD encompasses a disease spectrum ranging from simple steatosis to metabolic dysfunction–associated steatohepatitis (MASH) and eventually to cirrhosis, which is a major contributor to end-stage liver disease (6, 7). Currently, there are few drugs approved for the treatment of MASLD (8, 9). Therefore, investigating the mechanisms underlying MASLD and identifying potential therapeutic targets are essential.

Increasing fatty acid β-oxidation (FAO) in the liver counteracts MASLD (10, 11). One of the key enzymes controlling FAO is carnitine palmitoyltransferase (CPT), which consists of 3 proteins in the liver: carnitine palmitoyltransferase 1A (CPT1A), CPT2, and carnitine-acylcarnitine translocase (CACT) (12, 13). CPT1A converts acyl-CoAs into acylcarnitines, which are transported across the mitochondrial membrane by CACT and reconverted into acyl-CoAs inside the mitochondria by CPT2 for β-oxidation (14). CPT1A is considered the rate-limiting enzyme for FAO, and its activation is a strategy for the treatment of MASLD (15–17). Resmetirom is the first FDA-approved drug for the treatment of MASH, and one of its mechanisms is to upregulate the transcription of *CPT1A*, thereby accelerating FAO and fatty acid consumption and reducing liver fat content (18–20).

E3 ubiquitin ligases control ubiquitination, which is a posttranslational modification that regulates cellular protein homeostasis. Recently, E3 ubiquitin ligase RING finger proteins (RNFs) were reported to play key roles in MASLD. For example, Lee et al. reported that RNF20 attenuates hepatocyte steatosis in *db/db* mice by directly interacting with SREBP1c, a key metabolic regulator of hepatic lipogenesis, promoting its ubiquitination-mediated degradation (21). In addition, RNF13 has been shown to ameliorate MASH pathology through the degradation of stimulator of interferon genes protein (STING) (22). A previous human genetic study identified RNF10 as a susceptibility gene associated with obesity and type 2 diabetes (23). However, whether and how RNF10 affects metabolic diseases remain unclear.

In the present study, we observed that RNF10 protein levels were significantly increased in livers of humans and mice with MASLD. Hepatic *Rnf10* deficiency alleviated steatosis, inflammation, and fibrosis in mice with MASLD, whereas adeno-associated virus–mediated (AAV-mediated) hepatocyte-specific overexpression of *Rnf10* exacerbated these phenotypes. Mechanistically, we found that RNF10 promotes the ubiquitination and degradation of CPT1A, which, in turn, suppresses hepatic FAO, thereby exacerbating MASLD phenotypes. These findings indicate that the RNF10/CPT1A axis governs hepatic lipid homeostasis and identify RNF10 as a potential therapeutic target for MASLD.

## Results

### Hepatic RNF10 protein levels are increased in MASLD.

**Figure 1:**
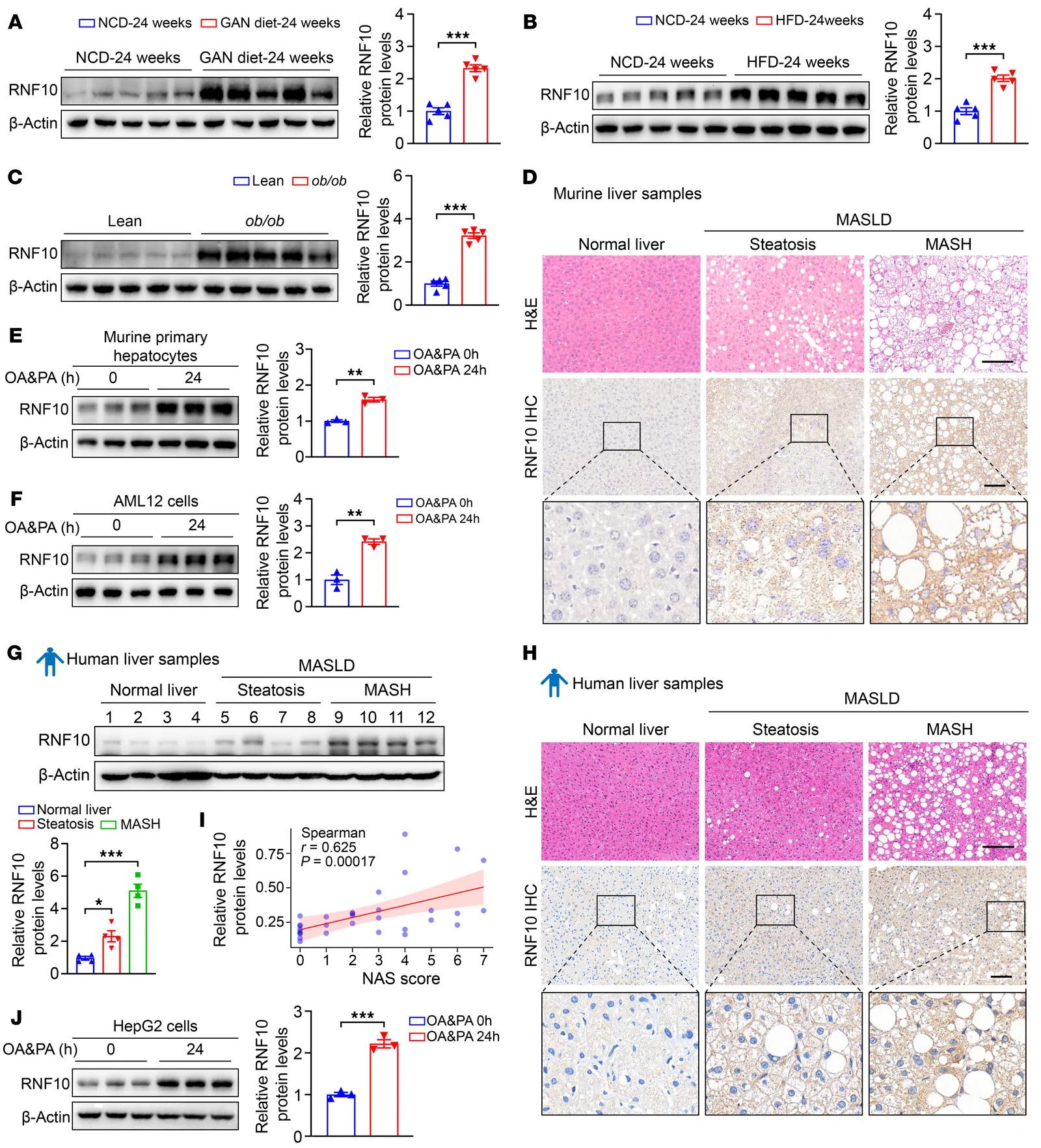
Hepatic RNF10 protein levels are increased in MASLD. (**A** and **B**) Western blot analysis and quantification of RNF10 expression in the livers of mice fed a NCD, GAN diet, or HFD for 24 weeks (*n* = 5 mice/group). (**C**) Relative RNF10 protein levels in the livers of lean and *ob/ob* mice (*n* = 5 mice/group). (**D**) Representative images of H&E (top) and immunohistochemical staining (middle and bottom) in murine liver sections without steatosis (12-week NCD-fed WT mice), with simple steatosis (12-week HFD-fed WT mice), or with MASH (24-week GAN diet–fed WT mice). Scale bars: 50 μm. (**E** and **F**) Western blot analysis and quantification of RNF10 protein levels in murine primary hepatocytes (**E**) or AML12 cells (**F**) treated with OA&PA for 24 hours (*n* = 3 independent experiments). (**G**) Representative Western blot analysis of RNF10 levels in liver tissue from individuals with normal liver, with steatosis, or with MASH (*n* = 4 individuals/group). (**H**) Representative immunohistochemical staining images of RNF10 levels in liver tissue from individuals with normal liver, with steatosis, or with MASH. Scale bars: 50 μm. (**I**) Correlation analysis for RNF10 protein levels and NAS in human liver samples (*n* = 31 individuals). (**J**) Western blot analysis and quantification of RNF10 protein levels in HepG2 cells treated with OA&PA for 24 hours (*n* = 3 independent experiments). Data are presented as mean ± SEM. Unpaired 2-tailed Student’s *t* test was performed in **A**–**C**, **E**, **F**, and **J**; 1-way ANOVA was performed in **G**, and Spearman’s correlation analysis was performed in **I**. *P < 0.05, **P < 0.01, and ***P < 0.001.

To explore the physiological function of RNFs in the liver, we first analyzed their expression profiles using transcriptomic datasets from mouse and human livers. We found that *RNF10* and *RNF181* are highly expressed in liver of both mice and humans, ranking among the top 5 most highly expressed RNFs (Supplemental Figure 1, A and B; supplemental material available online with this article; https://doi.org/10.1172/JCI194148DS1). Of note, compared with controls, hepatic RNF181 protein levels were unaltered in high-fat, high-fructose, high-cholesterol (GAN) diet–induced MASLD mouse models (Supplemental Figure 1C). However, RNF10 was upregulated in different mouse models of MASLD, including GAN diet–induced, high-fat diet–induced (HFD-induced), and *ob/ob* mouse models (Figure 1, A–C). Moreover, the RNF10 protein level gradually increased with disease progression (Figure 1D). Consistently, RNF10 protein levels were also increased in murine hepatocytes treated with 1.0 mM OA&PA (oleic acid and palmitic acid; 2:1) (Figure 1, E and F), a well-characterized cell model of MASLD (24). Notably, *Rnf10* mRNA levels remained unchanged in both the mouse models and the cell model of MASLD (Supplemental Figure 1, D and E). Thus, RNF10 protein was subjected to posttranslational modifications in MASLD.

Next, we explored the molecular mechanisms underlying the upregulation of RNF10 protein levels during MASLD progression. We first performed cycloheximide (CHX; an inhibitor of protein synthesis) chase assays and found that OA&PA treatment decreased RNF10 protein degradation in hepatocytes (Supplemental Figure 1F). Eukaryotic cells have 2 major degradation systems: the lysosomal pathway and the proteasomal pathway (22). Notably, CHX-mediated decrease in RNF10 protein levels was largely recovered by cotreatment with lysosome inhibitor (chloroquine) in murine primary hepatocytes, while the 26S proteasome inhibitor (MG132) had much less effect on RNF10 protein recovery compared with chloroquine (Supplemental Figure 1G). Thus, these data suggest that excessive fatty acids possibly reduce lysosome-mediated RNF10 degradation.

We subsequently analyzed the human relevance of RNF10 in MASLD. Consistent with our findings in mouse models, RNF10 protein levels were significantly higher in the livers of patients with simple steatosis and MASH than in those of normal controls (Figure 1, G and H). Notably, RNF10 protein levels exhibited a strong positive correlation with NAFLD activity score (NAS), while *RNF10* mRNA levels remained stable across all groups (Figure 1I and Supplemental Figure 1H). Consistently, OA&PA treatment also increased RNF10 protein levels in HepG2 cells without altering its mRNA levels (Figure 1J and Supplemental Figure 1I). Taken together, hepatic RNF10 protein levels are increased in MASLD, suggesting that RNF10 plays a role in the pathogenesis of MASLD.

### Hepatocyte-specific Rnf10 deficiency ameliorates MASLD in mice.

To investigate the functional role of RNF10 in MASLD, we generated hepatocyte-specific *Rnf10*-knockout (*Rnf10*-HKO) mice by crossing Alb-Cre mice with *Rnf10*^fl/fl^ mice (flanking exon 2 of *Rnf10* with loxP sites). Littermates lacking the Cre gene (*Rnf10*-Flox) were used as controls (Supplemental Figure 2, A–D). Western blot analysis confirmed that RNF10 was successfully deleted in the livers of *Rnf10*-HKO mice but not in other tissues (Supplemental Figure 2E).

**Figure 2:**
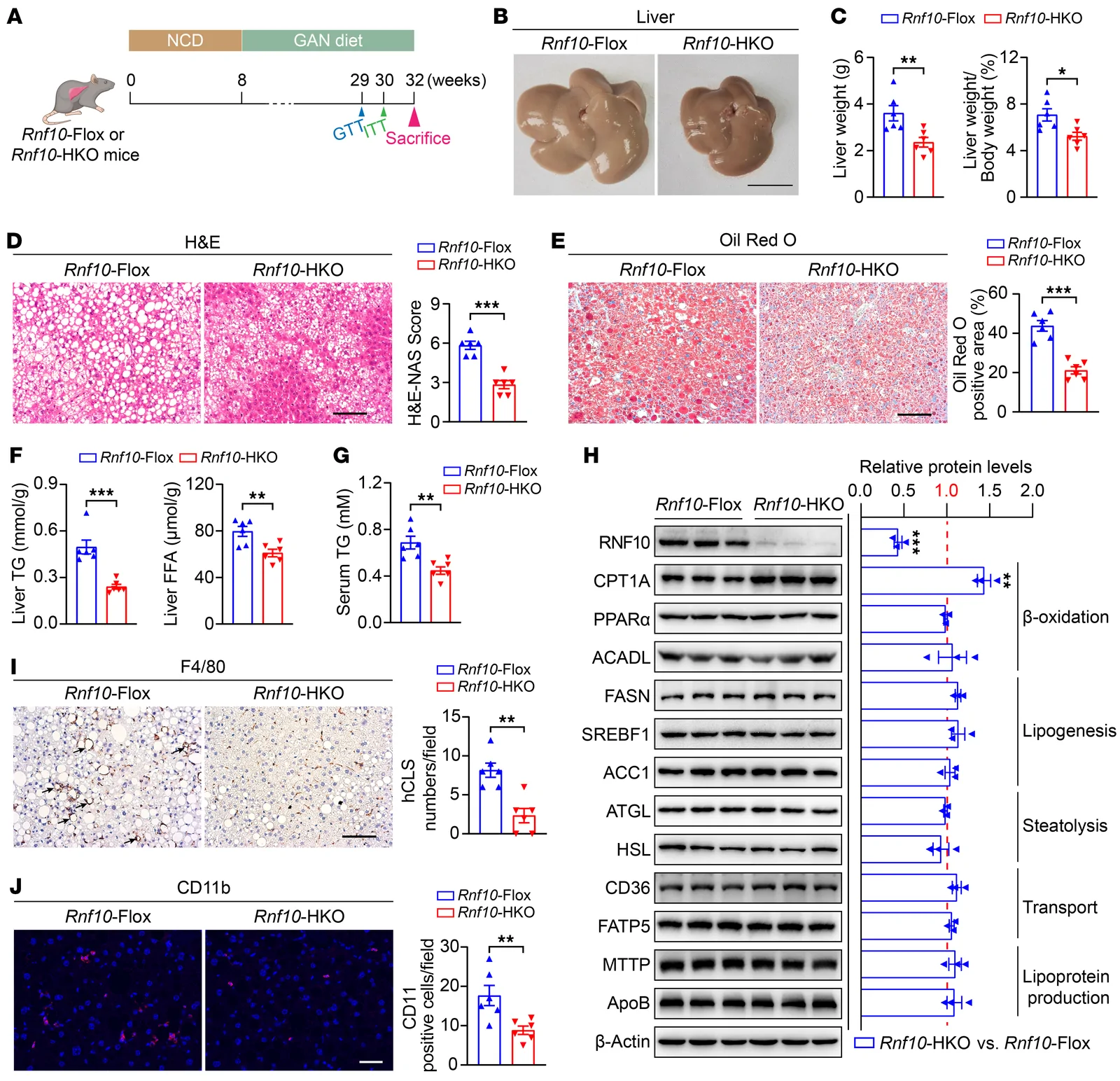
Hepatocyte-specific *Rnf10* deficiency ameliorates GAN diet–induced MASH in mice. (**A**) Schematic of the feeding protocol for mice with hepatocyte-specific deletion of *Rnf10* (*Rnf10*-HKO) for the data shown in **B**–**J**. *Rnf10*-Flox was used as the control. Mice were fed a GAN diet for 24 weeks. (**B**) Gross liver morphology. Scale bar: 1 cm. (**C**) Liver weight and the LW/BW ratio (*n* = 6 mice/group). (**D** and **E**) Representative H&E (**D**) and Oil Red O (**E**) staining images; quantitative results are shown on the right (*n* = 6 mice/group). Scale bar: 50 μm. (**F** and **G**) Levels of hepatic TG and FFA (**F**) and serum TG (**G**) (*n* = 6 mice/group). (**H**) Relative protein levels of lipid metabolism markers in the liver (β-oxidation, lipogenesis, steatolysis, fatty acid transport, and lipoprotein production) (*n* = 3 mice/group). (**I**) Representative immunohistochemical staining for F4/80^+^ immune cells; quantitative results are shown on the right (*n* = 6 mice/group). hCLS, hepatic crown-like structure. Scale bar: 50 μm. (**J**) Representative immunofluorescent staining for CD11b^+^ immune cells; quantitative results are shown on the right (*n* = 6 mice/group). Scale bar: 50 μm. Data are presented as mean ± SEM. Unpaired 2-tailed Student’s *t* test was performed comparing *Rnf10*-HKO with *Rnf10*-Flox mice. *P < 0.05, **P < 0.01, and ***P < 0.001.

Next, *Rnf10*-HKO mice were fed a normal chow diet (NCD) (Supplemental Figure 2F) or a GAN diet (Figure 2A) for 24 weeks. Under NCD conditions, *Rnf10*-HKO mice presented a phenotype similar to that of their littermate controls but slightly improved systemic glucose metabolism (Supplemental Figure 2, G–O). Under GAN diet conditions, compared with *Rnf10*-Flox mice, *Rnf10*-HKO mice exhibited visibly smaller livers and presented reductions in liver weight, the liver weight/body weight ratio (LW/BW; %), body weight, and fat mass (Figure 2, B and C, and Supplemental Figure 3A). Histological analysis (H&E and Oil Red O staining) revealed reduced hepatic steatosis and NAS in *Rnf10*-HKO mice (Figure 2, D and E). Consistently, biochemical assessments confirmed lower hepatic triglyceride (TG), free fatty acid (FFA), and serum TG contents in the GAN diet–fed *Rnf10*-HKO mice than in the control mice (Figure 2, F and G). Moreover, we found that *Rnf10* deletion significantly increased CPT1A (a rate-limiting enzyme in FAO) protein levels, whereas no obvious changes were observed in other proteins associated with hepatic FAO (PPARα and ACADL), lipogenesis (FASN, SREBF1, and ACC1), steatolysis (ATGL and HSL), fatty acid transport (CD36 and FATP5), and lipoprotein secretion (MTTP and ApoB) (Figure 2H). However, *Rnf10* deletion did not affect the mRNA levels of these lipid metabolism–related genes, including *Cpt1a* (Supplemental Figure 3B). To functionally assess the impact of *Rnf10* deficiency on hepatic FAO, we measured serum β-hydroxybutyrate (β-OHB) levels (25). Notably, β-OHB levels were elevated in *Rnf10*-HKO mice compared with control mice, suggesting that *Rnf10* deletion enhances hepatic FAO (Supplemental Figure 3C). Consistently, in both mouse and human cell models of MASLD, *Rnf10* deficiency alleviated steatosis and increased CPT1A protein and FAO (Supplemental Figure 3, D–M).

**Figure 3:**
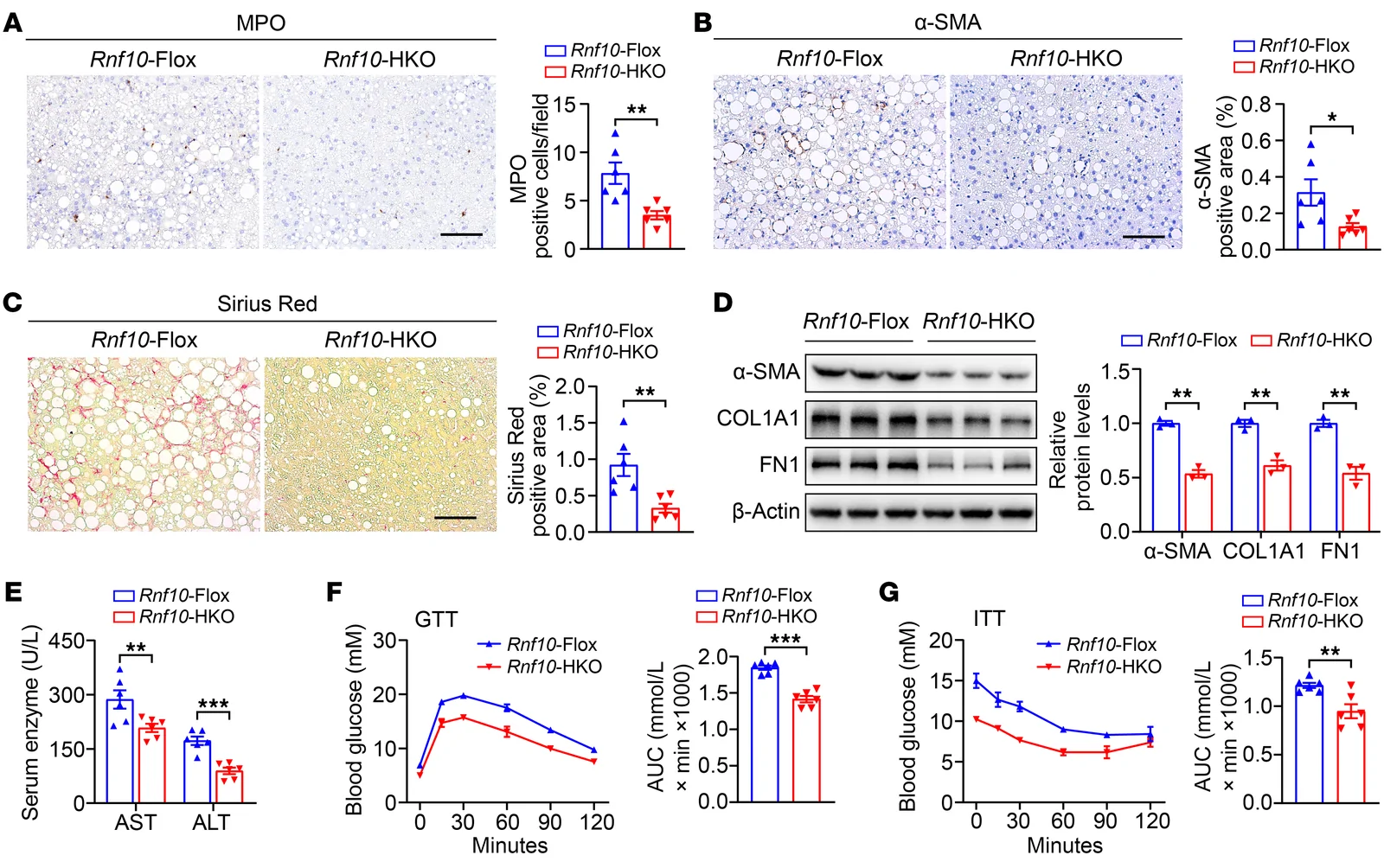
Hepatocyte-specific *Rnf10* deficiency ameliorates GAN diet–induced MASH in mice. (**A**) Representative immunohistochemical staining for MPO^+^ immune cells; quantitative results are shown on the right (*n* = 6 mice/group). Scale bar: 50 μm. (**B** and **C**) Representative images of α-SMA immunohistochemical staining (**B**) and Sirius Red staining (**C**); quantitative results are shown on the right (*n* = 6 mice/group). Scale bars: 50 μm. (**D**) Relative levels of the indicated proteins related to liver fibrosis (*n* = 3 mice/group). (**E**) Serum AST and ALT levels (*n* = 6 mice/group). (**F** and **G**) Glucose tolerance test (GTT) (**F**) and insulin tolerance test (ITT) (**G**) with the corresponding AUC (*n* = 6 mice/group). Data are presented as mean ± SEM. Unpaired 2-tailed Student’s *t* test was performed comparing *Rnf10*-HKO with *Rnf10*-Flox mice. *P < 0.05, **P < 0.01, and ***P < 0.001.

Hepatic steatosis can lead to lipotoxicity, resulting in liver inflammation and fibrosis (26). By examining F4/80^+^ (a marker of macrophages) cells in the livers of GAN diet–fed *Rnf10*-Flox and *Rnf10*-HKO mice, we found that *Rnf10* deficiency reduced the density of hepatic crown-like structures, which are macrophages that surround and phagocytose necrotic hepatocytes, a condition commonly observed in MASH livers (Figure 2I). We also examined the infiltration of CD11b^+^ immune cells in the liver and found that *Rnf10* deficiency reduced the number of immune cells (Figure 2J). MPO staining demonstrated that *Rnf10* deficiency also reduced hepatic neutrophil infiltration (Figure 3A). In addition, qPCR analysis revealed that *Rnf10* deficiency reduced the expression of inflammatory genes (*Tnf*, *Ccl2*, *Cxcl2*, *Il6*, and *Il1b*) (Supplemental Figure 3N). Furthermore, *Rnf10* deficiency limited the progression of fibrosis, as shown by a decrease in α-smooth muscle actin^+^ (α-SMA^+^; a marker of activated hepatic stellate cells [HSCs]) area, and average collagen deposition (Figure 3, B and C). Similarly, the expression of fibrotic genes — such as tissue inhibitor of metalloproteinase 1; actin alpha 2, smooth muscle (which encodes α-SMA); collagen type I alpha 1 chain; fibronectin 1; and connective tissue growth factor — was decreased in *Rnf10*-HKO mice (Figure 3D and Supplemental Figure 3O). Moreover, *Rnf10*-HKO mice presented less liver injury, as evidenced by decreased serum aspartate aminotransferase (AST) and alanine aminotransferase (ALT) levels (Figure 3E). Thus, *Rnf10* deficiency ameliorates hepatic inflammation and fibrosis. Hepatic steatosis is often associated with insulin resistance (27). As expected, *Rnf10*-HKO mice presented improved glucose intolerance and insulin resistance (Figure 3, F and G, and Supplemental Figure 3P). Thus, *Rnf10* deficiency ameliorates hepatic steatosis.

To confirm these findings, we fed *Rnf10*-HKO mice an HFD for 24 weeks (Supplemental Figure 4A). Compared with control mice, HFD-fed *Rnf10*-HKO mice presented reduced body weight, fat mass, liver size, liver weight, LW/BW ratio, hepatic lipid content, and NAS (Supplemental Figure 4, B–G). In addition, serum TG levels were reduced in the *Rnf10*-HKO mice (Supplemental Figure 4H). Moreover, hepatic *Rnf10* deletion increased CPT1A protein levels without altering its mRNA levels (Supplemental Figure 4, I and J). Consistently, serum β-OHB levels were also elevated in *Rnf10*-HKO mice (Supplemental Figure 4K). *Rnf10*-HKO mice also presented less inflammation and liver injury (Supplemental Figure 4, L–N) as well as improved glucose intolerance and insulin resistance (Supplemental Figure 4, O and P).

To explore whether the *Rnf10* deficiency alleviates MASLD independently of changes in body weight, we challenged *Rnf10*-HKO mice with a choline-deficient, L–amino acid–defined, high-fat diet (CDAHFD) for 7 weeks. CDAHFD typically induces hepatic steatosis, inflammation, and fibrosis without changing body weight of mice (28). As a result, *Rnf10* deficiency mitigated CDAHFD-induced hepatic steatosis, inflammation, and fibrosis in the absence of changes in body weight. Furthermore, *Rnf10* deficiency significantly elevated CPT1A protein levels, while *Cpt1a* mRNA expression remained unaffected (Supplemental Figure 5). These results confirm that *Rnf10* deficiency ameliorates MASLD.

### RNF10 mediates the K48-linked polyubiquitination of CPT1A.

**Figure 4:**
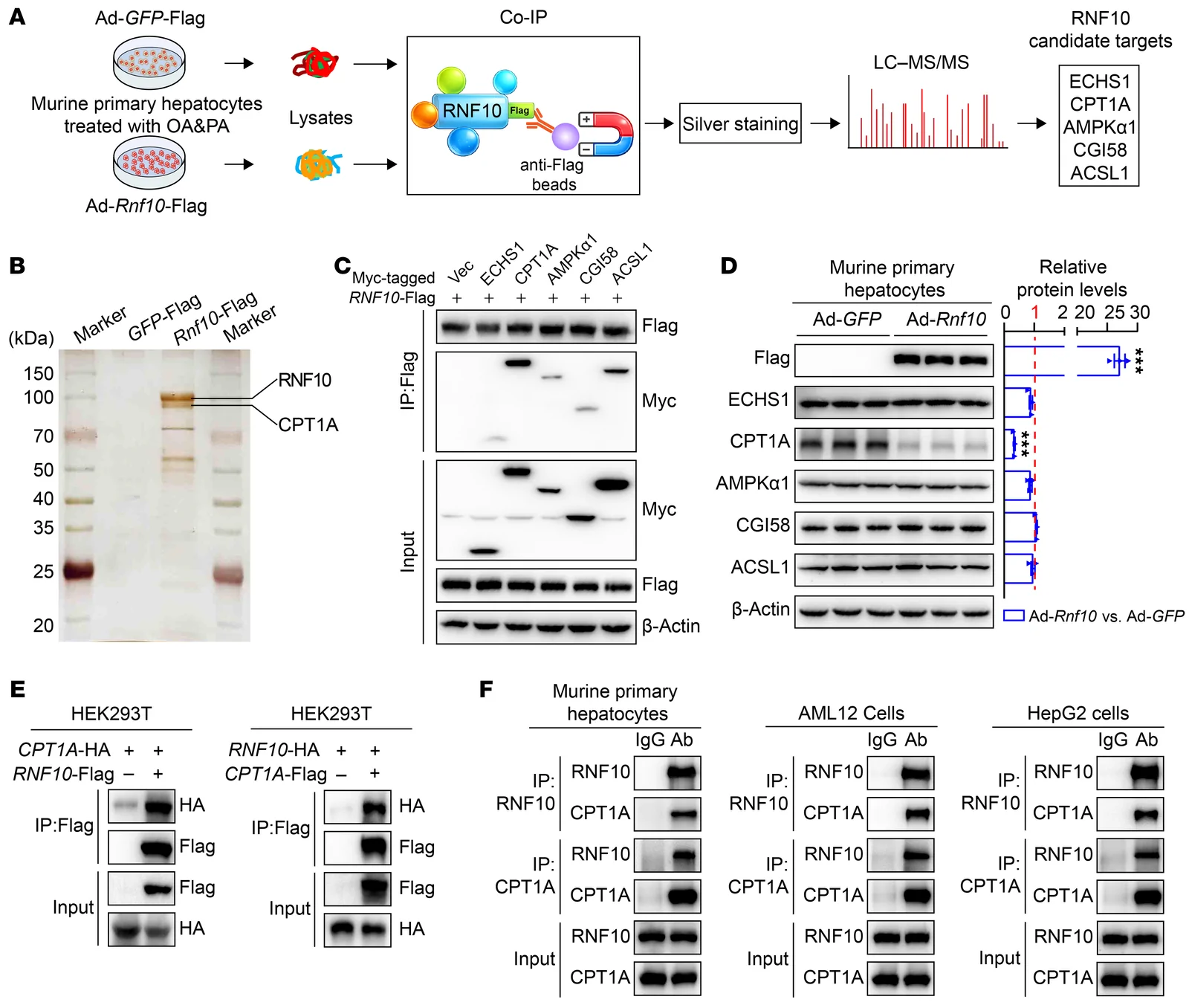
Identification of CPT1A as a target of RNF10 in hepatocytes. (**A**) Schematic representation of the co-IP and liquid chromatography–tandem mass spectrometry (LC-MS/MS) workflow. (**B**) Silver-stained gel showing the indicated protein binding to RNF10. (**C**) Representative co-IP analysis evaluating the interaction between RNF10 and candidate targets (ECHS1, CPT1A, AMPK α1, CGI58, and ACSL1). (**D**) Western blot showing the effect of RNF10 on candidate targets in murine primary hepatocytes (*n* = 3 independent experiments). (**E**) Representative co-IP analysis between endogenous RNF10 and CPT1A in murine primary hepatocytes, AML12 cells, and HepG2 cells. IgG served as a negative control. (**F**) Representative co-IP analysis between exogenous RNF10 and CPT1A in HEK293T cells. Data are presented as mean ± SEM. Unpaired 2-tailed Student’s *t* test was performed in **D**. ***P < 0.001.

**Figure 5:**
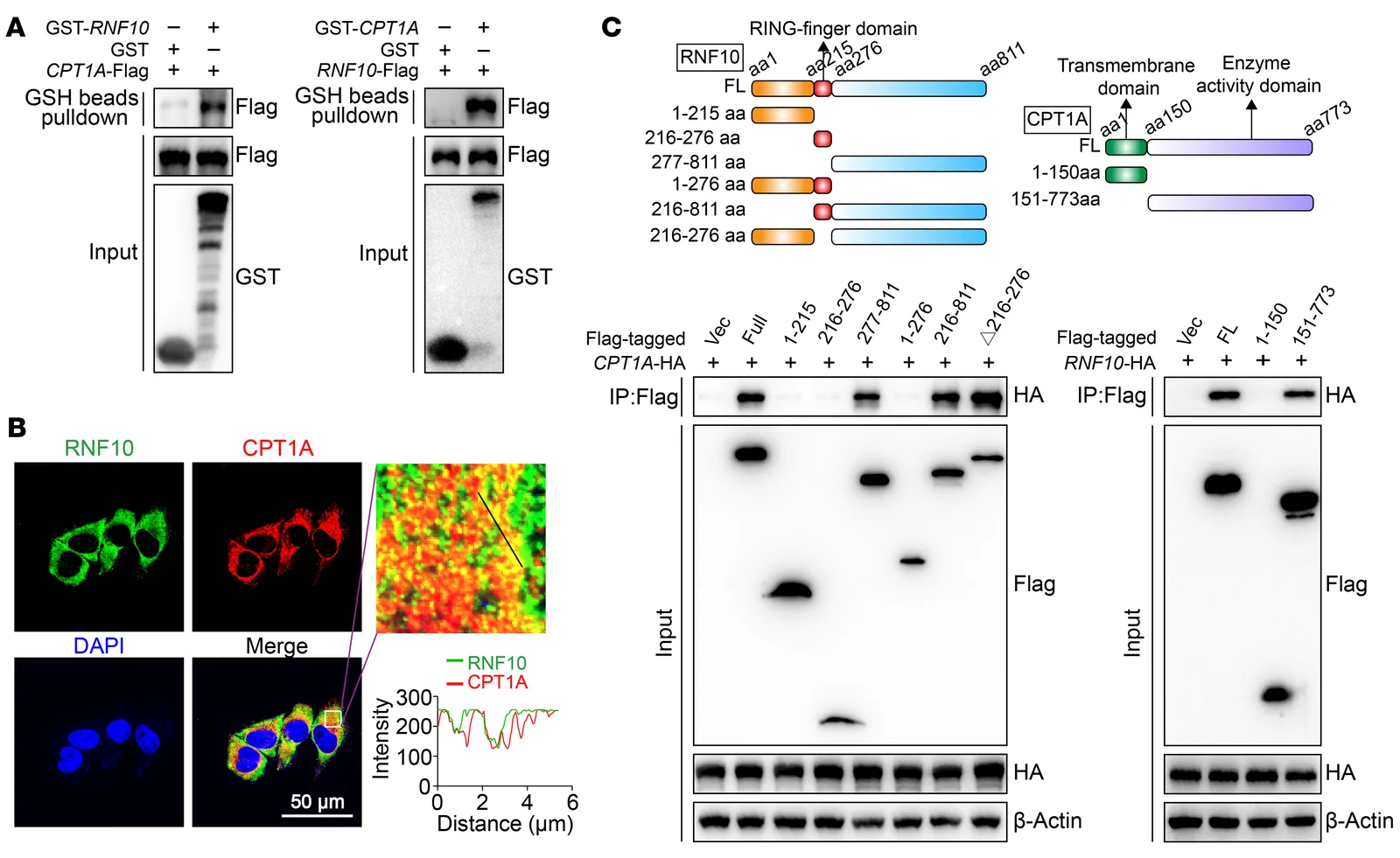
Identification of CPT1A as a target of RNF10 in hepatocytes. (**A**) Representative GST pull-down assays showed direct binding between RNF10 and CPT1A. Purified GST was used as the control. (**B**) Colocalization of RNF10 (green) and CPT1A (red) in HepG2 cells. DAPI (blue) counterstain for nuclei is shown in the bottom panels. Relative fluorescence intensity profiles of the green and red channels measured along the indicated line in the inset. Scale bar: 50 μm. (**C**) Schematics of the RNF10 and CPT1A full-length and fragment constructs; the interaction domains of RNF10 and CPT1A were explored based on co-IP assays.

We next investigated the molecular basis of RNF10-mediated posttranslational regulation of CPT1A. First, we overexpressed exogenous RNF10 in hepatocytes and performed liquid chromatography–tandem mass spectrometry. We found that RNF10 interacted with CPT1A, along with several other lipid metabolism–related proteins, such as ECHS1, AMPKα1, CGI58, and ACSL1 (Figure 4, A and B). These interactions were further validated by co-IP assays (Figure 4C). Among these candidates, only CPT1A protein levels were reduced upon *Rnf10* overexpression in murine primary hepatocytes (Figure 4D). These findings implied that CPT1A is the potential key target of RNF10 in lipid metabolism. Notably, CPT1A interacted with RNF10 in both mouse and human cells (Figure 4, E and F). GST pull-down experiments confirmed that RNF10 directly interacted with CPT1A in vitro (Figure 5A). Consistent with these findings, immunofluorescence staining revealed the colocalization of RNF10 and CPT1A in HepG2 cells (Figure 5B). Next, we generated truncated mutants of RNF10 and CPT1A to investigate the functional domains involved in the RNF10–CPT1A interaction. Our results indicate that the 276– to 811–amino acid domain of RNF10 is responsible for its interaction with the 151– to 773–amino acid domain of CPT1A (Figure 5C). Together, these data suggest that RNF10 directly interacts with CPT1A.

**Figure 6:**
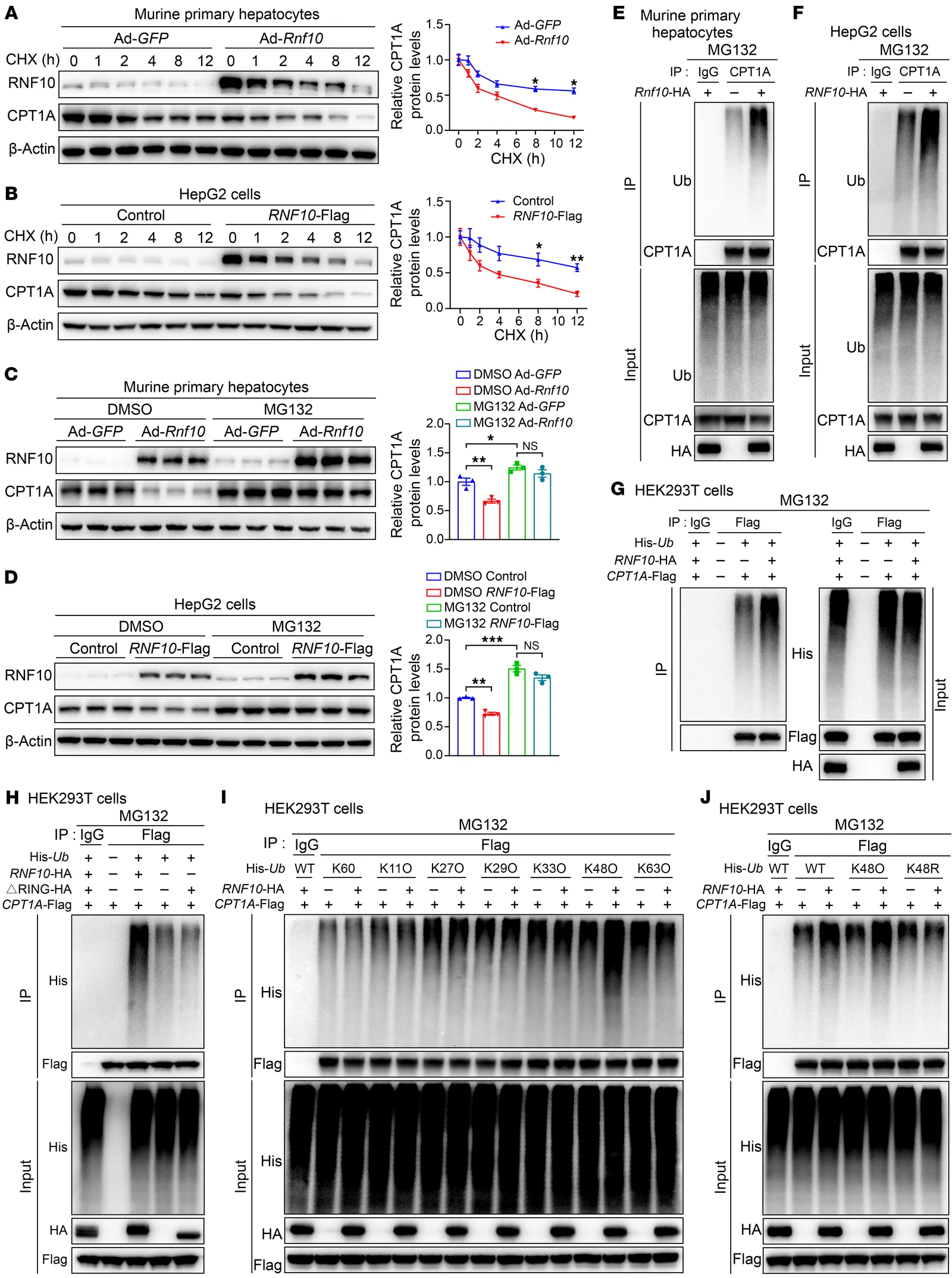
RNF10 facilitates CPT1A protein degradation through K48-linked ubiquitination. Relative CPT1A protein levels in murine primary hepatocytes (**A**) and HepG2 cells (**B**) with *RNF10* overexpression, followed by CHX (100 μg/mL) treatment. Quantitative results are shown on the right (*n* = 3 independent experiments). (**C** and **D**) Relative CPT1A protein levels in murine primary hepatocytes (**C**) and HepG2 cells (**D**) with *RNF10* overexpression in the absence or presence of MG132 (20 μM). The right panels show quantitative analyses of the Western blot results (*n* = 3 independent experiments). (**E** and **F**) Ubiquitination of endogenous CPT1A induced by *RNF10* overexpression in murine primary hepatocytes (**E**) and HepG2 cells (**F**) in the presence of MG132 (20 μM). IgG was used as a negative control. (**G** and **H**) Ubiquitination of exogenous CPT1A induced by *RNF10* (**G**) or *RNF10^ΔRING^* (**H**) overexpression in HEK293T cells that were treated with MG132 (20 μM). IgG was used as a negative control. (**I** and **J**) Screening of potential lysine ubiquitination types of CPT1A by RNF10 with the indicated types of ubiquitin in the presence of MG132 (20 μM). K6O, K11O, and so on, are ubiquitin-mutated proteins (e.g., K6O means that only the number 6 lysine residue [K] is retained, whereas the rest of the lysine residues are mutated to arginine; K48R means that only the number 48 lysine residue is mutated to arginine [R], whereas the rest of the lysine residues are retained). IgG was used as a negative control. Data are presented as mean ± SEM. Unpaired 2-tailed Student’s *t* test was performed in **A** and **B**, and 1-way ANOVA was performed in **C** and **D**. *P < 0.05, **P < 0.01, and ***P < 0.001.

As an E3 ligase, RNF10 regulates ubiquitination-mediated protein degradation. Therefore, we examined the protein levels of CPT1A at different time points following treatment with CHX. We found that *Rnf10* overexpression increased the degradation rate of CPT1A in murine primary hepatocytes (Figure 6A) and HepG2 cells (Figure 6B). Moreover, the degradation of CPT1A protein promoted by *Rnf10* overexpression was reversed by MG132 in murine primary hepatocytes (Figure 6C) and HepG2 cells (Figure 6D). The initial step in ubiquitin-proteasome system–mediated protein degradation is the binding of ubiquitin to the target protein. IP experiments confirmed that the binding of ubiquitin to endogenous CPT1A was induced by RNF10 under the treatment of MG132 in murine primary hepatocytes (Figure 6E) and HepG2 cells (Figure 6F). Moreover, the ubiquitination of exogenous CPT1A markedly increased when RNF10 was overexpressed (Figure 6G), and this modification was dependent on the RNF10 RING finger domain (Figure 6H). Given that different types of ubiquitination lead to distinct molecular processes, the screening of mutated forms of ubiquitin for potential types of lysine ubiquitination revealed that the degradation of CPT1A was mediated by RNF10 through K48-linked ubiquitination (Figure 6, I and J). Previous studies have shown that mutation of the 2 cysteine residues in the RING domain of RNF10 (C225/C228) abolishes its E3 ligase activity (29). Consistent with this study, our results showed that the RNF10 C225S/C228S mutant (*Rnf10^mut^*) failed to promote CPT1A ubiquitination and degradation and consequently had negligible effects on hepatic lipid metabolism (Supplemental Figure 6). Taken together, RNF10 interacts with CPT1A and promotes its proteasomal degradation via K48-linked ubiquitination, which relies on the E3 ligase activity of RNF10.

### RNF10 requires CPT1A to modulate FAO and MASH progression.

To investigate whether CPT1A mediates the effects of RNF10 on FAO, we silenced *CPT1A* via lentivirus-mediated knockdown in *RNF10*-deficient hepatocytes (Supplemental Figure 7, A and E). In murine primary hepatocytes and HepG2 cells, *CPT1A* knockdown abolished the *RNF10* deletion–mediated attenuation of OA&PA-induced lipid accumulation (Supplemental Figure 7, B, C, F, and G). *RNF10* depletion increased β-OHB levels, while *CPT1A* knockdown reversed this effect, leading to a reduction in β-OHB levels (Supplemental Figure 7, D and H). Next, we quantified FAO activity using a Seahorse XF24 analyzer. *RNF10*-knockdown hepatocytes treated with an OA&PA mixture exhibited a marked increase in the oxygen consumption rate, whereas *CPT1A* knockdown reversed this effect, leading to a reduction in oxygen consumption rate (Supplemental Figure 7, I and J). Moreover, *RNF10*-knockdown hepatocytes were more sensitive to OA&PA and etomoxir (a CPT1A inhibitor), supporting the role of RNF10 in regulating FAO via CPT1A (Supplemental Figure 7, K and L). Collectively, these data indicated that CPT1A is required for the enhanced FAO observed upon *RNF10* depletion.

**Figure 7:**
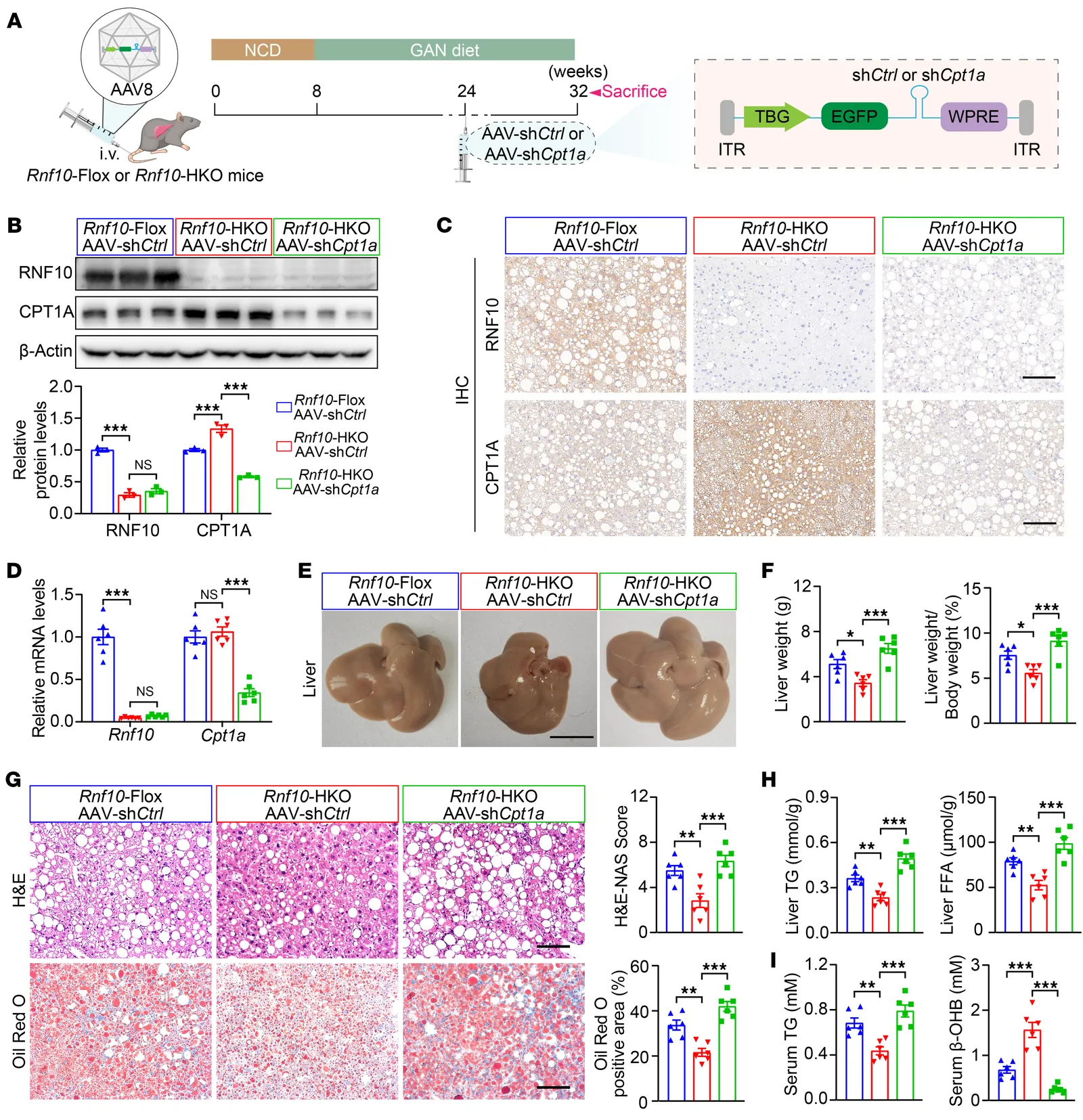
Knockdown of *Cpt1a* blunts the effects of hepatocyte-specific deficiency of *Rnf10* in MASH. (**A**) Schematic of the experiment analyzing the role of CPT1A in mediating the effect of *Rnf10* deletion in GAN diet–induced MASH and schematic of AAV-sh*Cpt1a* structure. After 16 weeks of GAN diet feeding, *Rnf10*-Flox mice injected with AAV-sh*Ctrl* and *Rnf10*-HKO mice injected with either AAV-sh*Ctrl* or AAV-sh*Cpt1a* continued a GAN diet for an additional 8 weeks. Data are shown in **B**–**I**. (**B**) Relative protein levels of RNF10 and CPT1A (*n* = 3 mice/group). (**C**) Representative immunohistochemical staining of RNF10 and CPT1A in liver sections from the indicated groups. Scale bars: 50 μm. (**D**) Relative mRNA levels of *Rnf10* and *Cpt1a* (*n* = 6 mice/group). (**E**) Gross liver morphology. Scale bar: 1 cm. (**F**) Liver weight and the LW/BW ratio (*n* = 6 mice/group). (**G**) Representative H&E (top) and Oil Red O (bottom) staining images; quantitative results are shown on the right (*n* = 6 mice/group). Scale bars: 50 μm. (**H** and **I**) Liver TG and FFA contents (**H**) and serum TG and β-OHB levels (**I**) (*n* = 6 mice/group). Data are presented as mean ± SEM. 1-way ANOVA was performed in **F**–**I**, and 2-way ANOVA was performed in **B** and **D**. *P < 0.05, **P < 0.01, and ***P < 0.001.

**Figure 8:**
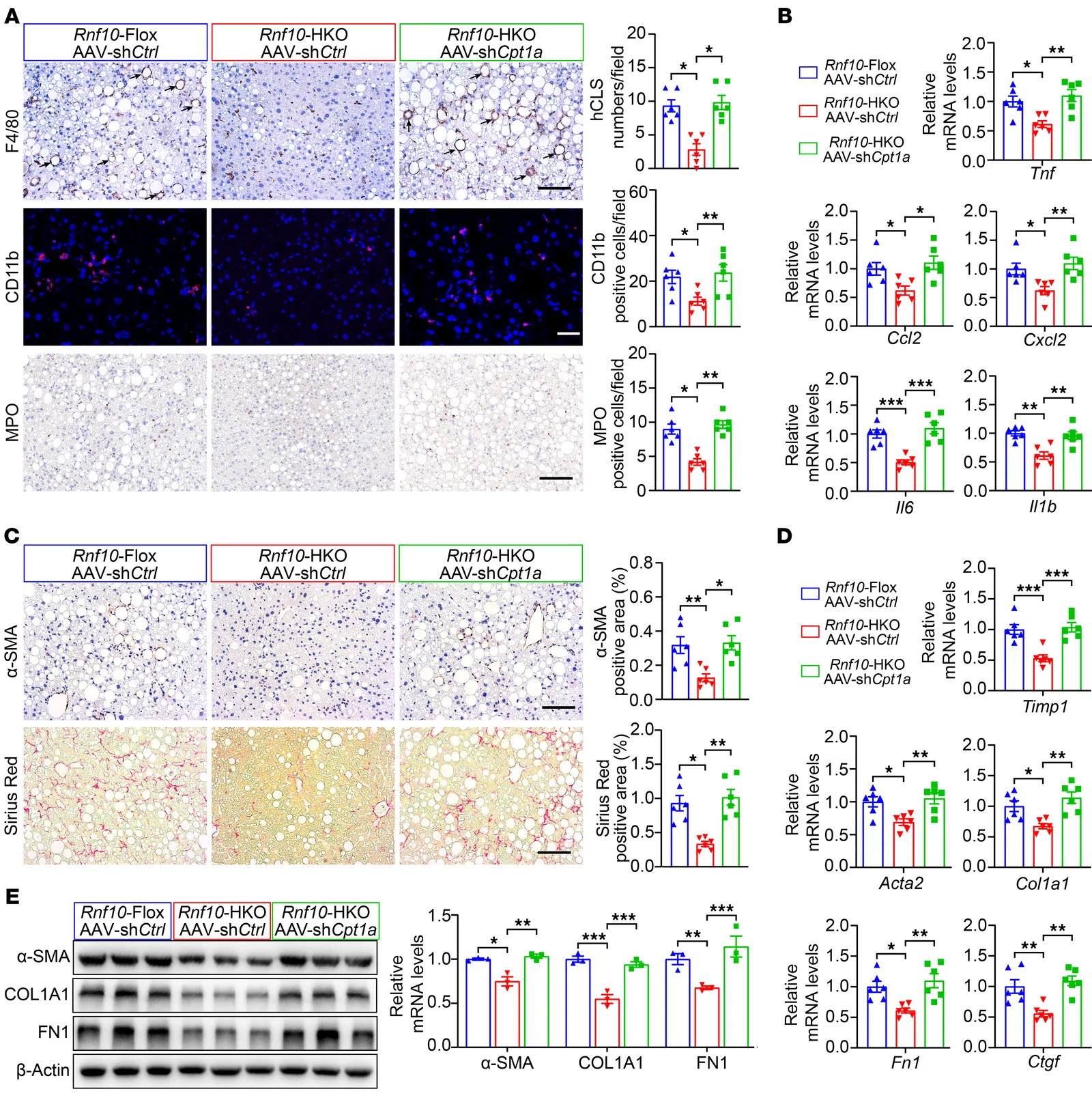
Knockdown of *Cpt1a* blunts the effects of hepatocyte-specific deficiency of *Rnf10* in MASH. (**A**) Representative F4/80 and MPO immunohistochemistry staining and CD11b immunofluorescent staining images; quantitative results are shown on the right (*n* = 6 mice/group). Scale bars: 50 μm. (**B**) Relative mRNA levels of proinflammatory genes (*n* = 6 mice/group). (**C**) Representative α-SMA and Sirius Red staining images; quantitative results are shown on the right (*n* = 6 mice/group). Scale bars: 50 μm. (**D** and **E**) Relative mRNA (**D**) (*n* = 6 mice/group) and protein (**E**) (*n* = 3 mice/group) levels of profibrotic genes. Data are presented as mean ± SEM. 1-way ANOVA was performed in **A**–**D**, and 2-way ANOVA was performed in **E**. *P < 0.05, **P < 0.01, and ***P < 0.001.

To investigate whether CPT1A mediates the effects of RNF10 on MASH progression, we performed a *Cpt1a*-knockdown experiment by administering AAV-sh*Cpt1a* via the tail vein into *Rnf10*-HKO mice (Figure 7A). AAV-sh*Cpt1a*–infected *Rnf10*-HKO mice presented a robust decrease in CPT1A expression in liver (Figure 7, B–D, and Supplemental Figure 7M). Moreover, hepatic *Cpt1a* knockdown prevented the amelioration of MASH features, including hepatic steatosis, inflammation, and fibrosis, caused by hepatocyte-specific *Rnf10* deficiency (Figure 7, E–I, and Figure 8, A–E). Notably, *Cpt1a* knockdown abolished the *Rnf10* deletion–mediated increase in β-OHB levels (Figure 7I), aligning with the in vitro findings. Furthermore, when *Cpt1a* was knocked down in the liver of *Rnf10*-HKO mice, the reduction in body weight and fat mass mediated by *Rnf10* deficiency was also reversed (Supplemental Figure 7N). Based on these in vitro and in vivo data, we concluded that the ability of RNF10 to promote MASH progression is largely dependent on impaired CPT1A protein stability, thereby disrupting FAO.

### Hepatocyte-specific Rnf10 overexpression exacerbates MASLD by downregulating CPT1A in mice.

As a complementary approach to liver-specific *Rnf10* knockout mouse models, we generated an AAV type 8 expressing RNF10 under the TBG promoter (AAV-*Rnf10*), which was specifically activated in hepatocytes (Supplemental Figure 8A). AAV-*Rnf10* was injected via the tail vein, and AAV8 expressing GFP (AAV-*GFP*) was used as the control. AAV-*Rnf10* infection increased RNF10 expression only in the liver, not in other tissues (Supplemental Figure 8, B and C).

**Figure 9:**
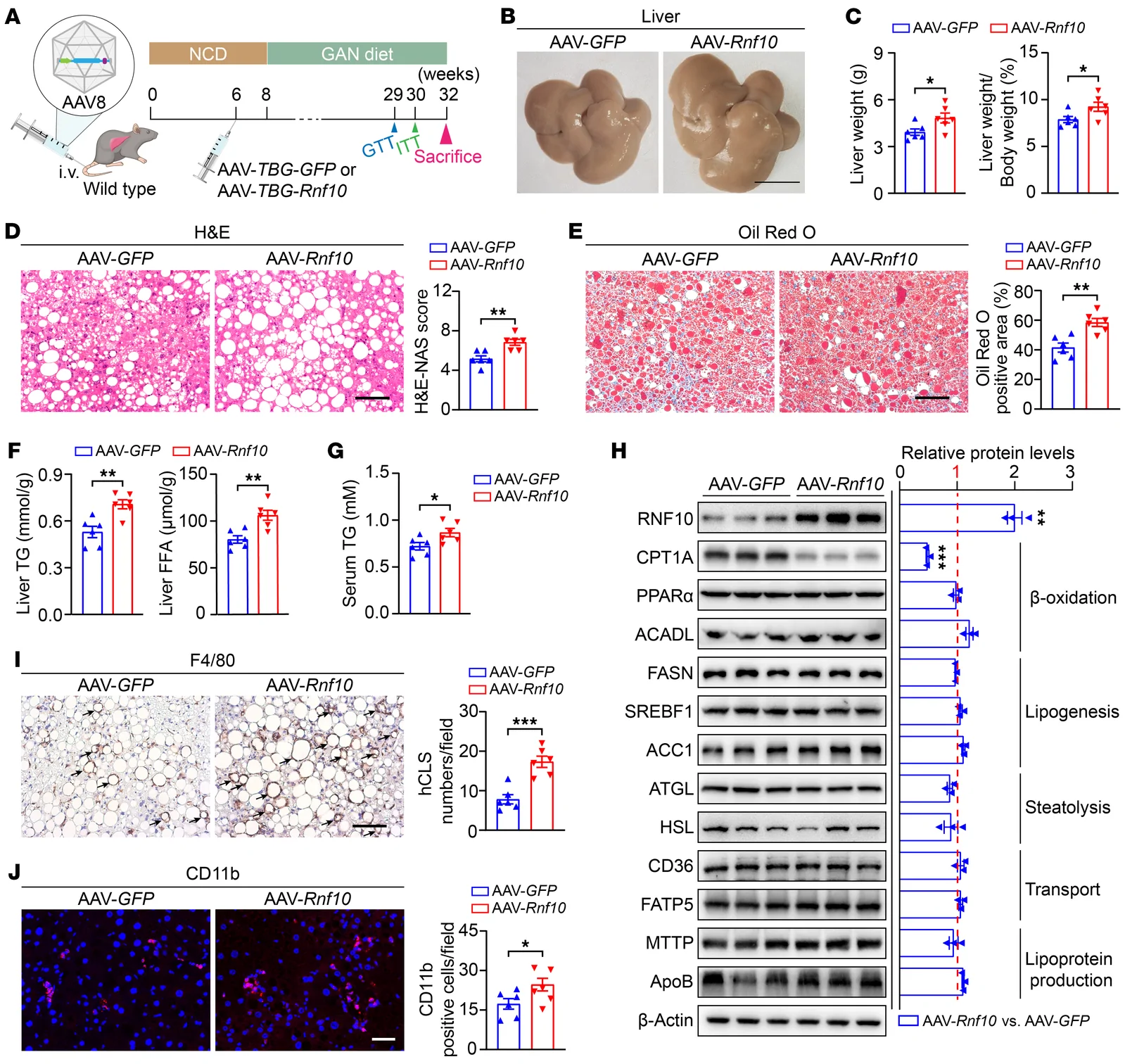
Hepatocyte-specific *Rnf10* overexpression exacerbates GAN diet–induced MASH in mice. (**A**) Schematic of GAN diet–fed AAV-*Rnf10* mice for the data shown in **B**–**J**. AAV-*GFP* was used as the control. 2 weeks following the AAV-*Rnf10* or AAV-*GFP* virus injection, mice were fed a GAN diet for 24 weeks. (**B**) Gross liver morphology. Scale bar: 1 cm. (**C**) Liver weight and the LW/BW ratio (*n* = 6 mice/group). (**D** and **E**) Representative H&E (**D**) and Oil Red O (**E**) staining images and quantified results (*n* = 6 mice/group). Scale bar: 50 μm. (**F** and **G**) Liver TG and FFA contents (**F**) and serum TG levels (**G**) (*n* = 6 mice/group). (**H**) Relative protein levels of lipid metabolism markers in the liver (β-oxidation, lipogenesis, steatolysis, fatty acid transport, and lipoprotein production) (*n* = 3 mice/group). (**I**) Representative immunohistochemical staining for F4/80^+^ immune cells; quantitative results are shown on the right (*n* = 6 mice/group). Scale bar: 50 μm. (**J**) Representative immunofluorescent staining for CD11b^+^ immune cells; quantitative results are shown on the right (*n* = 6 mice/group). Scale bar: 50 μm. Data are presented as mean ± SEM. Unpaired 2-tailed Student’s *t* tests were performed to compare the AAV-*Rnf10* and AAV-*GFP* groups. *P < 0.05, **P < 0.01, and ***P < 0.001.

**Figure 10:**
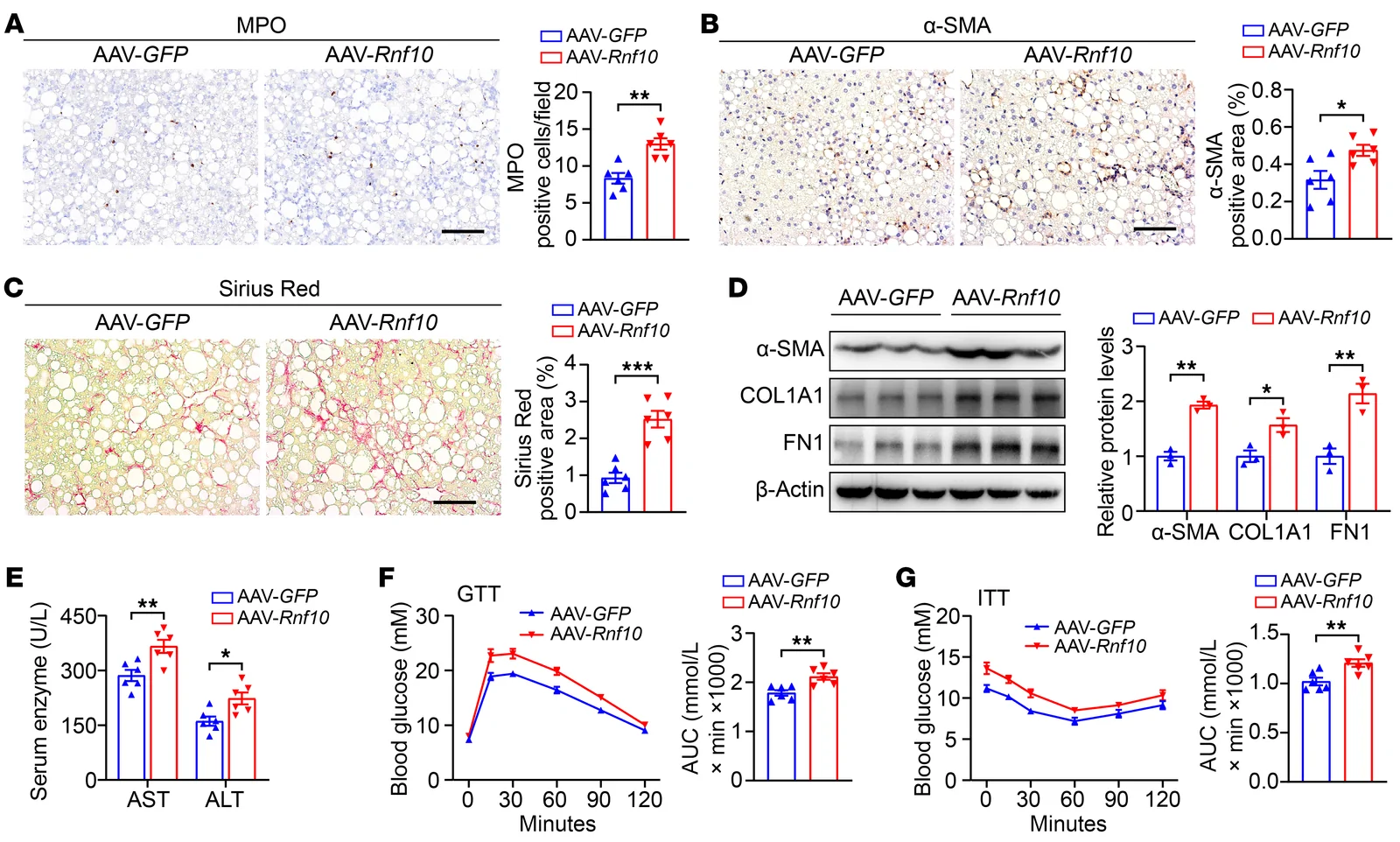
Hepatocyte-specific *Rnf10* overexpression exacerbates GAN diet–induced MASH in mice. (**A**) Representative immunohistochemical staining for MPO^+^ immune cells; quantitative results are shown on the right (*n* = 6 mice/group). Scale bar: 50 μm. (**B** and **C**) Representative images of α-SMA immunohistochemical (**B**) and Sirius Red staining (**C**); quantitative results are shown on the right (*n* = 6 mice/group). Scale bars: 50 μm. (**D**) Relative protein levels of profibrotic genes (*n* = 3 mice/group). (**E**) Serum AST and ALT levels (*n* = 6 mice/group). (**F**) GTT and the corresponding AUC (*n* = 6 mice/group). (**G**) ITT and the corresponding AUC (*n* = 6 mice/group). Data are presented as mean ± SEM. Unpaired 2-tailed Student’s *t* tests were performed to compare the AAV-*Rnf10* and AAV-*GFP* groups. *P < 0.05, **P < 0.01, and ***P < 0.001.

Under NCD conditions, *Rnf10* overexpression did not markedly affect the gross hepatic appearance but slightly increased hepatic lipid accumulation and impaired systemic glucose metabolism (Supplemental Figure 8, D–L). Subsequently, *Rnf10*-overexpressing and control mice were fed a GAN diet to induce MASLD (Figure 9A). In these models, *Rnf10* overexpression consistently increased liver size, liver weight, the LW/BW ratio, body weight, and fat mass (Figure 9, B and C, and Supplemental Figure 9A). Additionally, hepatic lipid accumulation was markedly elevated, accompanied by higher NAS, increased hepatic TG and FFA content, and elevated serum TG levels (Figure 9, D–G). Moreover, *Rnf10* overexpression reduced hepatic CPT1A protein level and decreased serum β-OHB levels (Figure 9H and Supplemental Figure 9, B and C). Consistently, *Rnf10* overexpression exacerbated steatosis and decreased CPT1A protein and FAO in both mouse and human cell models of MASLD (Supplemental Figure 9, D–M). In addition, MASLD model mice with hepatic *Rnf10* overexpression exhibited exacerbated hepatic inflammation and/or hepatic fibrosis, glucose intolerance, and insulin sensitivity (Figure 9, I and J, Figure 10, and Supplemental Figure 9, N–P). Similar results were obtained in HFD-fed MASLD mice (Supplemental Figure 10). However, AAV-mediated overexpression of *Cpt1a* in GAN diet–induced MASLD models improved liver dysfunction caused by *Rnf10* overexpression (Supplemental Figure 11). Taken together, these results demonstrate that RNF10 exacerbates MASLD by downregulating CPT1A.

### Hepatocyte-directed GalNAc-siRnf10-2 ameliorates GAN diet–induced MASH in mice.

The targeted delivery of oligonucleotides to hepatocytes using *N*-acetylgalactosamine (GalNAc) conjugates that bind to the asialoglycoprotein receptor has become a breakthrough approach in the therapeutic oligonucleotide field. To determine the therapeutic potential of silencing hepatocyte *Rnf10* in the treatment of MASLD, we designed GalNAc-si*Rnf10*. First, we designed 6 siRNAs to target mouse *Rnf10* mRNA and found that the sh*Rnf10*-2 sequence (atgagatgataatatgggg) effectively silenced both mouse and human *RNF10* mRNAs (Supplemental Figure 12, A–C). Functionally, distinct shRNA sequences targeting *Rnf10* consistently reduced lipid accumulation in hepatocytes (Supplemental Figure 12D). To increase stability, the 2′ OH position of ribose in si*Rnf10*-2 was chemically modified with 2′-*O*-methyl or 2′-fluoro groups. Additionally, the phosphodiester bonds were substituted with phosphorothioate bonds at the 5′ end of both the sense and antisense strands, as well as at the 3′ end of the antisense strands. These chemical modifications contributed to overall hydrophobicity and were essential for compound stabilization and efficient cellular internalization. Moreover, the 3′ end of the sense strand, which was conjugated to a trivalent GalNAc ligand, allowed targeted delivery to hepatocytes via the abundant, hepatocyte-specific and rapid recycling of asialoglycoprotein receptor (Supplemental Figure 12E).

Mice received a single subcutaneous injection of either GalNAc-si*NC* or GalNAc-si*Rnf10*-2 at doses of 0.1, 0.3, 1, 3, or 5 mg/kg. A high silencing efficiency was observed at a dose of 5 mg/kg GalNAc-si*Rnf10*-2 (Supplemental Figure 12F). In addition, mice received a single subcutaneous injection of either GalNAc-si*NC* or GalNAc-si*Rnf10*-2 and were analyzed at 3, 7, 14, 21, and 28 days after injection. Effective *Rnf10* silencing effects were observed for 21 days after injection (Supplemental Figure 12G). As expected, no reduction in RNF10 protein expression was observed in other tissues (fat, brain, kidney, pancreas, lung, spleen, intestine, testis, heart, and muscle) of GalNAc-si*Rnf10*-2–treated mice (Supplemental Figure 12H). The targeted delivery of modified si*Rnf10* by GalNAc to achieve *Rnf10* mRNA knockdown is depicted in Supplemental Figure 12I.

To assess the gene off-target effects of GalNAc-si*Rnf10*-2, we performed RNA-seq analysis of livers from GalNAc-si*NC*– or GalNAc-si*Rnf10*-2–treated mice (Supplemental Figure 13, A and B). Empirical cumulative distribution function analysis showed there were no strong gene off-target effects (Supplemental Figure 13C).

To assess the safety profile of GalNAc-si*Rnf10*-2, NCD-fed WT mice were administered vehicle, GalNAc-si*NC*, or GalNAc-si*Rnf10*-2. We observed that neither GalNAc-si*NC* nor GalNAc-si*Rnf10*-2 altered serum levels of AST, ALT, alkaline phosphatase, total bilirubin, blood urea nitrogen, or creatinine (Supplemental Figure 13D). Furthermore, neither GalNAc-si*NC* nor GalNAc-si*Rnf10*-2 altered cellular morphology, lipid accumulation, or immune cell infiltrations in the liver (Supplemental Figure 13E). These findings indicate that GalNAc-si*Rnf10*-2 exhibited no obvious systemic or hepatic toxicity.

**Figure 11:**
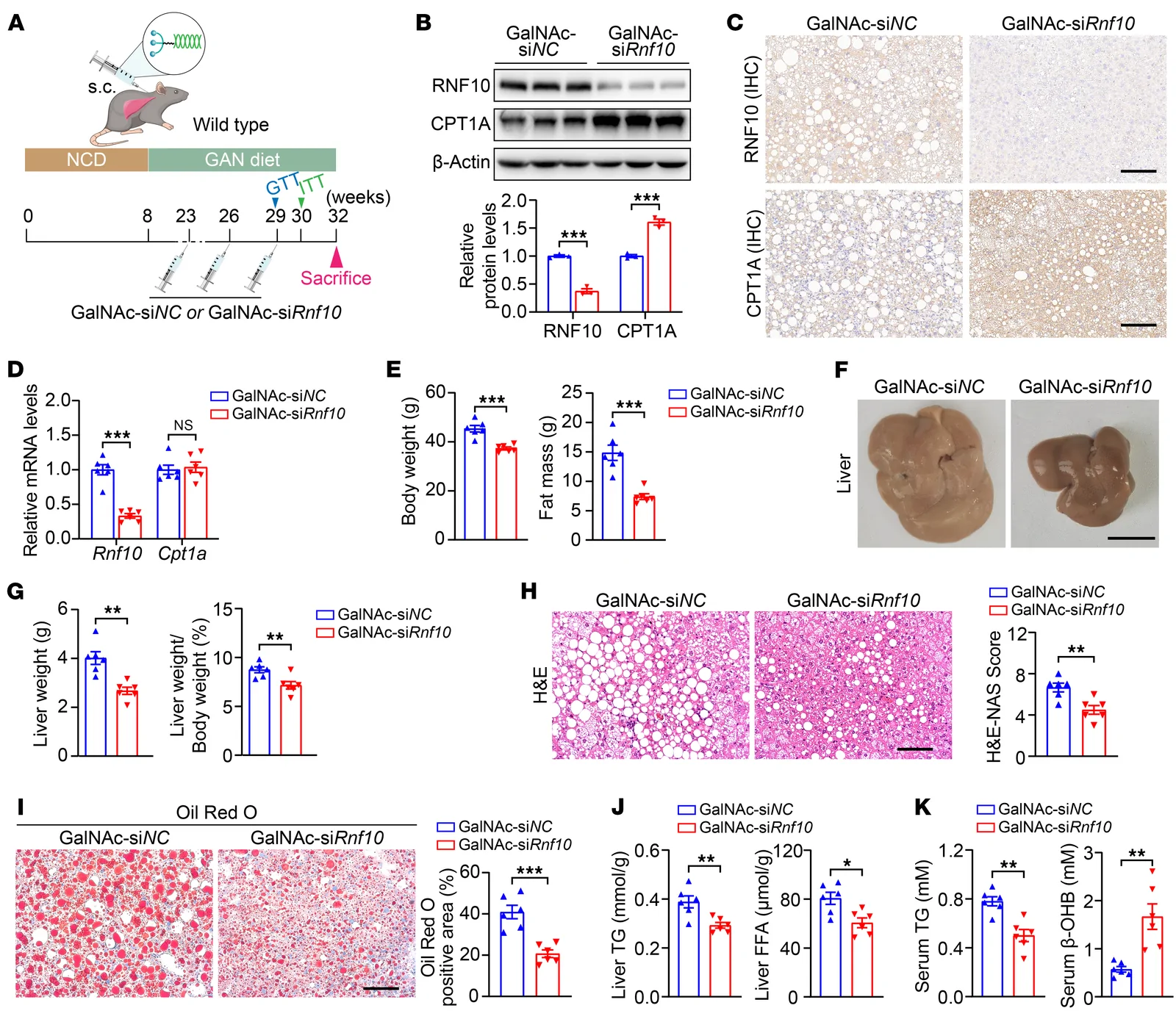
GalNAc-si*Rnf10*-2 targeting hepatic *Rnf10* ameliorates GAN diet–induced MASH in mice. (**A**) Treatment schedule for the GAN diet in mice, with an overview of GalNAc-si*Rnf10*-2 injection time points. Analyses were performed after 24 weeks of GAN diet feeding, and data are shown in **B**–**K**. (**B**) Relative protein levels of RNF10 and CPT1A (*n* = 3 mice/group). (**C**) Representative immunohistochemical staining of RNF10 and CPT1A. Scale bars: 50 μm. (**D**) Relative mRNA levels of *Rnf10* and *Cpt1a* (*n* = 6 mice/group). (**E**) Body weight and fat mass (*n* = 6 mice/group). (**F**) Gross liver morphology. Scale bar: 1 cm. (**G**) Liver weight and the LW/BW ratio (*n* = 6 mice/group). (**H** and **I**) Representative H&E (**H**) and Oil Red O (**I**) staining images and quantified results of the indicated groups (*n* = 6 mice/group). Scale bar: 50 μm. (**J** and **K**) Hepatic TG and FFA contents (**J**) and serum TG and β-OHB levels (**K**) (*n* = 6 mice/group). Data are presented as mean ± SEM. Unpaired 2-tailed Student’s *t* tests were performed to compare GalNAc-si*Rnf10*-2 with GalNAc-si*NC* groups. *P < 0.05, **P < 0.01, and ***P < 0.001.

**Figure 12:**
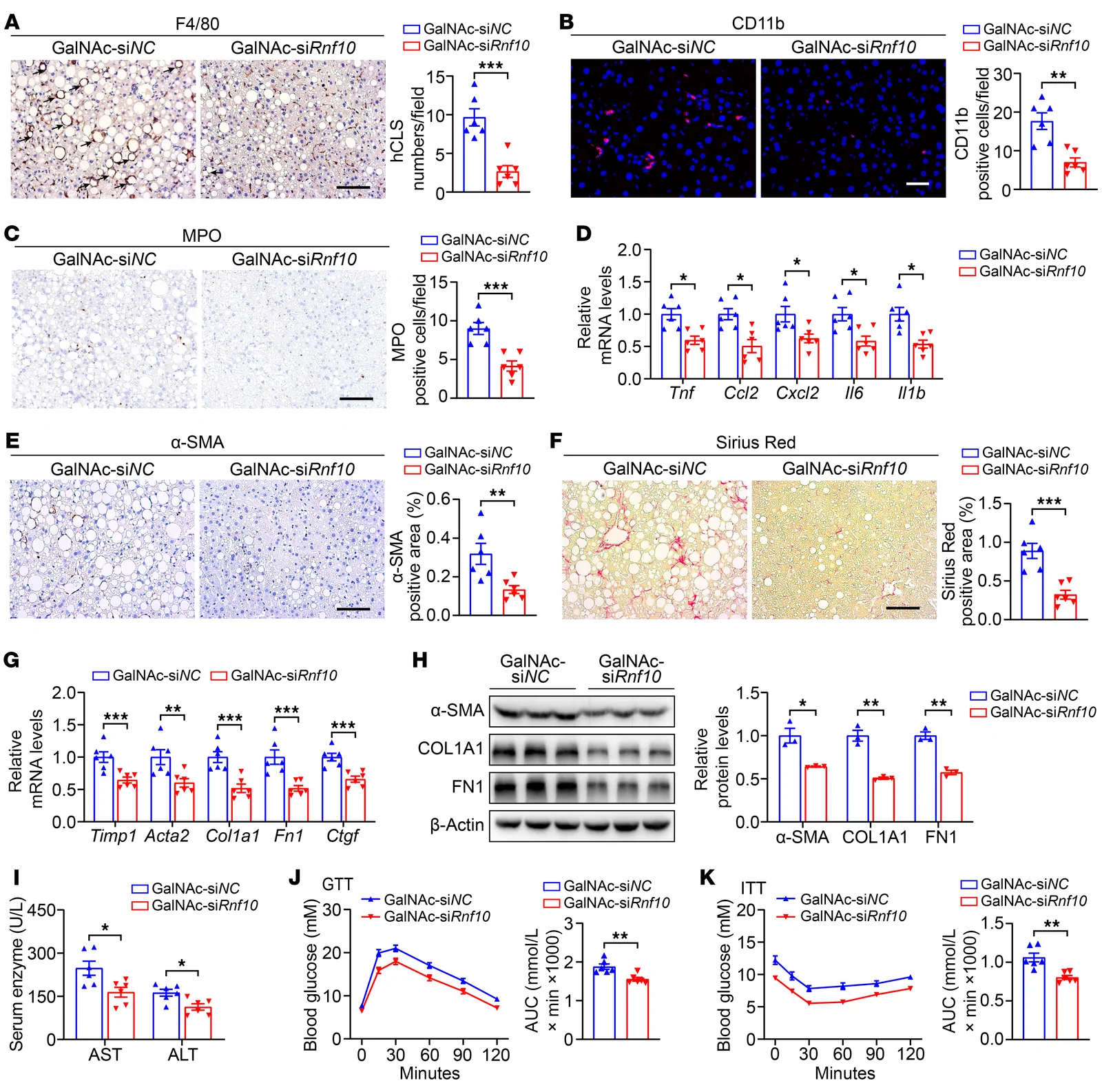
GalNAc-si*Rnf10*-2 targeting hepatic *Rnf10* ameliorates GAN diet–induced MASH in mice. (**A**–**C**) Representative images of F4/80^+^ immune cells by immunohistochemistry (**A**), CD11b^+^ immune cells by immunofluorescence (**B**), and MPO^+^ immune cells by immunohistochemistry (**C**), with quantification shown on the right (*n* = 6 mice/group). Scale bars: 50 μm. (**D**) Relative mRNA levels of genes related to proinflammatory cytokines (*n* = 6 mice/group). (**E** and **F**) Representative images of α-SMA immunohistochemical (**E**) and Sirius Red (**F**) staining, with quantification shown on the right (*n* = 6 mice/group). Scale bars: 50 μm. (**G** and **H**) Relative mRNA (**G**) (*n* = 6 mice/group) and protein (**H**) (*n* = 3 mice/group) levels of indicated genes related to liver fibrosis. (**I**) Serum AST and ALT levels (*n* = 6 mice/group). (**J** and **K**) GTT and ITT with corresponding AUC (*n* = 6 mice/group). Data are presented as mean ± SEM. Unpaired 2-tailed Student’s *t* tests were performed to compare GalNAc-si*Rnf10*-2 with GalNAc-si*NC* groups. *P < 0.05, **P < 0.01, and ***P < 0.001.

We next evaluated the therapeutic potential of GalNAc-si*Rnf10*-2 in MASLD. GAN diet–induced MASLD model mice were treated with GalNAc-si*NC* or GalNAc-si*Rnf10*-2 (Figure 11A). Consistent with our hypotheses, treatment with GalNAc-si*Rnf10*-2 increased CPT1A protein levels, whereas *Cpt1a* mRNA levels remained unchanged (Figure 11, B–D). Moreover, GalNAc-si*Rnf10*-2–treated mice presented a reduced body weight, fat mass, liver size, liver weight, and LW/BW ratio (Figure 11, E–G). Histological analysis revealed reduced hepatic steatosis and NAS in GalNAc-si*Rnf10*-2–treated mice (Figure 11, H and I). Biochemical assessments confirmed lower hepatic TG and FFA levels and lower serum TG levels, together with higher serum β-OHB levels, in GalNAc-si*Rnf10*-2–treated mice than in control mice (Figure 11, J and K). Additionally, GalNAc-si*Rnf10*-2–treated mice presented less inflammation, fibrosis, and liver injury (Figure 12, A–I). Furthermore, GalNAc-si*Rnf10*-2 treatment improved glucose tolerance and reduced insulin resistance (Figure 12, J and K).

Moreover, the administration of GalNAc-si*Rnf10*-2 to *ob/ob* mice reduced fat mass, liver weight, LW/BW ratio, NAS, and hepatic lipid droplet accumulation (Supplemental Figure 14, A–F). In addition, hepatic TG and FFA levels were decreased (Supplemental Figure 14G), along with lower serum TG, AST, and ALT levels and higher serum β-OHB levels (Supplemental Figure 14, H and I). Notably, GalNAc-si*Rnf10*-2 treatment also increased CPT1A protein levels without markedly altering *Cpt1a* mRNA levels (Supplemental Figure 14, J and K). Taken together, these findings suggest that GalNAc-si*Rnf10*-2 may be a promising therapeutic option for the treatment of MASLD.

## Discussion

The prevalence of MASLD is increasing globally, thus burdening public health (30, 31). Currently, treatment options for MASLD are limited (8, 32). Here, we found that RNF10 expression was significantly elevated in mouse models of MASLD. Moreover, RNF10 protein levels were positively correlated with the severity of MASLD in humans. Hepatocyte-specific *Rnf10* deletion markedly alleviated liver lipid accumulation, inflammation, and fibrosis. AAV-mediated *Rnf10* overexpression markedly aggravated MASLD-related phenotypes in mice. Consistently, GalNAc-mediated hepatocyte-specific *Rnf10* knockdown alleviated the MASLD phenotype in mice. Thus, strategies targeting RNF10 should be explored for the treatment of MASLD.

Impaired FAO has been implicated in the pathogenesis of fatty liver (33–35). Strategies that increase FAO, including the activation of FXR, PPARs, and THRβ, represent attractive therapeutic approaches for the treatment of MASLD (36–38). CPT1A is a rate-limiting enzyme in FAO, and its deficiency, an inborn metabolic disorder, is characterized by hepatic steatosis, inflammation, and an increased risk of liver failure (39–41). Numerous studies have shown that the activation of CPT1A can alleviate MASLD, highlighting CPT1A as a potential therapeutic target for MASLD treatment (15–17, 42). In addition, increasing evidence suggests that posttranslational modifications of CPT1A play important roles in liver lipid metabolism. For example, Softic and colleagues confirmed that a fructose-supplemented HFD leads to the increased acetylation of CPT1A, resulting in decreased FAO and contributing to MASLD pathogenesis (43, 44). In this study, we identified CPT1A as a direct target of RNF10 in the liver, which helps us better understand the mechanisms of MASLD and identify potential therapeutic options. Furthermore, we observed that hepatocyte-specific *Rnf10* deletion or overexpression altered body weight and fat mass in GAN diet– or HFD-fed mice. However, how hepatic RNF10 influences adipose phenotype remains unknown. Current evidence indicates that certain hepatokines, including FGF21 and GDF15, influence fat metabolism directly or indirectly (45, 46). Although CPT1A is a direct target of RNF10 and mediates the effects of RNF10 on hepatic FAO, it is not the sole target in liver. It is logical to speculate that certain hepatokines or metabolites that control fat metabolism might also be altered via other targets in mice with hepatic *Rnf10* deficiency or overexpression. Further studies are required to clarify this idea.

The members of the RING finger protein family to which RNF10 belongs have distinct associations with MASLD (21, 22, 47). With respect to RNF10, current studies have focused primarily on its role in modulating ribosome function through ubiquitylation of ribosomal proteins such as RPS2 and RPS3 (48–50). In this work, we elucidated the role of RNF10 in regulating FAO through the ubiquitination of CPT1A. Our in vitro experiments revealed that the domain encompassing amino acids 276–811 is the major fragment of RNF10 responsible for binding to CPT1A. Furthermore, the RNF10 RING domain mutant lacked E3 ligase activity to ubiquitinate CPT1A, highlighting the essential role of the RING finger domain in the ubiquitination function of RNF10. Additionally, the inhibitory effects of RNF10 on CPT1A greatly depend on its function in proteasomal ubiquitination, as evidenced by the inverse correlation between CPT1A protein abundance and RNF10 expression levels, which can be reversed with the proteasome inhibitor MG132. Importantly, different types of protein ubiquitination are known to serve specialized functions: K48-linked ubiquitination typically targets proteins for proteasomal degradation, K11-linked ubiquitination plays a more prominent role in regulating the cell cycle through complex regulatory mechanisms, and other linkages, such as K6, K27, K29, K33, and K63, are critical for regulating protein activity and the cellular signaling events responsible for DNA damage, cytokine activation, and other cellular processes (51–53). Our study demonstrated that RNF10 mediated the K48-linked ubiquitination of CPT1A. Therefore, interference with RNF10 could alleviate MASLD by regulating CPT1A K48-linked ubiquitination and preventing its proteasomal degradation.

E3 ubiquitin ligases in nonparenchymal cells have been implicated in the progression of liver inflammation and fibrosis. For example, Moreno-Lanceta et al. reported that RNF41 plays a critical role in regulating liver inflammation, fibrosis, and regeneration in macrophages (54). NEDD4L, another E3 ligase, has been shown to negatively regulate hepatic fibrogenesis by stimulating the degradation of the tyrosine kinase receptor TrkB in HSCs (55). In addition, Fondevila et al. reported that the inhibition of CPT1A ameliorated fibrosis by preventing the activation of HSCs (56). These findings suggest a functional role for RNF10 in nonparenchymal cells. In our study, we used HFD- and GAN diet–induced MASLD models to investigate the function of RNF10 in MASLD. Mice fed an HFD for 24 weeks develop hepatic steatosis with only mild inflammation, whereas approximately 1 year of feeding is needed for hepatic fibrosis to develop (57, 58). Thus, RNF10 may have a primary function in hepatocytes to inhibit FAO, which triggers lipotoxicity, hepatic inflammation, and fibrosis. Moreover, a GAN diet based on high fat, high fructose, and high cholesterol levels recapitulates features of metabolic syndrome and MASH with progressive fibrosis, representing an animal model that follows the natural course of human MASH development (59, 60). At present, we cannot exclude the possibility that RNF10 functions in nonparenchymal cells to synergistically regulate the progression of MASLD. We are continuing to investigate the functional role of RNF10 in HSCs and macrophages in liver disease.

GalNAc binds to the asialoglycoprotein receptor, which is highly expressed on hepatocytes, endowing GalNAc with the function of targeted delivery to the liver (61). Chemical modifications in the GalNAc-siRNA moiety protect siRNAs against nuclease digestion and immune recognition (62). Moreover, GalNAc-siRNAs can be delivered by subcutaneous administration, which can potentially allow for self-administration. Notably, a GalNAc-conjugated siRNA-based drug (givosiran) was approved by the FDA in 2019 for the treatment of acute hepatic porphyria (63). Since then, 4 other GalNAc-conjugated siRNA-based drugs have been approved for the treatment of liver diseases: lumasiran in 2020 and nedosiran in 2023 to treat primary hyperoxaluria type 1 (64, 65), inclisiran in 2020 for primary hypercholesterolemia (66), and vutrisiran in 2022 for hereditary transthyretin-mediated amyloidosis (67). Importantly, the GalNAc-based delivery of siRNAs targeting HSD17B13 or PNPLA3 has progressed to the preclinical stage for the treatment of MASH, highlighting the substantial potential of GalNAc-siRNA systems in MASH therapy (68–70). Here, we show that chemically modified si*Rnf10*-2 formulated with GalNAc effectively reduces liver steatosis, inflammation, and fibrosis in MASLD model mice. Thus, RNF10 is a potential therapeutic target for the treatment of MASLD.

There are several limitations in our study. First, only male mice were utilized for animal experiments. Whether RNF10 exerts similar effects on MASLD in female mice remains to be determined. Second, to develop RNF10-targeted therapeutic strategies for the treatment of MASLD, further studies are needed to uncover other potential roles of RNF10, including cell cycle and proliferation in the liver and other tissues. Lastly, although we observed a positive correlation between RNF10 protein levels and MASLD severity in patients, whether RNF10 is a safe and effective target for the treatment of human MASLD remains to be determined.

In summary, our findings reveal that RNF10 facilitates CPT1A degradation through K48-linked ubiquitination, inhibiting FAO and thereby aggravating the progression of MASLD. Moreover, GalNAc-si*Rnf10*-2 efficiently alleviated steatosis, inflammation, and fibrosis in a MASLD model. Thus, our work identifies RNF10 as a promising therapeutic target for the clinical treatment of MASLD.

## Methods

A detailed description of the methods used in this study is provided in the supplemental materials.

### Sex as a biological variable.

In the present study, only male mice were used because MASLD is a sex-dimorphic disease with a general higher prevalence in men, and estrogen is a key factor in MASLD progression. Whether the present findings could be applied to female mice still requires further study.

### Statistics.

Statistical methods are provided in the figure legends. For in vivo studies, littermates were randomly assigned to treatment groups for all experiments. Sample sizes were based on previous experiments with similar methodologies. The number of animals used is indicated in the figure legends. All animals were included in statistical analyses, and blinding was not performed. In cell culture experiments, data were obtained from 3 independent experiments. Data are presented as mean ± SEM. For comparisons between 2 groups, a 2-tailed unpaired Student’s *t* test was used. For comparisons between 3 or more groups, 1- or 2-way ANOVA was applied. Statistical analyses were performed using GraphPad Prism 9. A *P* value of less than 0.05 was considered statistically significant.

### Study approval.

Human sample collection and use complied with the Medical Ethics Committees of The Third People’s Hospital of Chengdu (approval number [2022]-S-62). The study protocol conforms to the principles outlined of the Declaration of Helsinki. Written informed consent was obtained from all participants or their families. All animal studies were approved by the Institutional Animal Care and Use Committee of Tianjin Medical University (permit number TMUaMEC2021052).

### Data availability.

Raw and processed RNA-seq data generated in this study have been deposited in the NCBI Gene Expression Omnibus under accession number GSE337995. Source data for all graphs are provided in the Supporting Data Values file. All other data supporting the findings of this study are available from the corresponding author upon reasonable request.

## Author contributions

CD, YZ, and HC contributed equally as co–first authors. The authorship order among the co–first authors was determined based on the extent of their overall contributions to the study. CD, YZ, and HC designed and performed the experiments and analyzed the data. YG, PL, and YC designed research. YZ, HC, CX, SS, KX, and WQ assisted with the experiments. YL and TZ provided human liver samples. CD and YC wrote the paper. All authors read and approved the manuscript.

## Conflict of interest

The authors have declared that no conflict of interest exists.

## Funding support

- National Natural Science Foundation of China grants 82530027 and 82230027 (to YC).
- National Key Research and Development Program of China grant 2022YFA0806102 (to YC).
- China Postdoctoral Science Foundation grant 2024M762378 (to CD).
- Discipline Research Special Project of Tianjin Medical University grant 2024XKNFM04 (to CD).
- The Tianfu Jincheng Laboratory (No. TFJCPI20250049).

## Supplementary Material

Supplemental data

Unedited blot and gel images

Supporting data values

## Version 1 08/11/2026

In-Press Preview

## Version 2 09/15/2026

Electronic publication
