# Supplementary material for: Targeting the E3 ubiquitin ligase RNF10 ameliorates metabolic dysfunction–associated steatohepatitis in mice
Source: J Clin Invest. 2026 Aug 6;136(18):e194148. doi: 10.1172/JCI194148 (PMC13574155; doi:10.1172/JCI194148)
Supplement: Supplementary file 1 [file jci-136-194148-s111.pdf]

Supplemental Methods

Patient samples

Liver biopsy samples were obtained from 32 patients with MASLD admitted to the Third People's Hospital of Chengdu. Clinical information, including sex, age, body mass index (BMI), and blood test results-such as aspartate aminotransferase (AST), alanine aminotransferase (ALT), total cholesterol (TC), triacylglycerol (TG), low-density lipoprotein (LDL), high-density lipoprotein (HDL), albumin, bilirubin, fasting glucose, platelet count, and prothrombin time-was documented. Patients with viral hepatitis A, B, or C, drug-related hepatitis, alcoholic or autoimmune liver disease were excluded. Patients with an alcohol consumption of >140 g/week in men and > 70 g/week in women were also excluded. For the control group, normal liver tissues were obtained from 13 patients who had liver tissues removed during cholecystectomy or treatment for blunt trauma. All control subjects had normal liver enzyme levels and less than 5% hepatic fat content. Two pathologists independently scored the NAFLD activity score (NAS) using the NASH-CRN (Clinical Research Network) system (1). Samples with a NAS of 1-2, no ballooning or fibrosis were classified in the steatosis group, while samples with a NAS greater than 4 or a NAS of 3-4 with fibrosis were categorized in the MASH group. Normal samples had a NAS of 0. All procedures involving human sample collection and application were approved by the Medical Ethics Committee of The Third People's Hospital of Chengdu and adhered to the principles outlined in the Declaration of Helsinki. Written informed consent was obtained from all participants or their families.

Animal study and approval

Male mice were utilized in this study. The mice were kept in a pathogen-free environment with a controlled 12-hour light-dark cycle. The temperature was maintained at 23 ± 2°C throughout the study. Food and water were available ad libitum. Male C57BL/6J mice, aged 6 to 8 weeks, were purchased from Beijing Vital River Laboratory Animal Technology (Beijing, China), while 4-week-old male *ob/ob* mice were obtained from Cyagen Biosciences (Suzhou, China). All animal procedures followed guidelines approved by the Institutional Animal Care and Use Committee of Tianjin Medical University.

Generation of *Rnf10*-Flox mice

Rnf10$^{flox/flox}$ mice were generated by Beijing Weishang Lide Biotechnology Co., Ltd. using the CRISPR/Cas9 system to flank the loxP sites to the second exon of *Rnf10* gene. To generate liver-specific *Rnf10* knockout mice (*Rnf10*-HKO), *Rnf10*$^{flox/flox}$ mice were mated with Albumin-Cre transgenic mice provided by Hongbing Zhang (Institute of Basic Medical Sciences, Peking Union Medical College). The Albumin promoter-driven Cre recombinase enabled targeted deletion of *Rnf10* specifically in hepatocytes. *Rnf10*$^{flox/flox}$ littermates (*Rnf10*-Flox) were used as controls in experiments. Genotyping for the *Rnf10*$^{flox/flox}$ and Albumin-Cre alleles was conducted via PCR analysis using specific primer pairs detailed in Supplemental Table 1.

Dietary modulation

To establish a fatty liver model, 8-week-old C57BL/6J mice were fed a high-fat diet (HFD; 60 kcal% fat; Research Diets Inc.; #D12492; New Brunswick, New Jersey) for 24 weeks. For the MASH model, C57BL/6J mice were fed a Gubra Amylin MASH diet (GAN; 40 kcal% fat, 20 kcal% fructose, and 2% cholesterol; Research Diets Inc.; #D09100310; New Brunswick, New Jersey) for 24 weeks or the L-amino acid diet (CDAHFD; 60 kcal% fat, 0.1% methionine, and no added choline; Research Diets Inc.; #A06071302; New Brunswick, New Jersey) for 7 weeks. Mice fed a normal chow diet (NCD; 10 kcal% fat, 20 kcal% protein, and 70 kcal% carbohydrate; Jiangsu Xietong Pharmaceutical Bio-engineering Co., Ltd.; #1010086; Jiangsu, China) served as controls. At the end of the feeding period, the whole liver and blood samples were collected for histological, cytological, biochemical, and molecular analysis.

Isolation and culture of mouse primary hepatocytes

Primary hepatocytes were isolated from *Rnf10*-Flox, *Rnf10*-HKO, and C57BL/6J mice (aged 6-8 weeks) using a well-established two-step collagenase perfusion via the portal vein, as previously described (2). Briefly, livers were perfused with 50 ml of Ca^{2+}- and Mg^{2+}-free HBSS supplemented with 20 mM HEPES, 10 mM $NaHCO_3$, and 2 mM EGTA (pH 7.4) at a controlled flow rate of 7 ml/min for 6 minutes, followed by HBSS with 0.05% (w/v) collagenase type II (Sigma-Aldrich; #C2-BIOC; St. Louis, MO, USA) at the same flow rate for 5 minutes. The HBSS was prewarmed to 37°C and infused with 95% O_2 and 5% CO_2. After perfusion, livers were excised, and hepatocytes were

flushed out and filtered through a 70 μm pore size mesh nylon filter. The cells were washed 3 times with precooled HBSS and centrifuged at 50 × g for 2 minutes at 4℃. Afterward, the cells were resuspended in RPMI 1640 medium (HyClone; #SH30027.02; Logan, UT, USA) and evaluated by Trypan blue exclusion to achieve more than 80% viable, then plated at the indicated density in culture plates. After 4-6 hours, unattached cells were washed away with PBS and fresh medium containing 10% FBS (HyClone; #SH30088.03; Logan, UT, USA) and 1% penicillin-streptomycin (Solarbio; #P1400; Beijing, China) was added for overnight incubation.

Cell culture and treatment

Human embryonic kidney (HEK) 293, 293T cell lines, and the human hepatoma cell line HepG2 were purchased from ATCC (#CRL-1573, #CRL-3216, and #HB-8065, respectively). The murine hepatocyte cell line AML12 was purchased from Pricella Life Science & Technology (#CL-0602; Wuhan, China). All cell lines were cultured in DMEM (HyClone; #SH30022.01; Logan, UT, USA) supplemented with 10% FBS and 1% penicillin-streptomycin and maintained at 37℃ with 5% CO_2. Mycoplasma contamination was tested and not detected. The MASLD cell model was established by treating HepG2 cells, AML12 cells, and primary hepatocytes with oleic acid (OA; 0.66 mM; Sigma-Aldrich; #O-1008; St. Louis, MO, USA) and palmitic acid (PA; 0.33 mM; Sigma-Aldrich; #P0500; St. Louis, MO, USA) (OA&PA) for 24 hours. Both PA and OA were dissolved in 0.5% fatty acid-free BSA (Solarbio; #A8850; Beijing, China), with fatty acid-free BSA used as the control.

Cellular oxygen consumption rate analysis

Oxygen consumption was measured at 37 ℃ using an XF24 Analyzer (Seahorse Bioscience) in accordance with the manufacturer's instruction. Oligomycin (2 μM), carbonyl cyanide-4-(trifluoromethoxy) phenylhydrazone (FCCP, 1.5 μM), and rotenone/antimycin (0.5 μM) were sequentially added to assess ATP-linked respiration and proton leak, maximal respiration, and nonmitochondrial respiration, respectively. Following the measurement, cell lysates were collected for total protein quantification.

Cellular Oil Red O staining and intracellular TG assay

To detect lipid accumulation, cells were stained with Oil Red O (Sigma-Aldrich; #00625; St. Louis, MO, USA). Briefly, cells were washed twice with PBS and fixed

with 4% paraformaldehyde. After fixation, they were rinsed twice with PBS and stained with 0.5% Oil Red O for 10 minutes. Next, the cells were rinsed with 60% isopropanol, washed twice with PBS, and then photographed. To measure intracellular TG content, a commercial kit (Applygen; #E1013; Beijing, China) was used following the manufacturer's instructions.

Plasmid construction

Full-length mouse *Rnf10* (NM_016698) and *Cpt1a* (NM_013495) were obtained by PCR from mouse liver cDNA, while human *RNF10* (NM_001330474) and *CPT1A* (NM_001876) coding sequences (CDS) were extracted from HepG2 cells. These CDS were inserted into the pcDNA3.1^{+} vector to generate overexpression plasmids for RNF10 and CPT1A, as well as cloned into the pCDH-CMV-MCS-EF1-copGFP vector (System Biosciences, #CD511B-1) for lentiviral production. To construct glutathione S-transferase (GST)-tagged HA-RNF10 and CPT1A, HA-tagged RNF10 and CPT1A were inserted into the prokaryotic expression vector pGEX-6p-1 (NovoPro Bioscience Inc., #V010912). Truncated variants of RNF10 (residues 1-215; 216-276; 277-811; 1-276; 216-811; △216-276) and CPT1A (residues 1-150; 151-756) were subcloned into the pcDNA5-Flag vectors. The RNF10 mutant in which Cys225 and Cys228 were substituted with serine (C225S/C228S) was cloned into the pcDNA5-HA vector. For knockdown, short hairpin RNA (shRNA) targeting mouse *Rnf10* (sh*Rnf10*) and *Cpt1a* (sh*Cpt1a*), as well as human *RNF10* (sh*RNF10*) and *CPT1A* (sh*CPT1A*), were constructs into the pLKO.1 vectors (Addgene, #10878). The control shRNA was cloned into the pLKO.1 vectors (sh*Ctrl*) and used as the negative control. These shRNA sequences are given in Supplemental Table 2.

Lentivirus production and infection

Lentivirus was produced by co-transfecting an overexpression or knockdown vector with packaging construct (psPAX2; Addgene, #12260) and envelope protein-producing plasmid (pMD2.G; Addgene, #12259) into HEK293T cells as previously described (3). Viral supernatants were collected 48 to 72 hours after transfection. After concentration, the viral was stored at -80℃ until use. For infections, cells were incubated with lentivirus in a medium containing 8 μg/mL polybrene for 24 hours before proceeding with further experiments.

Adenovirus production

Mouse *Rnf10*-overexpressing recombinant adenovirus (Ad-*Rnf10*) was produced by WZ Biosciences *Inc.* (Shandong, China) and amplified in HEK293 producer cells.

Adeno-associated virus (AAV) production and delivery

To express reconstituted proteins, plasmids were engineered from the pAAV-TBG-*GFP* vector (Addgene, #105535) by replacing the *GFP* coding sequence with *Rnf10* or *Cpt1a*. For liver-specific *Cpt1a* knockdown, a shRNA targeting *Cpt1a* with miR30 flanking sequences was cloned into the pAAV-TBG-*GFP* vector, generating the AAV-TBG-mir30-sh*Cpt1a* construct (Figure 5A). Expression of RNF10, CPT1A or sh*Cpt1a* was driven by the human thyroxine-binding globulin (TBG) promoter. Recombinant AAV particles were produced by co-transfecting overexpression or knockdown vector with Rep/Cap (2/8) plasmid (pAAV2/8; Addgene, #112864) and helper plasmid (pAdDeltaF6; Addgene, #112867). Viral particles were harvested 72 hours post-transfection, purified using an iodixanol gradient, and concentrated with a 100-kDa centrifugation filter (Sartorius Stedim Lab Ltd.; #VS2042; Stonehouse, UK). AAV titers were quantified by qPCR, adjusted to 0.5×10^{10} viral genomes (vg)/μL in PBS, and administered via tail vein injection (200 μL per mouse) at the indicated point.

Glucose and insulin tolerance tests

For the glucose tolerance test, mice were fasted for 16 hours, then injected intraperitoneally with glucose at a dose of 2 g/kg body weight. For the insulin tolerance test, mice were fasted for 6 hours and then injected intraperitoneally with 1 unit of insulin (Sigma-Aldrich; #PHR8925; St. Louis, MO, USA) per kg body weight. Blood glucose levels were measured from the tail vein at 0, 15, 30, 60, and 120 minutes using a glucometer (One Touch Ultra; LifeScan; Milpitas, CA).

Measurement of metabolic indices and liver function

Serum TG, AST, ALT, alkaline phosphatase (ALP), total bilirubin (TBIL), blood urea nitrogen (BUN), and creatinine (Cr) were determined using an automatic biochemical analyzer (AU5800; Beckman Coulter Chemistry System analyzer). Hepatic TG was measured with a colorimetric diagnostic kit (Applygen Technologies; #E1013; Beijing, China). Serum β-Hydroxybutyrate levels were measured using the β-Hydroxybutyrate

(Ketone Body) Colorimetric Assay Kit (Cayman Chemical; #700190; Ann Arbor, MI). Free fatty acid (FFA) in the liver was quantified using the Free Fatty Acids Quantitation Kit (Solarbio Life Sciences; #BC0595; Beijing, China).

Histological analyses

Liver tissues were fixed overnight in 4% formaldehyde and then embedded in paraffin. H&E staining was used to assess lipid accumulation and inflammation. Frozen liver sections were prepared with Tissue-Tek OCT compound, and Oil Red O staining was used to see lipid droplets. Sirius Red staining was used to check for liver fibrosis. A light microscope (ZEISS; Oberkochen, Germany) was used to observe the tissue features, and images were taken. The NAFLD activity score (NAS) was used, including liver steatosis (scale of 0-3), lobular inflammation (scale of 0-3), and hepatocellular ballooning (scale of 0-2) (4).

Western blot analysis

Tissues and cells were collected and lysed using RIPA lysis buffer (150 mM NaCl, 50 mM Tris-HCl pH 7.5, 1% (w/v) sodium deoxycholate, 1% (w/v) SDS, 1% (v/v) Triton X-100) supplemented with 1mM PMSF (#P0100; Solarbio; Beijing, China) and cOmplete Protease Inhibitor Cocktail (Roche; #04693116001; Basel, Switzerland). After centrifugation, the supernatant was collected and the total protein concentration was quantified using a BCA protein quantification kit (GenStar; #E162-101; Beijing, China). Equal amounts of protein were separated by 10% or 12% SDS-PAGE and transferred onto PVDF membranes (Millipore; #IPVH00010; Billerica, MA, USA). The PVDF membranes were blocked with TBST containing 5% skim milk and incubated with primary antibodies overnight at 4°C. After incubated with corresponding resistant secondary antibodies, protein levels were detected using the ECL kit (GenStar; #ZE171-101; Beijing, China) and visualized in a Tanon 5200 Imaging System (Beijing YuanPingHao Biotech; Beijing, China). The antibodies are listed in Supplemental Table 3.

Immunofluorescence (IF)

For immunofluorescence staining of RNF10 and CPT1A, cells grown on coverslips were incubated with primary antibody overnight at 4°C, followed by fluorophore-conjugated secondary antibody (Alexa Flour 594 AffiniPure goat anti-Rabbit IgG

(H+L); Yeasen; #33112ES60; Shanghai, China). For CD11b immunofluorescence staining, paraffin-embedded liver sections were processed using the same procedure. The primary antibodies are listed in Supplemental Table 3. Nuclei were stained with DAPI (Beyotime; #C1005; Shanghai, China). Immunofluorescence images were captured using a fluorescence microscope (NIKON ECLIPSE CI, Nikon, Japan).

Immunohistochemistry (IHC)

Paraffin-embedded liver sections were prepared for immunostaining. Endogenous peroxidase activity was blocked by incubating the sections in 3% H_2O_2 for 30 min at 37°C. The sections then underwent antigen retrieval in a microwave and were blocked with 5% BSA for 1 hour at room temperature. The primary antibodies are listed in Supplemental Table 3. After incubation with horseradish peroxidase (HRP)-conjugated secondary antibodies, the sections were visualized using 3, 3'-Diaminobenzidine (MedChemExpress; #HY-15912; Monmouth Junction, NJ, USA). The slides were stained with hematoxylin, dehydrated, and mounted for observation under a light microscope (ZEISS, Oberkochen, Germany).

RNA extraction and qPCR

Total RNA was extracted from tissues or cells using Triquick Reagent (Solarbio; #R1100; Beijing, China) according to the manufacturer's instructions. RNA concentration was measured using a NanoDrop 2000C spectrophotometer (Thermo Fisher Scientific, Waltham, MA, USA). For quantitative real-time PCR, 2 μg of total RNA was reverse-transcribed using the Applied Biosystems High-Capacity cDNA Reverse-Transcription Kit (Thermo Fisher Scientific; #4374967; Waltham, USA). SYBR Green PCR Master Mix (Promega; #A6001; Madison, USA) was used along with 500 nM primers, following the manufacturer's instructions. The PCR reactions were performed with a LightCycler Nano system (Roche; Basel, Switzerland). Relative gene expression was calculated using the comparative Ct ($2^{-\Delta\Delta Ct}$) method, normalizing to β-Actin. Primer sequences are listed in Supplemental Table 4.

Mass spectrometry analysis and data processing

Primary hepatocytes isolated from C57BL/6J mice were transiently infected with Ad-*Rnf10*-Flag for 12 hours, followed by treatment with OA&PA for an additional 24 hours. Cell lysates were prepared with IP lysis buffer supplemented with a protease inhibitor

cocktail (Roche; Basel, Switzerland). Flag-tagged protein complexes were captured using anti-Flag agarose (Sigma-Aldrich; #A2220; St. Louis, MO, USA). After binding, the beads were washed six times with a washing buffer. Flag peptide (Sigma-Aldrich; #F3290; St. Louis, MO, USA) was used to elute the protein complexes. Primary hepatocytes infected with Ad-*GFP*-Flag were used as the control. The eluted complexes were separated on SDS-PAGE gels and visualized using ProteoSilver Stain for Mass Spectrometry (Sigma-Aldrich; #PROTSIL1; St. Louis, MO, USA). Protein bands of interest were excised and examined using a Nano-LC system (EASY-nLC 1200, Thermo Fisher Scientific) coupled with an Orbitrap Q-Exactive Plus mass spectrometer (Thermo Fisher Scientific). The resulting MS/MS data were processed using Proteome Discoverer software (version 1.4).

Co-immunoprecipitation (Co-IP) assays

Co-IP assays were performed in hepatocytes or HEK293T cells transiently transfected with the indicated plasmids for 24 hours. Endogenous Co-IP was performed in hepatocytes. Cells were lysed with IP lysis buffer (50 mM Tris-HCl, pH 7.5; 150 mM NaCl; 1 mM EDTA; 1% (v/v) NP-40), and the lysates were incubated with constant agitation and then centrifuged at 4°C. The supernatants were incubated with the indicated antibody and Protein G-agarose beads overnight at 4°C. The beads were washed six times with cold washing buffer (20 mM Tris-HCl, pH 8.0; 500 mM NaCl; 1 mM EDTA; 0.1% (v/v) NP-40) and resuspended in SDS buffer, followed by heating at 100°C for 10 minutes. The samples were analyzed by Western blot.

Glutathione S-transferase (GST) precipitation assays

In GST pull-down assays, Flag-RNF10 and CPT1A proteins were overexpressed in HEK293T cells and lysed with IP lysis buffer (50 mM Tris-HCl, pH 7.5; 150 mM NaCl; 1 mM EDTA; 1% (v/v) NP-40), then immunopurified using anti-Flag antibodies. GST-RNF10 and CPT1A proteins were purified from HA-GST- RNF10 or CPT1A plasmid-transfected *Escherichia coli* BL21 (DE3). The cells were induced with 1 mM isopropyl-β-D-thiogalactopyranoside (IPTG; MedChemExpress; #HY-15921; Monmouth Junction, NJ, USA) for 12 hours and lysed with lysis buffer (50 mM Na_2HPO_4, pH 8.0; 300mM NaCl; 1% Triton X-100; Protease Inhibitor Cocktail). The HA-GST tagged protein extracts were incubated with GST-Sefinose™ Resin 4FF (MedChemExpress; #C600031; Monmouth Junction, NJ, USA) for 3 hours at 4°C. The GST beads loaded

with proteins were incubated overnight at 4°C with purified Flag-tagged RNF10 or CPT1A protein. After washing six times with washing buffer (20 mM Tris-HCl, pH 8.0; 500 mM NaCl; 1 mM EDTA; 0.1% (v/v) NP-40), the beads were resuspended in 2× SDS loading buffer and heated at 100°C for 10 minutes. The samples were analyzed by Western blot.

Confocal microscopy

To study the colocalization of RNF10-Flag and CPT1A-HA, HepG2 cells were infected with lentiviruses overexpressing the indicated genes for 24 hours. The cells were fixed with 4% paraformaldehyde for 15 minutes and permeabilized with 0.2% Triton X-100 for 10 minutes. Cells were incubated overnight at 4°C with mouse anti-Flag (MBL; #M185-3; Dilution: 1:100) or rabbit anti-CPT1A (Proteintech; #15184-1-AP; Dilution: 1:100). The fluorophore-conjugated secondary antibodies were incubated for 2 hours at room temperature. Cell nuclei were stained with DAPI for 10 min. Images were taken using a laser-scanning confocal microscope (Olympus FV1200).

In vivo ubiquitination assays

For ubiquitination assays, murine primary hepatocytes or HEK293T cells were co-transfected with the indicated plasmids, whereas HepG2 cells were infected with lentiviruses encoding the indicated genes. After 24 hours, the cells were treated with MG132 (MedChemExpress; #HY-13259; Monmouth Junction, NJ, USA) at a final concentration of 20 μM for an additional 12 hours. The cells were then lysed with 100 μl SDS lysis buffer (50 mM Tris-HCl, pH 7.5; 150 mM NaCl; 1 mM EDTA; 1% (w/v) SDS), mixed with a protease inhibitor cocktail, and heated at 100°C for 10 min. The supernatants were diluted 10-fold with lysis buffer (50 mM Tris-HCl, pH 7.5; 150 mM NaCl; 1 mM EDTA; 1% (v/v) NP-40) containing Protease Inhibitor Cocktail. After centrifugation, the supernatants were subjected to immunoprecipitation with the indicated antibodies. Western blot was performed to measure the ubiquitin chains using anti-Ub or anti-His antibodies.

Cycloheximide assay

Murine primary hepatocytes or HepG2 cells were cultured in 6-well plates overnight. Murine primary hepatocytes were infected with adenoviruses Ad-*GFP* or Ad-*Rnf10*, and HepG2 cells were infected with lentiviruses expressing either control or Flag-

RNF10. After 24 hours, the cells were treated with 100 μg/ml cycloheximide (CHX; MedChemExpress; #HY-12320; Monmouth Junction, NJ, USA) dissolved in DMSO to block protein synthesis. Total protein lysates were harvested at specified time points and analyzed by Western blot using the indicated antibodies.

GalNAc-si*Rnf10*-2 synthesis

To identify effective siRNA sequences for silencing *Rnf10* in vivo, six test sequences targeting different regions of mouse *Rnf10* mRNA were designed (Supplemental Table 2). The sequence with the strongest silencing effect in both mouse and human *RNF10* mRNA was selected: sh*Rnf10*-2. GalNAc-modified si*Rnf10*-2 (sense: 3' c-c-cuaaGaAGAucaaccugaaL 5', antisense: 3' u-U-cagGuuGaucuUcUuaggg-c-c 5') was provided by Biosyntech and administered via subcutaneous injection at different doses in the indicated times to male C57BL/6J mice. Mice treated with GalNAc-si*NC* (sense: 3' u-u-cuCcGAAcgugucacguL 5', antisense: 3' a-C-gugAcACguucGgAgaa-u-u 5') served as the control. The capital letter represents 2'-deoxy-2'-fluoro (2'-F) modifications; the lowercase letter represents 2'-*O*-methyl (2'-OMe) modifications; "-" represents phosphorothioate (PS) linkages; "L" represents triantennary *N*-acetylgalactosamine (GalNAc).

RNA-sequencing

Total RNA from liver tissues was extracted using TRIzol Reagent. RNA concentration and quality were assessed with NanoDrop. RNA-sequencing libraries were prepared following Illumina Stranded mRNA Prep, Ligation (San Diego, CA) using 1 μg of total RNA. Paired-end cDNA libraries were sequenced on a DNBSEQ platform using the DNBSEQ Reagent Kit (FCL PE150, version 3.0) with a read length of 150 bp. The reads were mapped to the Ensembl mouse reference genome (mm10/GRCm38) by HISAT2 software. DESeq2 (version 1.50.2) was used for differential gene expression analysis. To evaluate GalNAc-si*Rnf10*-2 off-target effects, we performed seed match enrichment analysis using the R package SeedMatchR (5). Genes containing 7-8 nucleotide seed matches (mer7-mer8) to the si*Rnf10*-2 guide strand were identified, and their expression changes were compared with background genes. ECDF curves of $\log_2$(fold chang) were generated, and statistical significance was assessed using the Kolmogorov-Smirnov test.

Supplemental Figures and Tables

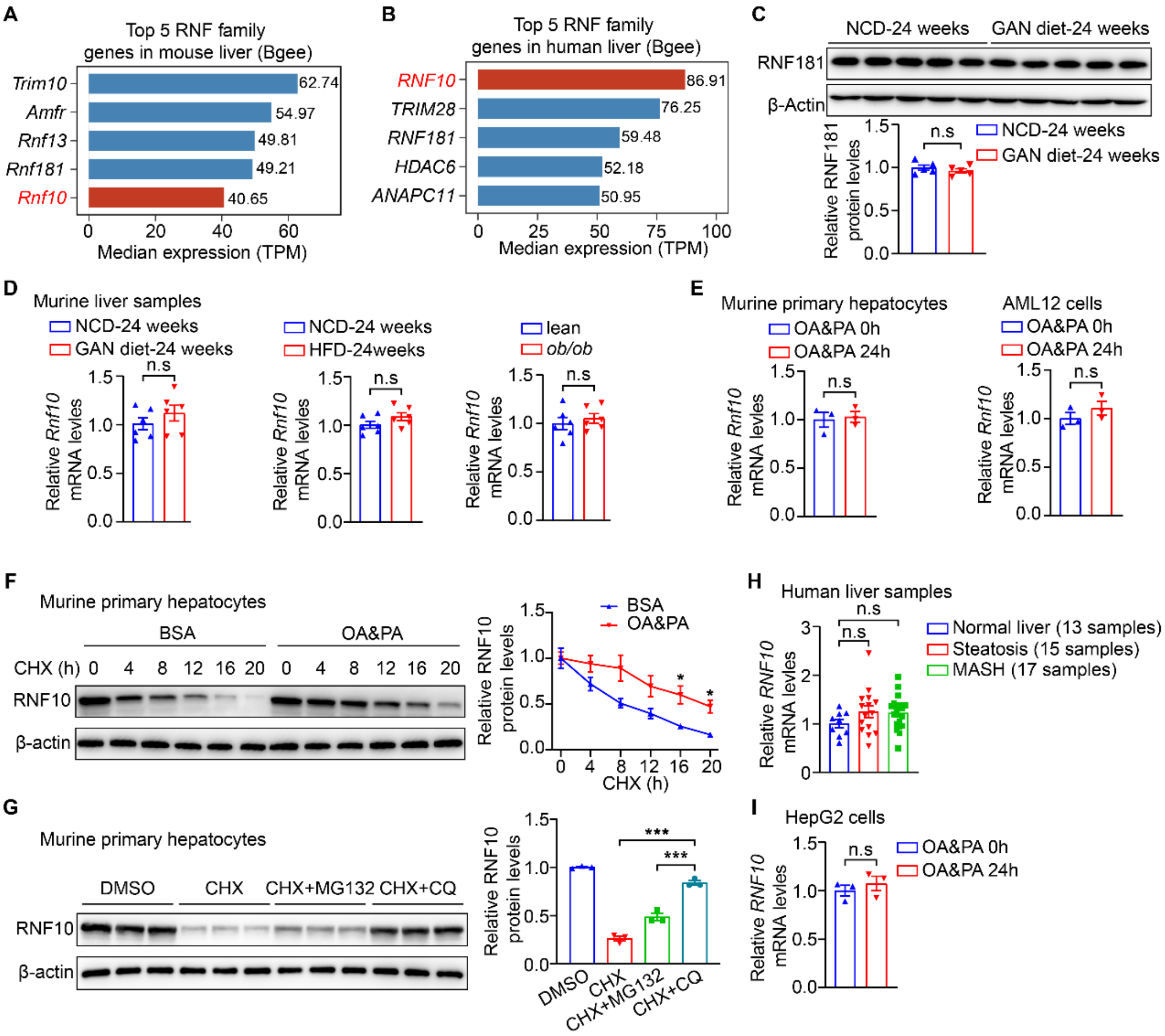

Supplemental Figure 1. *RNF10* mRNA expression remains unchanged in MASLD, whereas its protein stability is increased through the lysosomal pathway, related to Figure 1. (**A** and **B**) Bar plots showing the top 5 RNF family genes in murine and human liver from the Bgee database, ranked by median expression level (TPM). *Rnf10*/*RNF10* is highlighted in red. (**C**) Relative RNF181 protein levels in the livers of mice fed an NCD or GAN diet for 24 weeks (n = 5 mice/group). (**D**) Relative *Rnf10* mRNA levels in mouse liver samples from mice fed an NCD, HFD or GAN diet for 24 weeks, and in lean and *ob/ob* mice (n = 6 mice/group). (**E**) Relative *Rnf10* mRNA levels in murine primary hepatocytes and AML12 cells treated with OA&PA for 24 h (n = 3 independent experiments). (**F**) Relative RNF10 protein levels in murine primary hepatocytes treated with BSA or OA&PA, followed by CHX (100 μg/ml) treatment. Quantitative results are shown on the right side (n = 3 independent experiments). (**G**) Murine primary hepatocytes were treated with DMSO, CHX alone, or CHX together

with MG132 or CQ, followed by Western blot analysis of RNF10. Quantitative results are shown on the right side (n = 3 independent experiments). (**H**) Relative *RNF10* mRNA levels in liver tissue from individuals with normal liver, with steatosis, or with MASH. (**I**) Relative *RNF10* mRNA levels in HepG2 cells treated with OA&PA for 24 h (n = 3 independent experiments). Data are presented as mean ± SEM. Unpaired 2-tailed Student's *t*-test were performed in (**C–F** and **I**) and 1-way ANOVA was performed in (**G** and **H**). $^{*}P < 0.05$ and $^{***}P < 0.001$.

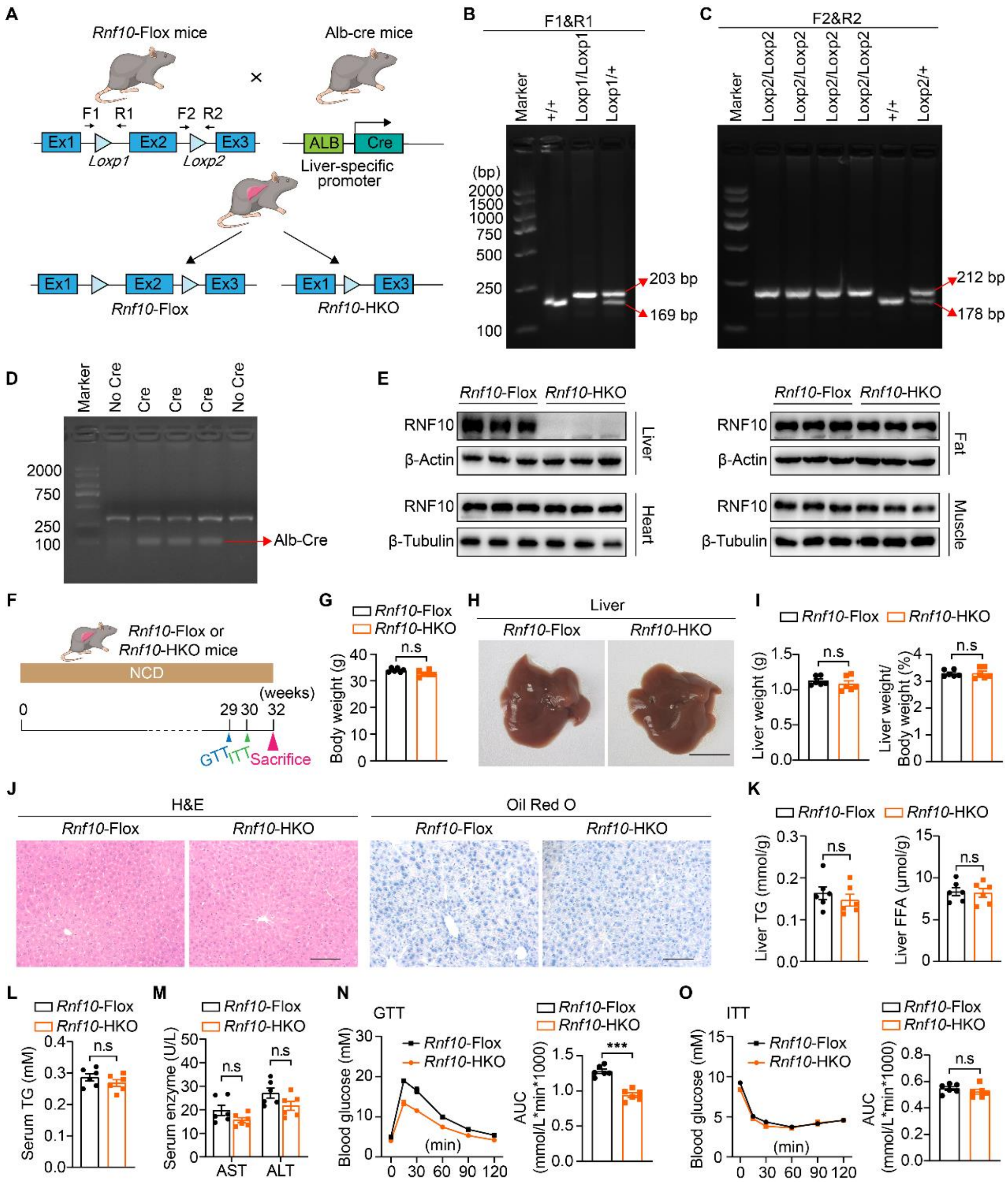

Supplemental Figure 2. Generation and characterization of hepatic-specific *Rnf10*-deficient (*Rnf10*-HKO) mice, related to Figure 2. (**A**) Workflow for generating *Rnf10*-HKO mice. (**B–D**) Mouse genotypes were identified by gel electrophoresis. (**E**) Relative RNF10 protein levels in different tissues (liver, fat, heart, and muscle) (n = 3 mice/group). (**F**) Schematic showing NCD feeding regimen of *Rnf10*-Flox and *Rnf10*-HKO mice for the data shown in (G–O). *Rnf10*-Flox mice were used as controls. Mice were fed a NCD for 32 weeks. (**G**) Body weight in indicated groups (n = 6 mice/group). (**H**) Gross liver morphology. Scale bar, 1 cm. (**I**) Liver

weight and the LW/BW ratio (n = 6 mice/group). (**J**) Representative H&E and Oil Red O staining images, with quantification shown on the right (n = 6 mice/group). Scale bar, 50 µm. Hepatic triglyceride (TG) and free fatty acid (FFA) contents (**K**), serum TG, AST, and ALT levels (**L** and **M**) (n = 6 mice/group). (**N** and **O**) Glucose tolerance test (GTT) and insulin tolerance test (ITT) with the corresponding area under the curve (AUC) (n = 6 mice/group). Data are represented as mean ± SEM. Unpaired 2-tailed Student's *t*-test were performed comparing *Rnf10*-HKO with *Rnf10*-Flox groups. ***$P < 0.001$.

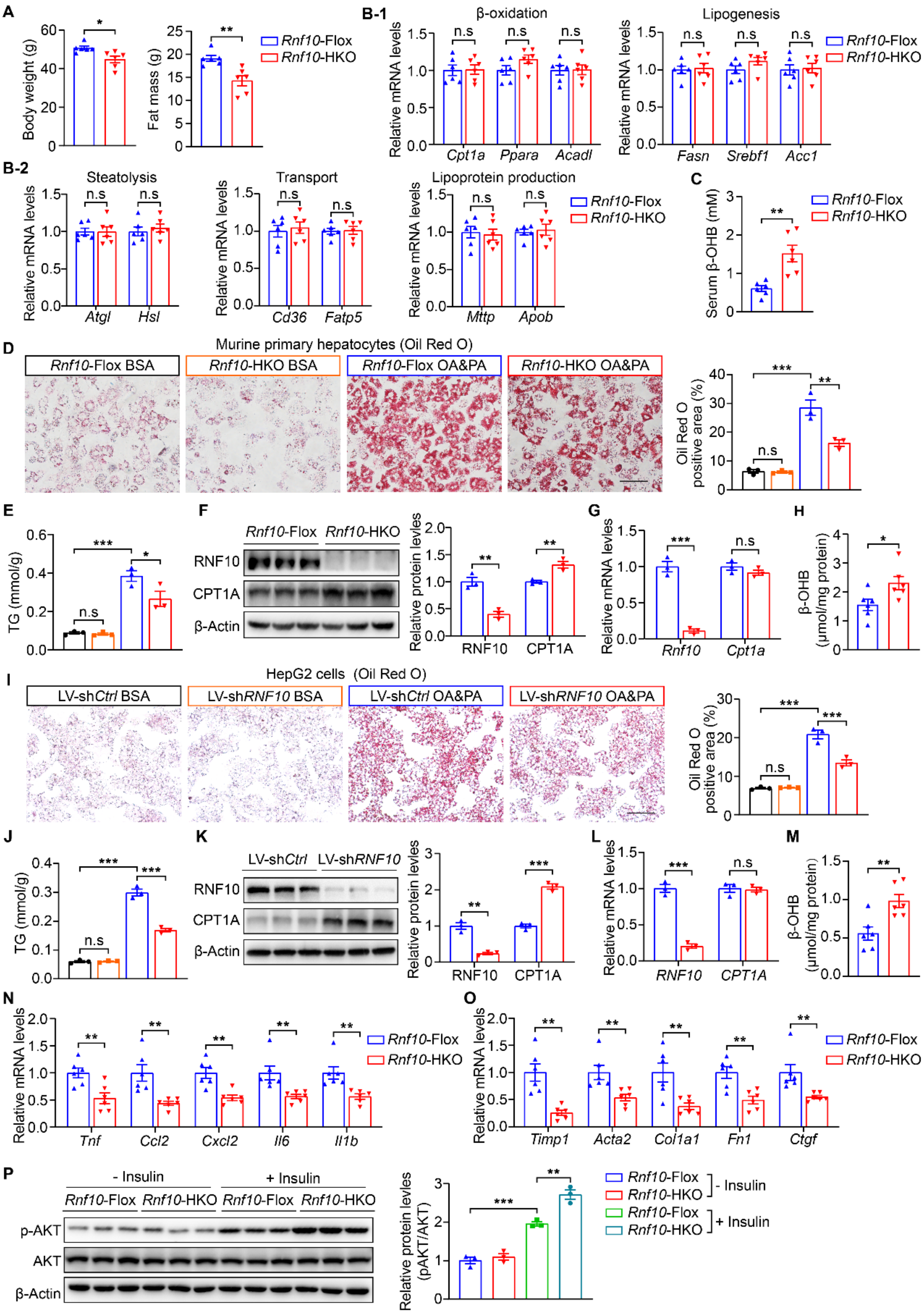

Supplemental Figure 3. *RNF10* downregulation increased CPT1A protein and β-hydroxybutyrate (β-OHB) levels without affecting *CPT1A* mRNA expression, related to Figure 2. (**A**) Body weight and fat mass of *Rnf10*-Flox and *Rnf10*-HKO mice (n = 6 mice/group). (**B**) Relative mRNA levels of lipid metabolism markers in the liver

(β-oxidation, lipogenesis, steatolysis, fatty acid transport and lipoprotein production) (n = 6 mice/group). (**C**) Serum β-OHB levels in the indicated groups (n = 6 mice/group). Representative Oil Red O staining images (**D**) and intracellular TG contents (**E**) (n = 3 independent experiments) in the indicated groups in murine primary hepatocytes. Relative protein (**F**) and mRNA (**G**) levels of RNF10 and CPT1A in murine primary hepatocytes from the indicated groups (n = 3 independent experiments). (**H**) β-OHB levels in the culture medium of murine primary hepatocytes from the indicated groups (n = 6 independent experiments). Representative Oil Red O staining images (**I**) and intracellular TG contents (**J**) (n = 3 independent experiments) in the indicated groups in HepG2 cells. Relative protein (**K**) and mRNA levels (**L**) of RNF10 and CPT1A in HepG2 cells from the indicated groups (n = 3 independent experiments). (**M**) β-OHB levels in the culture medium of HepG2 cells from the indicated groups (n = 6 independent experiments). (**N**) Relative hepatic mRNA expression levels of genes related to pro-inflammatory cytokines (n = 6 mice/group). (**O**) Relative mRNA levels of indicated genes related to liver fibrosis (n = 6 mice/group). (**P**) Relative protein levels of phosphorylated AKT in liver tissues from the indicated groups (n = 3 mice/group). Data are presented as mean ± SEM. Unpaired 2-tailed Student's *t*-test were performed in (**A–C**, **F–H**, and **K–O**) and 1-way ANOVAs were performed in (**D**, **E**, **I**, **J**, and **P**). *$P < 0.05$, **$P < 0.01$, and ***$P < 0.001$.

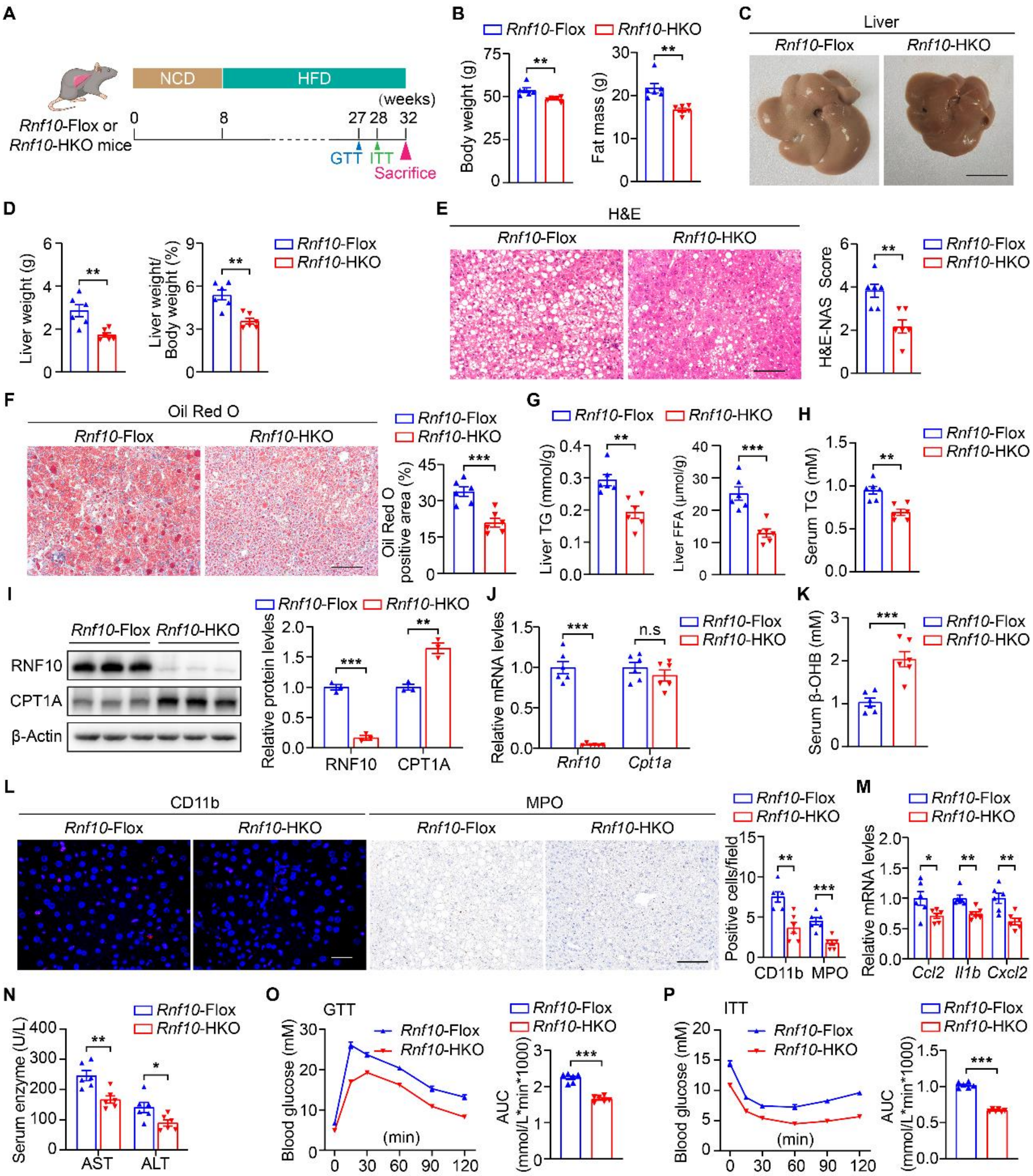

Supplemental Figure 4. Hepatic-specific *Rnf10* deficiency ameliorates HFD-induced liver lipid accumulation and inflammation, related to Figure 2. (**A**) Schematic of HFD-fed *Rnf10*-Flox and *Rnf10*-HKO mice for the data shown in (B–P). Mice were fed an HFD for 24 weeks. (**B**) Body weight and fat mass (n = 6 mice/group). (**C**) Gross liver morphology. Scale bar, 1 cm. (**D**) Liver weight and the LW/BW ratio (n = 6 mice/group). Representative H&E (**E**) and Oil Red O (**F**) staining images of liver sections and quantified results (n = 6 mice/group). Scale bar, 50 μm. Levels of hepatic TG and FFA (**G**), serum TG (**H**) (n = 6 mice/group). Relative protein (**I**) (n = 3 mice/group) and mRNA (**J**) (n = 6 mice/group) levels of RNF10 and CPT1A, and serum β-OHB levels (n = 6 mice/group) from *Rnf10*-Flox and *Rnf10*-HKO mice (**K**) fed an

HFD for 24 weeks. Representative images and quantification of CD11b immunofluorescent staining and MPO immunohistochemical staining (**L**), and relative mRNA levels of proinflammatory genes (**M**) (n = 6 mice/group). Scale bar, 50 μm. (**N**) Serum AST and ALT levels (n = 6 mice/group). (**O** and **P**) GTT and ITT with the corresponding AUC (n = 6 mice/group). Data are represented as mean ± SEM. Unpaired 2-tailed Student's *t*-test were performed comparing *Rnf10*-HKO with *Rnf10*-Flox groups. *$P < 0.05$, **$P < 0.01$, and ***$P < 0.001$.

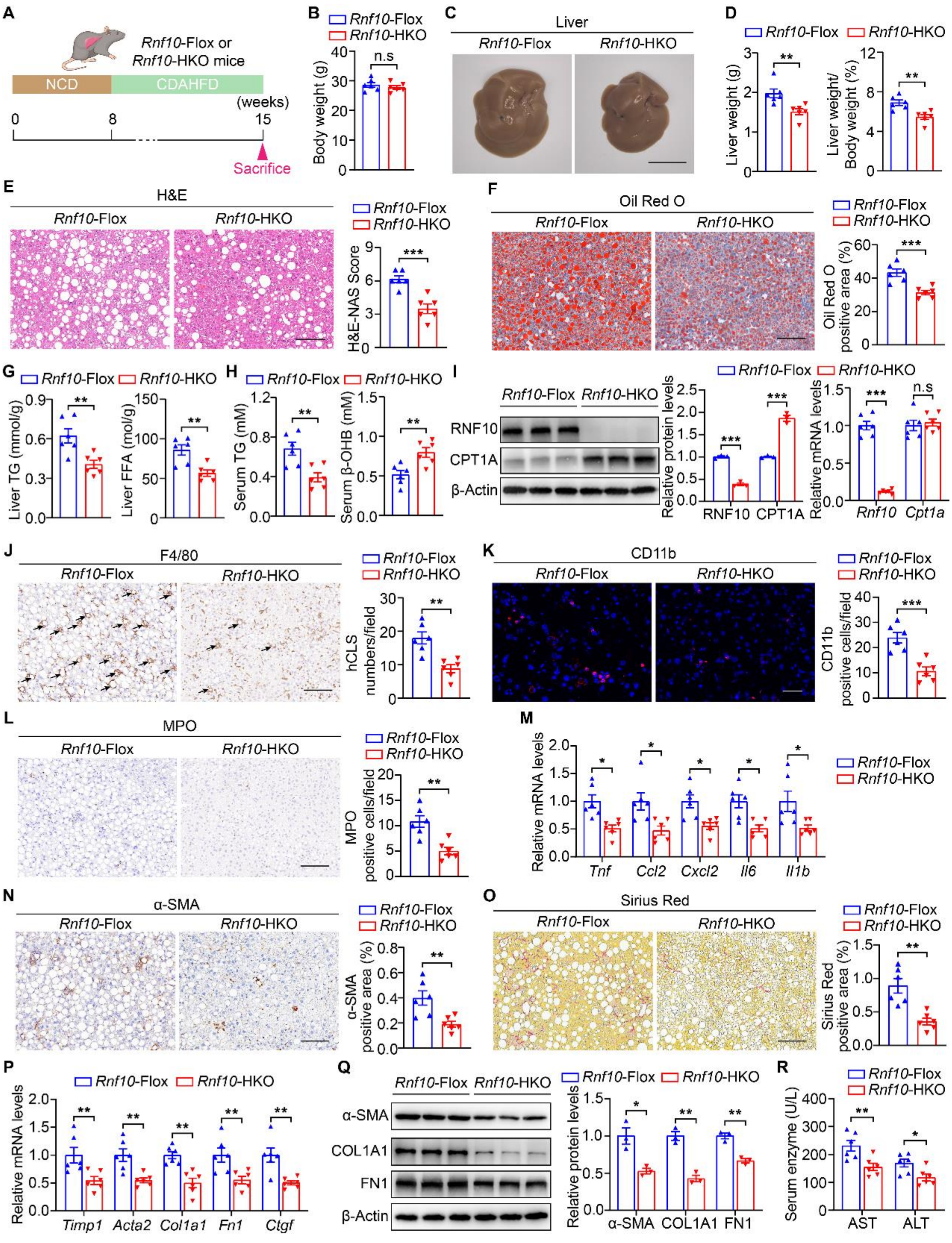

Supplemental Figure 5. Hepatic-specific *Rnf10* deficiency ameliorates CDAHFD-induced MASH in mice, related to Figure 2. (**A**) Schematic of the feeding protocol for *Rnf10*-HKO mice, with data shown in (B–R). *Rnf10*-Flox mice were used as controls. Mice were fed a CDAHFD for 7 weeks. (**B**) Body weight (n = 6 mice/group). (**C**) Gross liver morphology. Scale bar, 1 cm. (**D**) Liver weight and the LW/BW ratio (n = 6 mice/group). Representative H&E (**E**) and Oil Red O (**F**) staining images, quantitative

results are shown on the right side (n = 6 mice/group). Scale bar, 50 μm. Levels of hepatic TG and FFA (**G**), serum TG and β-OHB (**H**) (n = 6 mice/group). (**I**) Relative protein (n = 3 mice/group) and mRNA (n = 6 mice/group) levels of RNF10 and CPT1A. Representative immunohistochemical staining for F4/80-positive immune cells (**J**), and immunohistochemical staining for MPO-positive immune cells (**L**), quantitative results are shown on the right side (n = 6 mice/group). Scale bar, 50 μm. (**M**) Relative hepatic mRNA expression levels of genes related to pro-inflammatory cytokines in *Rnf10*-HKO compared to *Rnf10*-Flox mice (n = 6 mice/group). Representative images of α-SMA immunohistochemical staining (**N**) and Sirius Red staining (**O**), quantitative results are shown on the right side (n = 6 mice/group). Scale bar, 50 μm. Relative mRNA (**P**) (n = 6 mice/group) and protein (**Q**) (n = 3 mice/group) levels of indicated genes related to liver fibrosis. (**R**) Serum AST and ALT levels (n = 6 mice/group). Data are represented as mean ± SEM. Unpaired 2-tailed Student's *t*-test were performed comparing *Rnf10*-HKO with *Rnf10*-Flox mice. *$P < 0.05$, **$P < 0.01$, and ***$P < 0.001$.

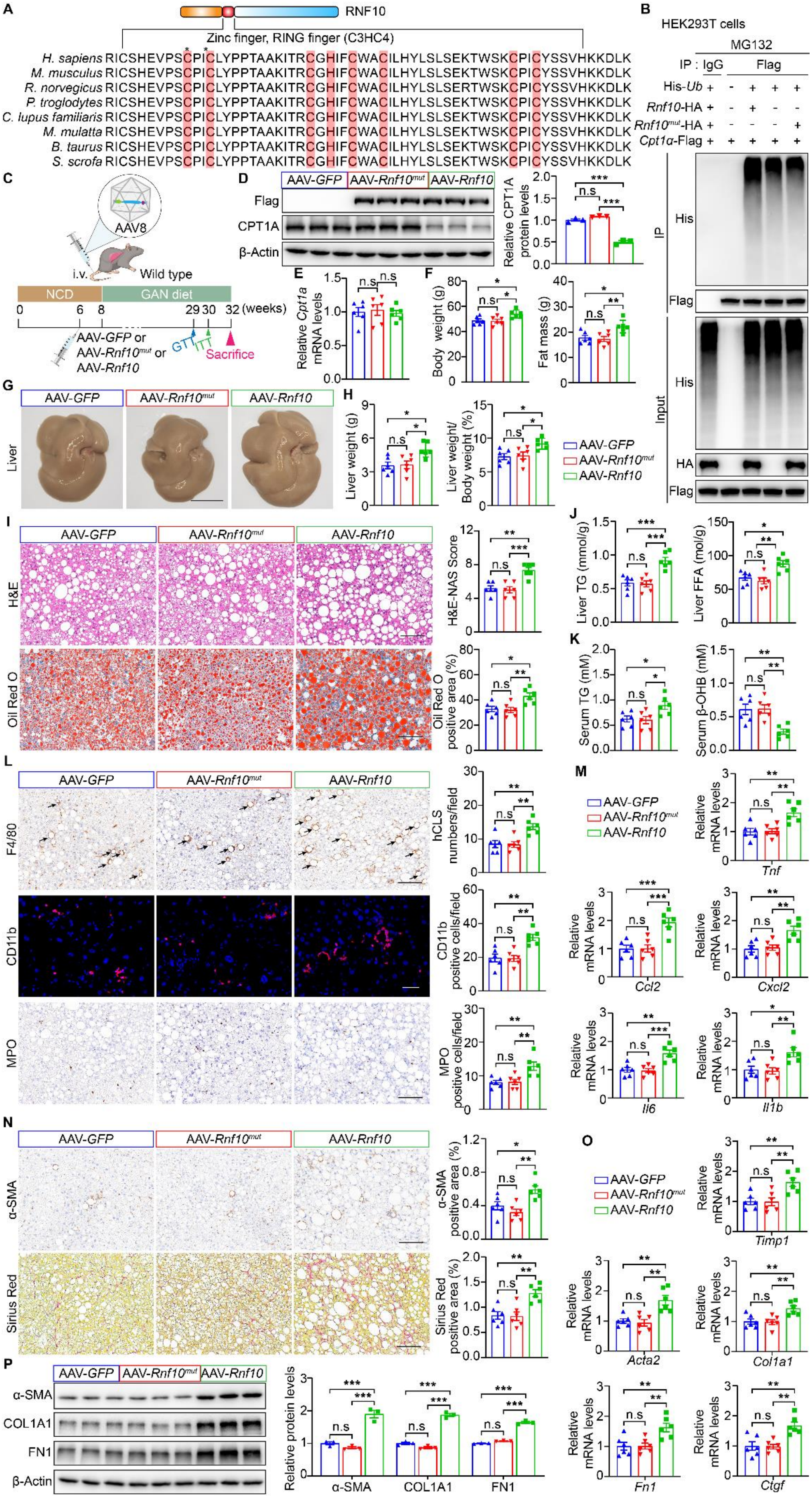
A
RNF10
Zinc finger, RING finger (C3HC4)
H. sapiens RICSHEVPSCPICLYPPTAAKITRCGHIFCWACILHYLSLSEKTWSKCPICYSSVHKKDLK
M. musculus RICSHEVPSCPICLYPPTAAKITRCGHIFCWACILHYLSLSEKTWSKCPICYSSVHKKDLK
R. norvegicus RICSHEVPSCPICLYPPTAAKITRCGHIFCWACILHYLSLSERTWSKCPICYSSVHKKDLK
P. troglodytes RICSHEVPSCPICLYPPTAAKITRCGHIFCWACILHYLSLSEKTWSKCPICYSSVHKKDLK
C. lupus familiaris RICSHEVPSCPICLYPPTAAKITRCGHIFCWACILHYLSLSEKTWSKCPICYSSVHKKDLK
M. mulatta RICSHEVPSCPICLYPPTAAKITRCGHIFCWACILHYLSLSEKTWSKCPICYSSVHKKDLK
B. taurus RICSHEVPSCPICLYPPTAAKITRCGHIFCWACILHYLSLSEKTWSKCPICYSSVHKKDLK
S. scrofa RICSHEVPSCPICLYPPTAAKITRCGHIFCWACILHYLSLSEKTWSKCPICYSSVHKKDLK
B
HEK293T cells
MG132
IP : IgG Flag
His-Ub
Rnf10-HA
Rnf10mut-HA
Cpt1α-Flag
IP
His
Flag
Input
His
HA
Flag
C
AAV8
i.v.
Wild type
NCD
GAN diet
0 6 8 29 30 32 (weeks)
AAV-GFP or AAV-Rnf10mut or AAV-Rnf10
GTT
ITT
Sacrifice
D
AAV-GFP AAV-Rnf10mut AAV-Rnf10
Flag
CPT1A
β-Actin
Relative CPT1A protein levels
E
Relative Cpt1a mRNA levels
F
Body weight (g)
Fat mass (g)
G
AAV-GFP AAV-Rnf10mut AAV-Rnf10
Liver
H
Liver weight (g)
Liver weight/ Body weight (%)
AAV-GFP
AAV-Rnf10mut
AAV-Rnf10
I
H&E
Oil Red O
H&E-NAS Score
Oil Red O positive area (%)
J
Liver TG (mmol/g)
Liver FFA (mol/g)
K
Serum TG (mM)
Serum β-OHB (mM)
L
F4/80
CD11b
MPO
hCLS numbers/field
CD11b positive cells/field
MPO positive cells/field
M
Relative mRNA levels
Tnf
Ccl2
Cxcl2
Il6
Il1b
N
α-SMA
Sirius Red
α-SMA positive area (%)
Sirius Red positive area (%)
O
Timp1
Acta2
Col1a1
Fn1
Ctgf
P
α-SMA
COL1A1
FN1
β-Actin
Relative protein levels
n.s
*
**
***

Supplemental Figure 6. The function of RNF10 in MASLD depends on its E3 ligase activity, related to Figure 4. (**A**) RNF10 domain schematic highlighting C225/228 in the RING domain and their cross-species conservation. (**B**) Ubiquitination of exogenous CPT1A induced by *RNF10* or $RNF10^{mut}$ (C225S/C228S) overexpression in HEK293T cells that were treated with MG132 (20 μM). (**C**) Schematic of the feeding protocol for mice with hepatic-specific overexpression of *Rnf10* (AAV-*Rnf10*) or $Rnf10^{mut}$ (AAV-$Rnf10^{mut}$) for the data shown in (D–P). Relative protein (**D**) (n = 3 mice/group) and mRNA (**E**) (n = 6 mice/group) levels of RNF10 and CPT1A. (**F**) Body weight and fat mass in indicated groups (n = 6 mice/group). (**G**) Gross liver morphology. Scale bar, 1 cm. (**H**) Liver weight and the LW/BW ratio (n = 6 mice/group). (**I**) Representative H&E and Oil Red O staining images, with quantification shown on the right (n = 6 mice/group). Scale bar, 50 μm. Levels of hepatic TG and FFA (**J**), serum TG and β-OHB (**K**) (n = 6 mice/group). (**L**) Representative images of F4/80-positive immune cells by immunohistochemistry, CD11b-positive immune cells by immunofluorescence, and MPO-positive immune cells by immunohistochemistry, with quantification shown on the right (n = 6 mice/group). Scale bar, 50 μm. (**M**) Relative hepatic mRNA expression levels of genes related to pro-inflammatory cytokines (n = 6 mice/group). (**N**) Representative images of α-SMA immunohistochemical and Sirius Red staining, with quantification shown on the right (n = 6 mice/group). Scale bar, 50 μm. Relative mRNA (**O**) (n = 6 mice/group) and protein (**P**) (n = 3 mice/group) levels of indicated genes related to liver fibrosis. Data are represented as mean ± SEM. 1-way ANOVAs were performed in (**D–F** and **H–O**) and 2-way ANOVAs were performed in (**P**). $^{*}P < 0.05$, $^{**}P < 0.01$, and $^{***}P < 0.001$.

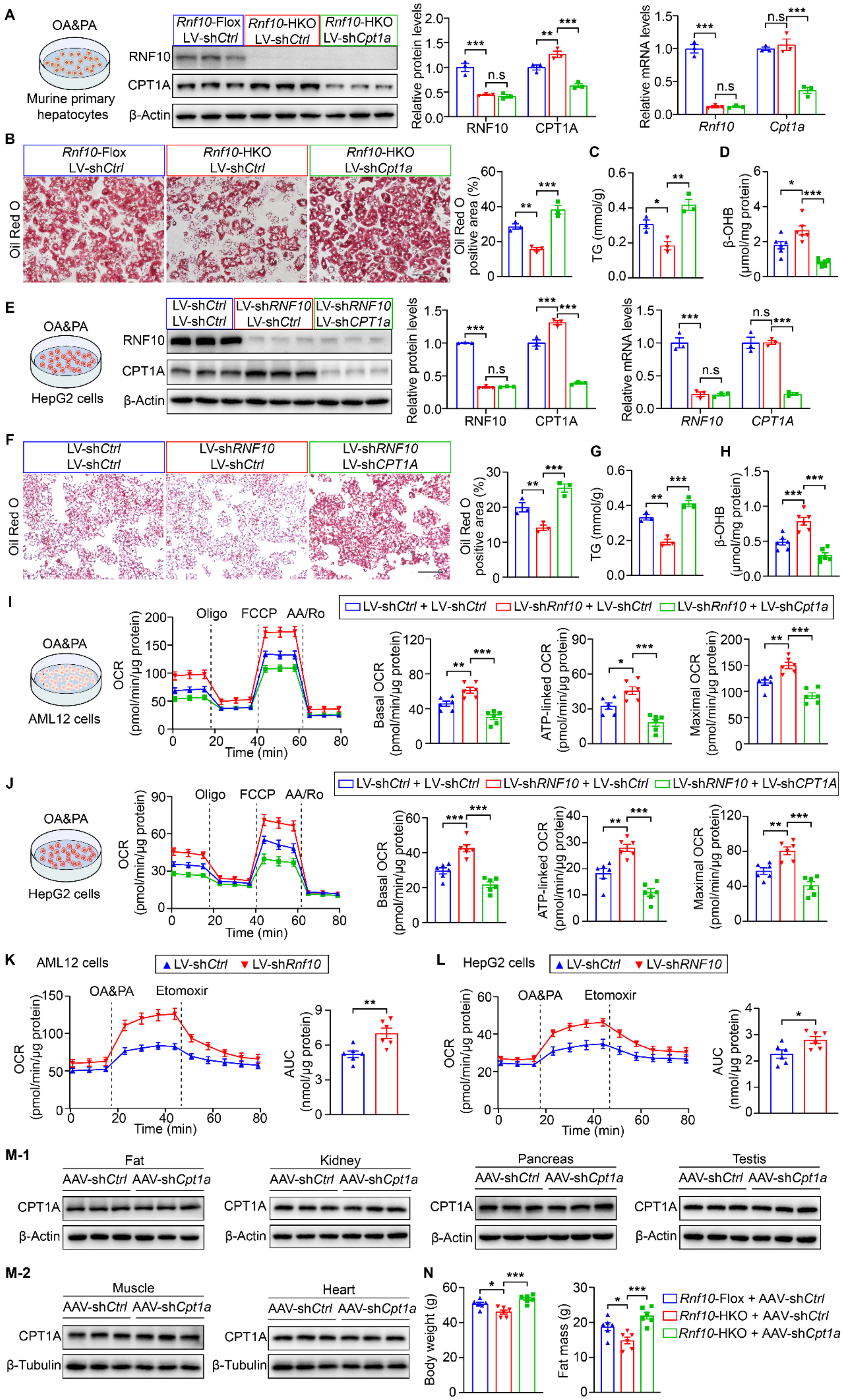

A
OA&PA
Murine primary hepatocytes
Rnf10-Flox LV-shCtrl
Rnf10-HKO LV-shCtrl
Rnf10-HKO LV-shCpt1a
RNF10
CPT1A
β-Actin
Relative protein levels
Relative mRNA levels
Rnf10
Cpt1a
n.s
B
Oil Red O
Oil Red O positive area (%)
C
TG (mmol/g)
D
β-OHB (μmol/mg protein)
E
HepG2 cells
LV-shCtrl LV-shCtrl
LV-shRNF10 LV-shCtrl
LV-shRNF10 LV-shCPT1a
LV-shRNF10 LV-shCPT1A
RNF10
CPT1A
F
G
H
I
AML12 cells
Oligo
FCCP
AA/Ro
OCR (pmol/min/μg protein)
Time (min)
LV-shCtrl + LV-shCtrl
LV-shRnf10 + LV-shCtrl
LV-shRnf10 + LV-shCpt1a
Basal OCR (pmol/min/μg protein)
ATP-linked OCR (pmol/min/μg protein)
Maximal OCR (pmol/min/μg protein)
J
LV-shRNF10 + LV-shCtrl
LV-shRNF10 + LV-shCPT1A
K
LV-shRnf10
Etomoxir
AUC (nmol/μg protein)
L
LV-shRNF10
M-1
Fat
Kidney
Pancreas
Testis
AAV-shCtrl
AAV-shCpt1a
M-2
Muscle
Heart
β-Tubulin
N
Body weight (g)
Fat mass (g)
Rnf10-Flox + AAV-shCtrl
Rnf10-HKO + AAV-shCtrl
Rnf10-HKO + AAV-shCpt1a

Supplemental Figure 7. RNF10 requires CPT1A to modulate fatty acid β-oxidation and lipid accumulation in hepatocytes, related to Figure 5. (**A**) Relative protein and mRNA levels of RNF10 and CPT1A in primary hepatocytes of the indicated groups (n = 3 independent experiments). Representative Oil Red O staining images (**B**) (n = 3 independent experiments) and intracellular TG contents (**C**) (n = 3 independent experiments) in primary hepatocytes of the indicated groups. Scale bar, 50 μm. (**D**) β-OHB levels in the culture medium of primary hepatocytes from the indicated groups (n = 6 independent experiments). (**E**) Relative protein and mRNA levels of RNF10 and CPT1A in HepG2 cells of the indicated groups (n = 3 independent experiments). Representative Oil Red O staining images (**F**) and intracellular TG contents (**G**) (n = 3 independent experiments) in HepG2 cells of the indicated groups. Scale bar, 50 μm. (**H**) β-OHB levels in the culture medium of HepG2 cells from the indicated groups (n = 6 independent experiments). Oxygen consumption rate (OCR) measured in AML12 cells (**I** and **K**) and HepG2 cells (**J** and **L**) of the indicated groups using the XF24 Analyzer (n = 6 independent experiments). (**M**) Relative protein levels of CPT1A in different tissues (fat, kidney, pancreas, testis, heart, and muscle) after AAV-sh*Cpt1a* treatment (n = 3 mice/group). (**N**) Body weight and fat mass in the indicated groups (n = 6 mice/group). Data are represented as mean ± SEM. Unpaired 2-tailed Student's *t*-test were performed in (**K** and **L**), 1-way ANOVAs were performed in (**B–D**, **F–J**, and **N**), and 2-way ANOVAs were performed in (**A** and **E**). $^{*}P < 0.05$, $^{**}P < 0.01$, and $^{***}P < 0.001$.

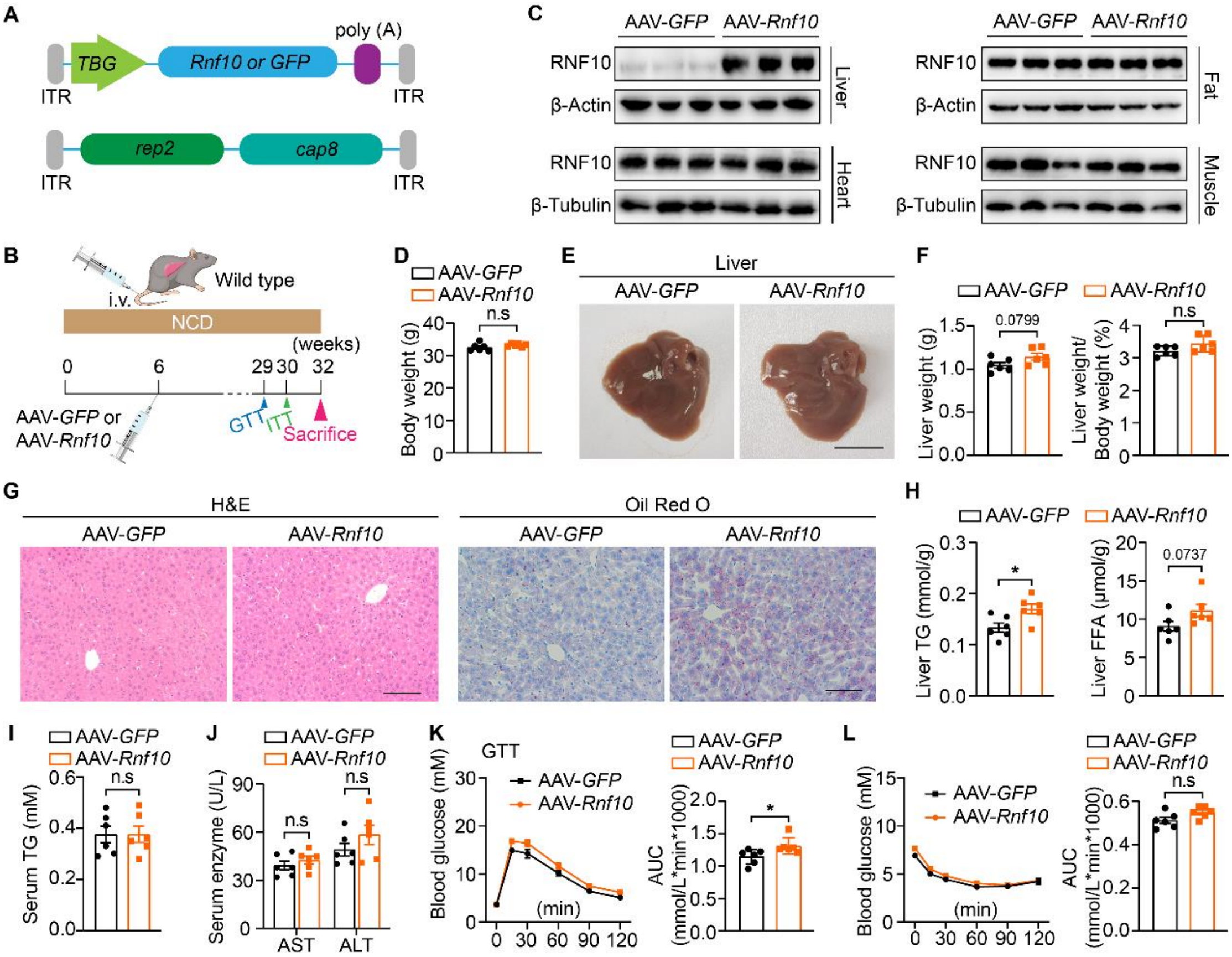

Supplemental Figure 8. Generation and characterization of a murine model with hepatocyte-specific *Rnf10* overexpression using adeno-associated virus 8 (AAV8), related to Figure 6. (**A**) Schematic representation of AAV8 vector expression cassette. (**B**) Schematic showing NCD feeding regimen of AAV-*Rnf10* and AAV-*GFP* mice for the data shown in (C–L). AAV-*GFP* was used as the control. Two weeks following AAV-*Rnf10* or AAV-*GFP* virus injection, mice were fed a NCD for 24 weeks. (**C**) Relative protein levels of RNF10 in different tissues (liver, fat, heart, and muscle) (n = 3 mice/group). (**D**) Body weight in indicated groups (n = 6 mice/group). (**E**) Gross liver morphology. Scale bar, 1 cm. (**F**) Liver weight and the LW/BW ratio (n = 6 mice/group). (**G**) Representative H&E and Oil Red O staining images of liver sections, with quantification shown on the right (n = 6 mice/group). Scale bar, 50 µm. Hepatic TG and FFA contents (**H**), serum TG, AST, and ALT concentrations (**I** and **J**) (n = 6 mice/group). (**K** and **L**) GTT and ITT with the corresponding AUC (n = 6 mice/group). Data are represented as mean ± SEM. Unpaired 2-tailed Student's *t*-test were performed to compare the AAV-*Rnf10* and AAV-*GFP* groups. *$P < 0.05$.

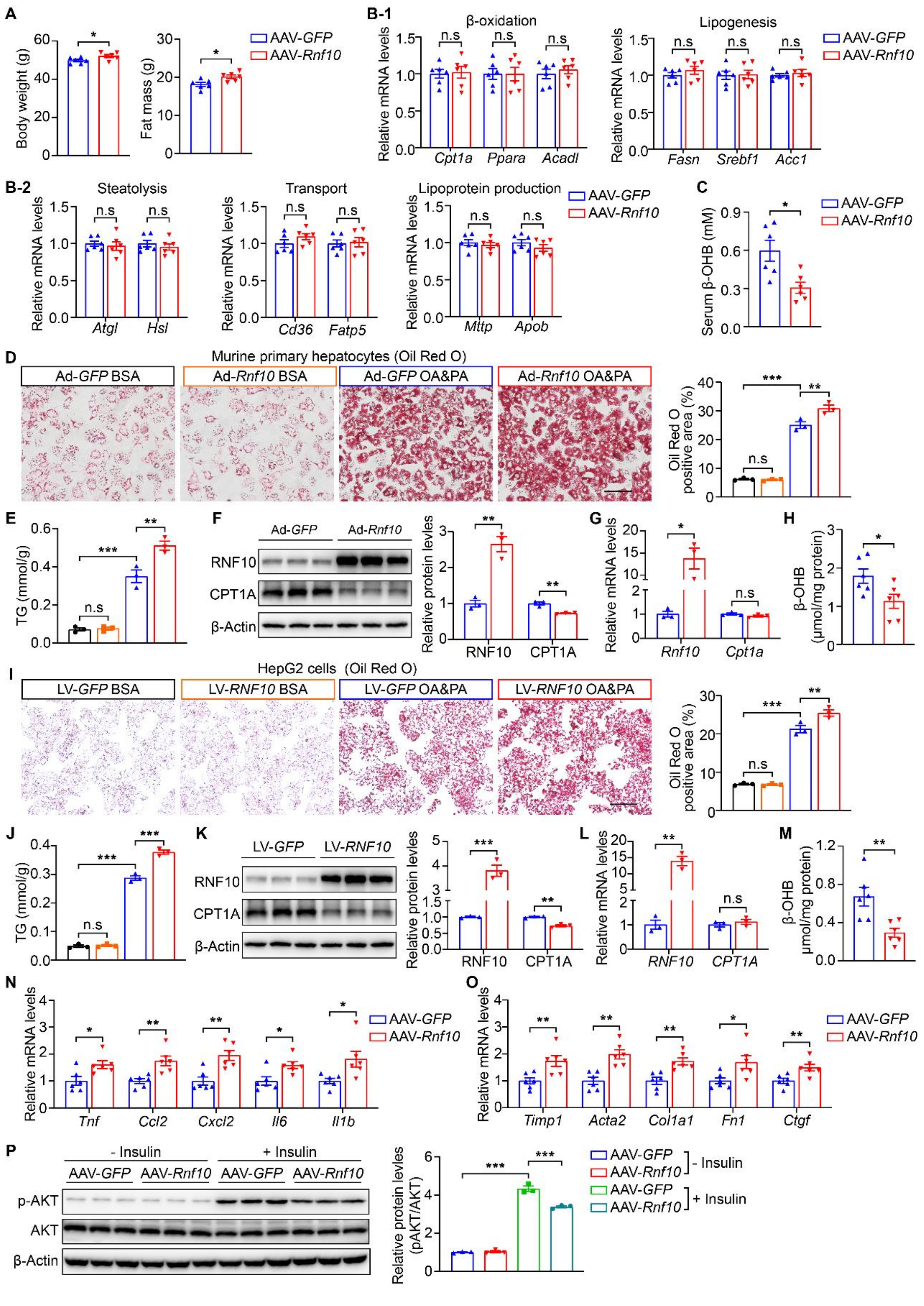

Supplemental Figure 9. ***RNF10*** **upregulation reduced CPT1A protein and β-OHB levels without affecting** ***CPT1A*** **mRNA expression, related to Figure 6.** (**A**) Body weight and fat mass of AAV-*GFP* and AAV-*Rnf10* mice (n = 6 mice/group). (**B**) Relative mRNA levels of lipid metabolism markers in the liver (β-oxidation, lipogenesis,

steatolysis, fatty acid transport and lipoprotein production) (n = 6 mice/group). (**C**) Serum β-OHB levels in the indicated groups (n = 6 mice/group). Representative Oil Red O staining images (**D**) and intracellular TG contents (**E**) (n = 3 independent experiments) in the indicated groups in murine primary hepatocytes. Relative protein (**F**) and mRNA levels (**G**) of RNF10 and CPT1A in primary hepatocytes from the indicated groups (n = 3 independent experiments). (**H**) β-OHB levels in the culture medium of murine primary hepatocytes from the indicated groups (n = 6 independent experiments). Representative Oil Red O staining images (**I**) and intracellular TG contents (**J**) (n = 3 independent experiments) in the indicated groups in HepG2 cells. Relative protein (**K**) and mRNA levels (**L**) of RNF10 and CPT1A in HepG2 cells from the indicated groups (n = 3 independent experiments). (**M**) β-OHB levels in the culture medium of HepG2 cells from the indicated groups (n = 6 independent experiments). (**N**) Relative hepatic mRNA expression levels of genes related to pro-inflammatory cytokines (n = 6 mice/group). (**O**) Relative mRNA levels of indicated genes related to liver fibrosis (n = 6 mice/group). (**P**) Relative protein levels of phosphorylated AKT in liver tissues from the indicated groups (n = 3 mice/group). Data are presented as mean ± SEM. Unpaired 2-tailed Student's *t*-test were performed in (**A–C**, **F–H**, and **K–O**) and 1-way ANOVAs were performed in (**D**, **E**, **I**, **J**, and **P**). *$P < 0.05$, **$P < 0.01$, and ***$P < 0.001$.

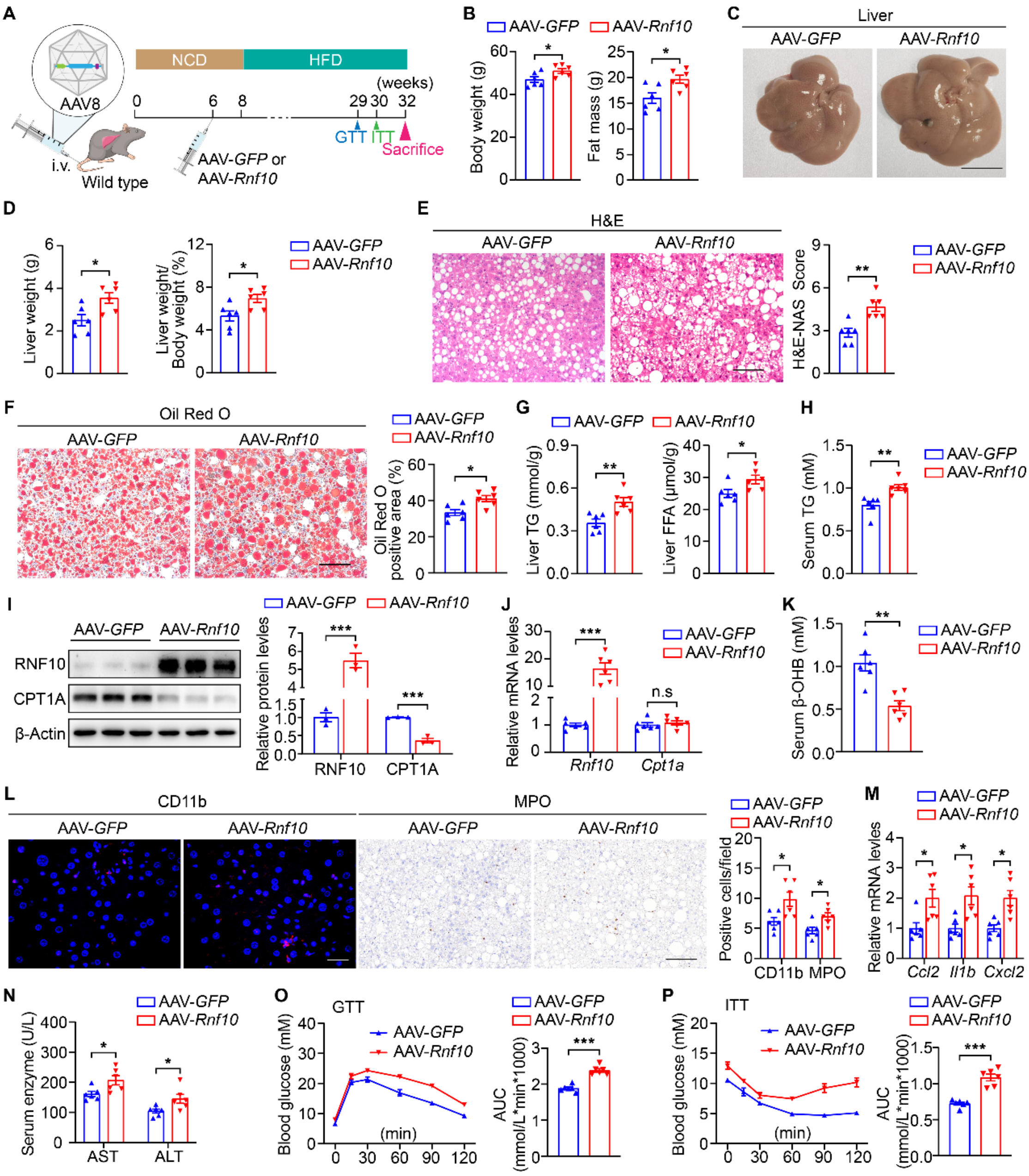

Supplemental Figure 10. Hepatic-specific *Rnf10* overexpression by AAV-*Rnf10* exacerbates HFD-induced liver lipid accumulation and inflammation, related to Figure 6. (**A**) Schematic of HFD-fed AAV-*Rnf10* and AAV-*GFP* mice for the data shown in (B–P). Two weeks following AAV-*Rnf10* or AAV-*GFP* virus injection, mice were fed an HFD for 24 weeks. (**B**) Body weight and fat mass (n = 6 mice/group). (**C**) Gross liver morphology. Scale bar, 1 cm. (**D**) Liver weight and the LW/BW ratio (n = 6 mice/group). Representative H&E (**E**) and Oil Red O (**F**) staining images, with quantification shown on the right (n = 6 mice/group). Scale bar, 50 μm. Levels of hepatic TG and FFA (**G**), serum TG (**H**) (n = 6 mice/group). Relative protein (**I**) (n = 3

mice/group) and mRNA (**J**) (n = 6 mice/group) levels of RNF10 and CPT1A, and serum β-OHB levels (n = 6 mice/group) from AAV-*GFP* and AAV-*Rnf10* mice (**K**) fed an HFD for 24 weeks. Representative images and quantification of CD11b immunofluorescent staining and MPO immunohistochemical staining (**L**), and relative mRNA levels of proinflammatory genes (**M**) (n = 6 mice/group). Scale bar, 50 µm. (**N**) Serum AST and ALT levels (n = 6 mice/group). (**O** and **P**) GTT and ITT with the corresponding AUC (n = 6 mice/group). Data are represented as mean ± SEM. Unpaired 2-tailed Student's *t*-test were performed to compare the AAV-*Rnf10* and AAV-*GFP* groups. $^{*}P < 0.05$, $^{**}P < 0.01$, and $^{***}P < 0.001$.

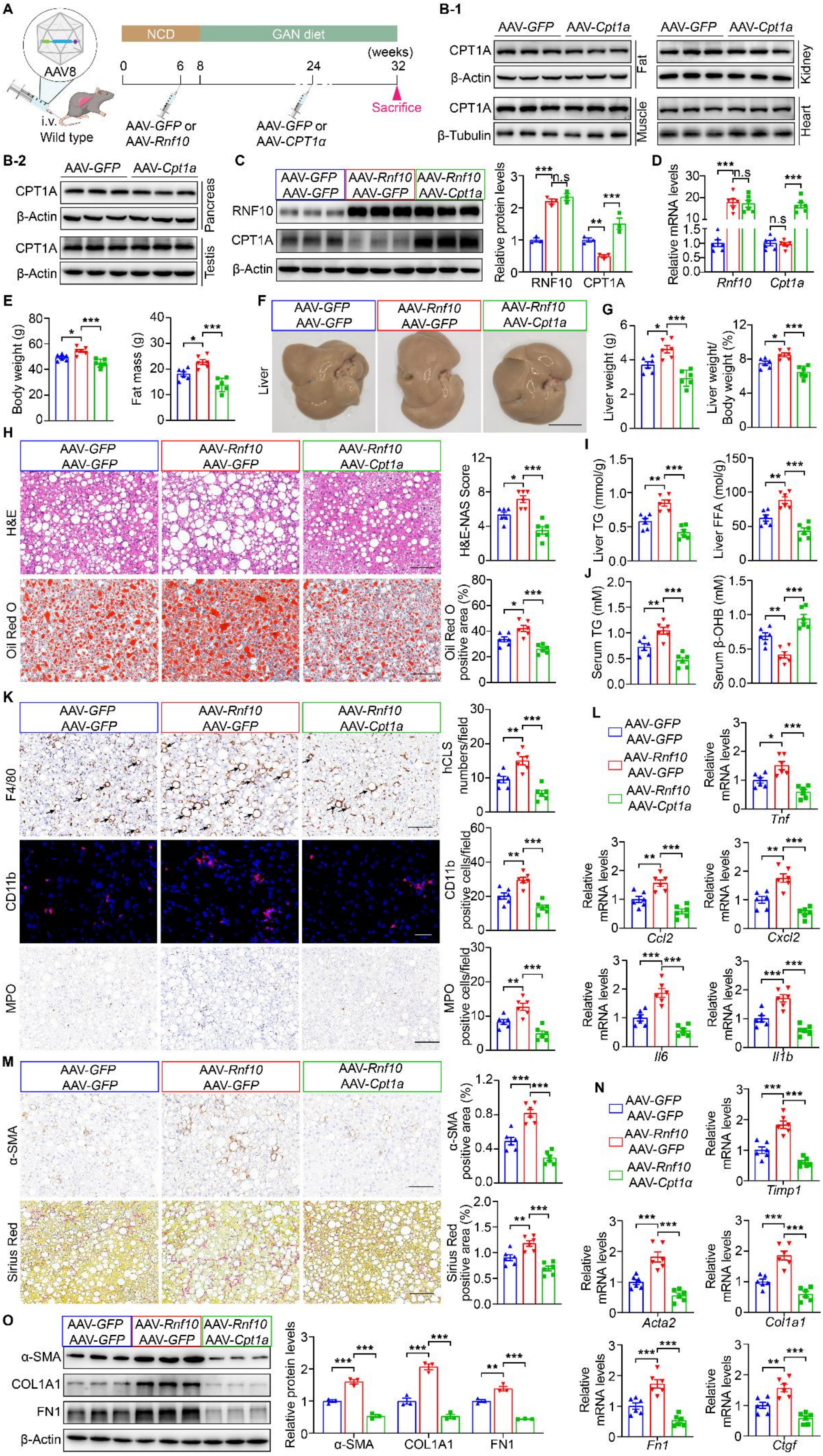
A
AAV8
i.v.
Wild type
NCD
GAN diet
(weeks)
0 6 8 24 32
AAV-GFP or
AAV-Rnf10
AAV-GFP or
AAV-CPT1α
Sacrifice
B-1
AAV-GFP AAV-Cpt1a
CPT1A
β-Actin
β-Tubulin
Fat
Kidney
Muscle
Heart
B-2
Pancreas
Testis
C
AAV-GFP AAV-GFP
AAV-Rnf10 AAV-GFP
AAV-Rnf10 AAV-Cpt1a
RNF10
CPT1A
β-Actin
Relative protein levels
n.s
D
Relative mRNA levels
Rnf10
Cpt1a
E
Body weight (g)
Fat mass (g)
F
Liver
G
Liver weight (g)
Liver weight/ Body weight (%)
H
H&E
Oil Red O
H&E-NAS Score
Oil Red O positive area (%)
I
Liver TG (mmol/g)
Liver FFA (mol/g)
J
Serum TG (mM)
Serum β-OHB (mM)
K
F4/80
CD11b
MPO
hCLS numbers/field
CD11b positive cells/field
MPO positive cells/field
L
Relative mRNA levels
Tnf
Ccl2
Cxcl2
Il6
Il1b
M
α-SMA
Sirius Red
α-SMA positive area (%)
Sirius Red positive area (%)
N
AAV-Rnf10 AAV-Cpt1α
Timp1
Acta2
Col1a1
Fn1
Ctgf
O
α-SMA
COL1A1
FN1
β-Actin
Relative protein levels

Supplemental Figure 11. Overexpression of *Cpt1a* blunts the effects of hepatic-specific *Rnf10* overexpression in MASH, related to Figure 6. (**A**) Experimental scheme to assess whether CPT1A mediates the effects of *Rnf10* overexpression in GAN diet-induced MASH. After 16 weeks of GAN diet feeding, AAV-*GFP* mice injected with AAV-*GFP* and AAV-*Rnf10* mice injected with either AAV-*GFP* or AAV-*Cpt1a* continued a GAN diet for an additional 8 weeks, data are shown in (B–O). (**B**) Relative protein levels of RNF10 in different tissues (n = 3 mice/group). Relative protein (**C**) (n = 3 mice/group) and mRNA (**D**) (n = 6 mice/group) levels of RNF10 and CPT1A. (**E**) Body weight (n = 6 mice/group). (**F**) Gross liver morphology. Scale bar, 1 cm. (**G**) Liver weight and the LW/BW ratio (n = 6 mice/group). (**H**) Representative H&E and Oil Red O staining images, with quantification shown on the right (n = 6 mice/group). Scale bar, 50 μm. Liver TG and FFA contents (**I**), serum TG and β-OHB levels (**J**) (n = 6 mice/group). (**K**) Representative images of F4/80-positive immune cells by immunohistochemistry, CD11b-positive immune cells by immunofluorescence, and MPO-positive immune cells by immunohistochemistry, with quantification shown on the right (n = 6 mice/group). Scale bar, 50 μm. (**L**) Relative hepatic mRNA expression levels of genes related to pro-inflammatory cytokines (n = 6 mice/group). (**M**) Representative images of α-SMA immunohistochemical and Sirius Red staining, with quantification shown on the right (n = 6 mice/group). Scale bar, 50 μm. Relative mRNA (**N**) (n = 6 mice/group) and protein (**O**) (n = 3 mice/group) levels of indicated genes related to liver fibrosis. Data are represented as mean ± SEM. 1-way ANOVAs were performed in (**E** and **G-N**) and 2-way ANOVAs were performed in (**C**, **D**, and **O**). $^*P < 0.05$, $^{**}P < 0.01$, and $^{***}P < 0.001$.

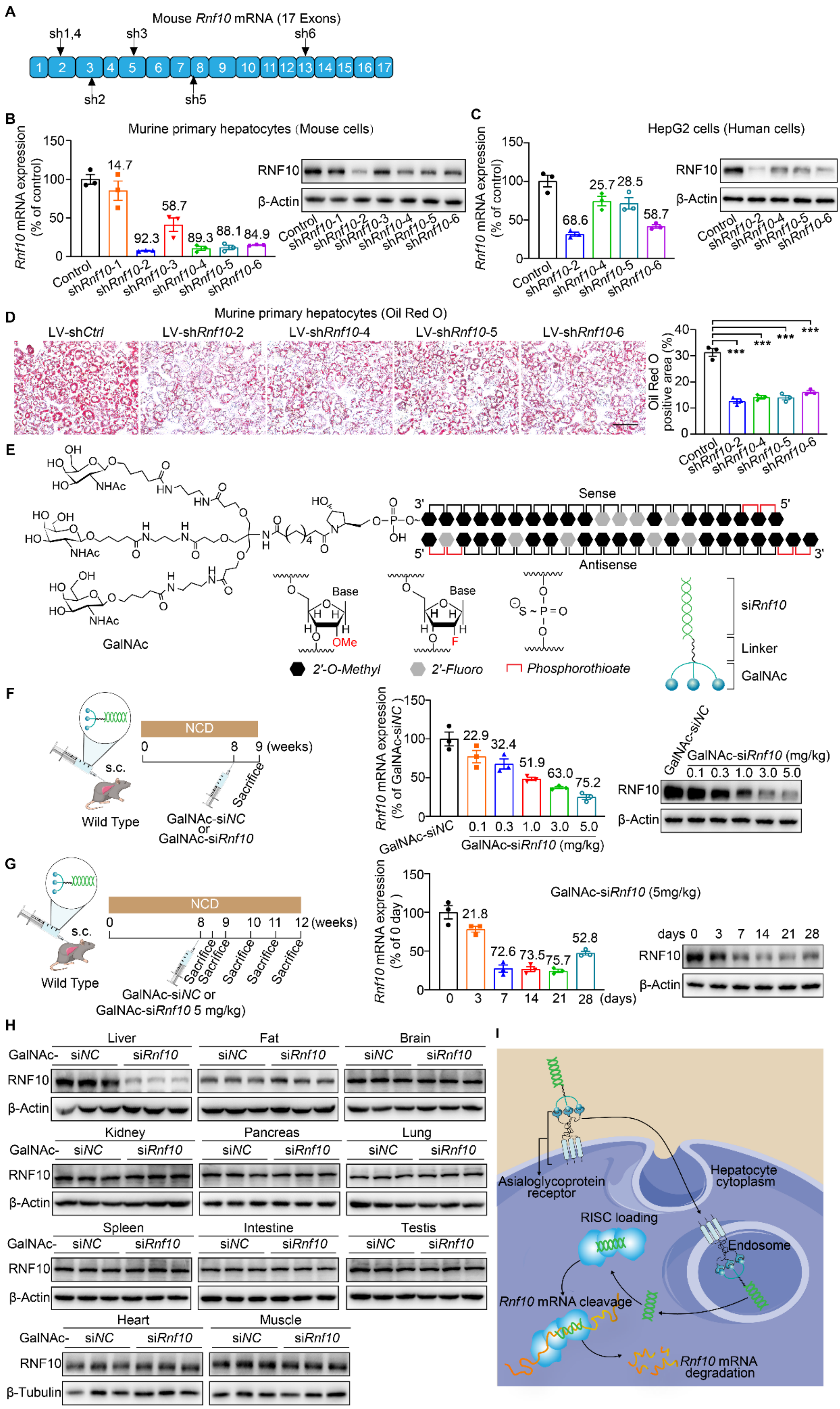

A
Mouse Rnf10 mRNA (17 Exons)
sh1,4
sh3
sh6
sh2
sh5
B
Murine primary hepatocytes (Mouse cells)
Rnf10 mRNA expression (% of control)
RNF10
β-Actin
Control
shRnf10-1
shRnf10-2
shRnf10-3
shRnf10-4
shRnf10-5
shRnf10-6
C
HepG2 cells (Human cells)
D
Murine primary hepatocytes (Oil Red O)
LV-shCtrl
LV-shRnf10-2
LV-shRnf10-4
LV-shRnf10-5
LV-shRnf10-6
Oil Red O positive area (%)
E
Sense
Antisense
GalNAc
Base
2'-O-Methyl
2'-Fluoro
Phosphorothioate
siRnf10
Linker
F
NCD
9 (weeks)
s.c.
Wild Type
Sacrifice
GalNAc-siNC
or
GalNAc-siRnf10
Rnf10 mRNA expression (% of GalNAc-siNC)
GalNAc-siRnf10 (mg/kg)
G
12 (weeks)
GalNAc-siNC or
GalNAc-siRnf10 5 mg/kg)
GalNAc-siRnf10 (5mg/kg)
Rnf10 mRNA expression (% of 0 day)
(days)
H
Liver
Fat
Brain
Kidney
Pancreas
Lung
Spleen
Intestine
Testis
Heart
Muscle
GalNAc-
siNC
siRnf10
β-Tubulin
I
Asialoglycoprotein receptor
Hepatocyte cytoplasm
RISC loading
Endosome
Rnf10 mRNA cleavage
Rnf10 mRNA degradation

Supplemental Figure 12. Identification of a potent si*Rnf10* sequence and investigation of the in vivo silencing capability and longevity of GalNAc-si*Rnf10*-2, related to Figure 7. (**A**) Targeted locations on the mouse *Rnf10* transcript by the candidate sh*Rnf10*. Relative mRNA and protein expression levels in murine primary hepatocytes (**B**) and HepG2 cells (**C**) after being infected with different sh*Rnf10* lentiviruses (n = 3 independent experiments). (**D**) Representative Oil Red O staining images (n = 3 independent experiments) in murine primary hepatocytes of the indicated groups. Scale bar, 50 µm. (**E**) Representative cartoon of chemically modified GalNAc-conjugated si*Rnf10* that were used in vivo studies. (**F**) Dose-response relationships of silencing elicited by GalNAc-si*Rnf10*-2. The mRNA levels of *Rnf10* were normalized against the GalNAc-si*NC* group (left) (n = 3 mice/group) and representative protein levels of RNF10 were analyzed by Western blot (right) (n = 3 mice/group). (**G**) Longevity of silencing elicited by GalNAc-si*Rnf10*-2. The mRNA levels of *Rnf10* were normalized against the 0-day group (left) (n = 3 mice/group) and representative protein levels of RNF10 were analyzed by Western blot (right) (n = 3 mice/group). (**H**) RNF10 protein levels in different organs (liver, fat, brain, kidney, pancreas, lung, spleen, intestine, testis, heart, and muscle) from mice treated with GalNAc-si*Rnf10*-2 for 21 days, GalNAc-si*NC* was used as control (n = 3 mice/group). (**I**) Schematic illustrations of the mechanisms underlying GalNAc-si*Rnf10*-2-mediated *Rnf10* mRNA knockdown. Data are represented as mean ± SEM. 1-way ANOVAs were performed in (**D**). $^{***}P < 0.001$.

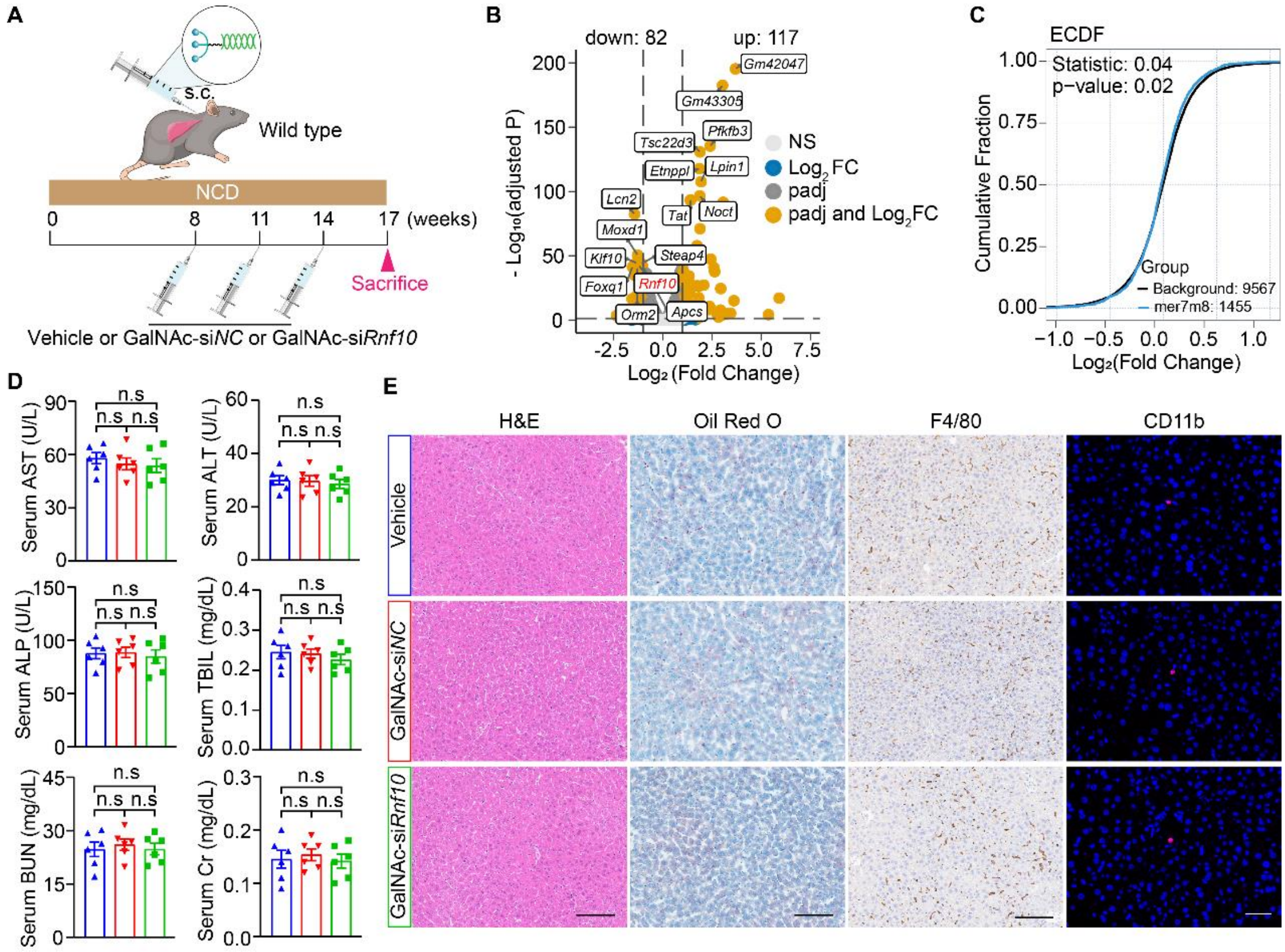

Supplemental Figure 13. Assessment of potential off-target effects and the preliminary safety profile of GalNAc-si*Rnf10*-2, related to Figure 7. (**A**) Experimental design for subcutaneous administration of GalNAc-si*NC* or GalNAc-si*Rnf10*-2 in wild-type mice fed a normal chow diet. (**B**) Volcano plot showing differentially expressed genes identified by RNA-seq (n = 3 mice/group). (**C**) Empirical cumulative distribution function (ECDF) plot showing the distribution of $\log_2$(fold change) values for transcripts containing canonical mer7m8 seed matches versus background transcripts. (**D**) Serum biochemical parameters, including AST, ALT, ALP, TBIL, BUN, and Cr, in mice treated with Vehicle, GalNAc-si*NC*, or GalNAc-si*Rnf10*-2 (n = 6 mice/group). (**E**) Representative liver images stained with H&E, Oil Red O, F4/80, and CD11b from the indicated groups. Scale bar, 50 µm. Data are represented as mean ± SEM. Unpaired 2-tailed Student's *t*-test were performed for GalNAc-si*Rnf10*-2 versus GalNAc-si*NC* group.

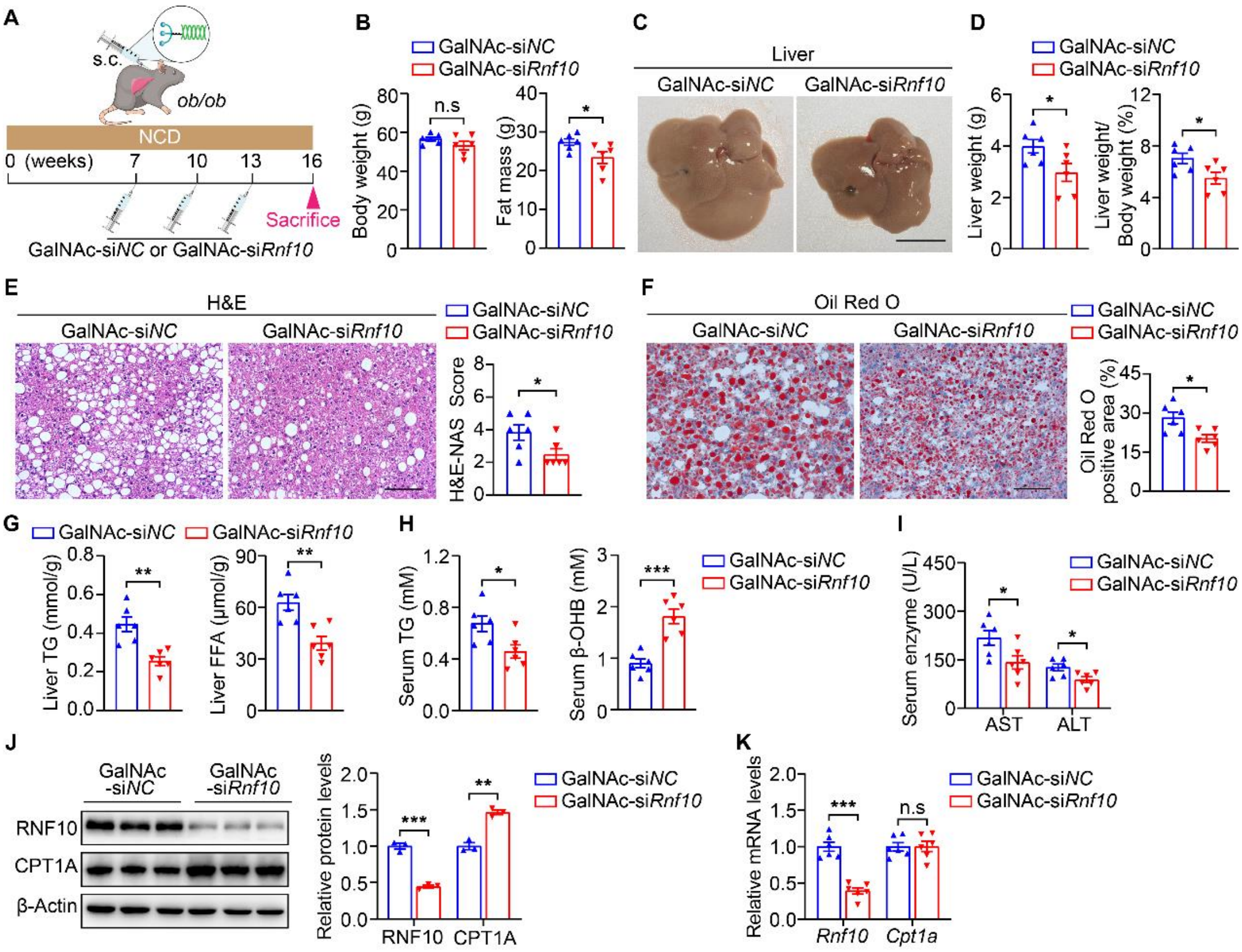

Supplemental Figure 14. GalNAc-si*Rnf10*-2 reduces hepatic steatosis in *ob/ob* mice, related to Figure 7. (**A**) Treatment schedule for *ob/ob* mice, with an overview of GalNAc-si*Rnf10*-2 injection time points. GalNAc-si*Rnf10*-2 (5 mg/kg) was subcutaneously administered to male *ob/ob* mice at 7, 10, and 13 weeks of age. Analyses were performed after 16 weeks of NCD feeding, and data are shown in (B–K). GalNAc-si*NC* was used as the control. (**B**) Body weight and fat mass in the indicated groups (n = 6 mice/group). (**C**) Gross liver morphology. Scale bar, 1 cm. (**D**) Liver weight and the LW/BW ratio (n = 6 mice/group). Representative H&E (**E**) and Oil Red O (**F**) staining images, with quantification shown on the right (n = 6 mice/group). Scale bar, 50 μm. Hepatic TG and FFA contents (**G**), and serum TG, β-OHB, AST, and ALT levels (**H** and **I**) (n = 6 mice/group). Relative protein (**J**) (n = 3 mice/group) and mRNA (**K**) (n = 6 mice/group) levels of RNF10 and CPT1A. Data are represented as mean ± SEM. Unpaired 2-tailed Student's *t*-test were performed for GalNAc-si*Rnf10*-2 versus GalNAc-si*NC* group. *P < 0.05, **P < 0.01, and ***P < 0.001.

Supplemental Table 1. Primers for genotyping transgenic mice.

Primer	Primer Sequence (5'-3')
F1	TGCCTGGGCTTGTTTTTATG
R1	TGGTGCTCTGAGGAAGCGC
F2	TGAGGTATGGAGAGAGAATGGC
R2	ACAACACACCTGCTGTCATGTC
Alb-cre P1	TGCAAACATCACATGCACAC
Alb-cre P2	TTGGCCCCTTACCATAACTG
Alb-cre P3	GAAGCAGAAGCTTAGGAAGATGG

Supplemental Table 2. Sequences for shRNAs.

shRNA	sequence (5'-3')
Human sh*RNF10*-1	CCACTTGTTGAATTTCACTTT
Human sh*RNF10*-2	CCAAGACTACACAGCTCATTT
Human sh*CPT1A*-1	GCCATGAAGCTCTTAGACAAA
Human sh*CPT1A*-2	CGATGTTACGACAGGTGGTTT
Mouse sh*Rnf10*-1	CGTCGCTCCAATTCACAGAAA
Mouse sh*Rnf10*-2	CCCTAAGAAGATCAACCTGAA
Mouse sh*Rnf10*-3	GCAAATGTCCCATCTGCTACA
Mouse sh*Rnf10*-4	ATGGATCCAAGCGCTACAATC
Mouse sh*Rnf10*-5	GGAGGTCAAGATTCGGGAAGA
Mouse sh*Rnf10*-6	GCAGTTTCCGGCCTTCAATTC
Mouse sh*Cpt1a*-1	GCTATGGTGTTTCCTACATTA
Mouse sh*Cpt1a*-2	ATGGACTCTAGTGATACAAAC
sh*Ctrl*	ACCTAAGGTTAAGTCGCCCTCG

Supplemental Table 3. List of antibodies

Antibodies	Source	Cat. no.	Clone no.	Dilution
anti-RNF10	Abclonal	A12296	N/A	WB: 1:1000<br>IP: 2 μg/sample
anti-RNF10	Abclonal	A24502	N/A	IF: 1:200
anti-RNF10	Proteintech	16936-1-AP	N/A	IHC: 1:100
anti-CPT1A	Proteintech	15184-1-AP	N/A	WB: 1:10000<br>IF: 1:200<br>IHC: 1:1000<br>IP: 2 μg/sample
anti-COL1A1	Abclonal	A1352	N/A	WB: 1:1000
anti-FN1	Abclonal	A12932	N/A	WB: 1:1000
anti-α-SMA	Abclonal	A7248	N/A	WB: 1:1000<br>IHC: 1:100
anti-F4/80	Abclonal	A23788	ARC61555	IHC: 1:400
anti-CD11b	Abclonal	A24120	ARC63897	IF: 1:200
anti-MPO	Abclonal	A22900	ARC3177	IHC: 1:5000
anti-PPARα	Abclonal	A6697	N/A	WB: 1:1000
anti-ACADL	Abclonal	A1266	N/A	WB: 1:1000
anti-FASN	Proteintech	10624-2-AP	N/A	WB: 1:1000
anti-SREBF1	Abclonal	A15586	N/A	WB: 1:1000
anti-ACC1	Proteintech	21923-1-AP	N/A	WB: 1:1000
anti-ATGL	Abclonal	A5126	ARC1192	WB: 1:1000
anti-HSL	Abclonal	A15686	N/A	WB: 1:1000
anti-CD36	Abclonal	A5792	N/A	WB: 1:1000
anti-FATP5	Proteintech	25347-1-AP	N/A	WB: 1:1000
anti-MTTP	Abclonal	A1746	N/A	WB: 1:1000
anti-ApoB	Proteintech	20578-1-AP	N/A	WB: 1:1000
anti-ECHS1	Proteintech	11305-1-AP	N/A	WB: 1:1000
anti-AMPKα1	Abclonal	A1229	N/A	WB: 1:1000
anti-CGI58	Proteintech	12201-1-AP	N/A	WB: 1:1000
anti-ACSL1	Proteintech	13989-1-AP	N/A	WB: 1:1000
anti-p-AKT	Cohesion Biosciences	CPA1031	N/A	WB: 1:1000
anti-AKT	Cohesion Biosciences	CPA1034	N/A	WB: 1:1000
anti-HA-tag	MBL	M180-3	TANA2	WB: 1:1000<br>IP: 2 μg/sample
anti-Flag-tag	MBL	M185-3L	FLA-1	WB: 1:1000<br>IP: 2 μg/sample
anti-His-tag	Abclonal	AE003	AMC0149	WB: 1:5000
anti-Myc-tag	Proteintech	60003-2-lg	1A5A2	WB: 1:10000
anti-Ub	Proteintech	10201-2-AP	N/A	WB: 1:1000

anti-GST-tag	Proteintech	10000-0-AP	N/A	WB: 1:1000
anti-β-Tubulin	Immunoway	YM3030	5G3	WB: 1:5000
anti-β-Actin	Abclonal	AC026	ARC5115-01	WB: 1:100000
Rabbit Control IgG	Abclonal	AC005	N/A	IP: 2 μg/sample
Mouse Control IgG	Abclonal	AC011	N/A	IP: 2 μg/sample
HRP Goat anti-Mouse IgG (H+L)	Abclonal	AS003	N/A	WB: 1:5000
HRP Goat anti-Rabbit IgG (H+L)	Abclonal	AS014	N/A	WB: 1:5000

Supplemental Table 4. Primers for qPCR.

Gene		Sequence (5'-3')
Human *RNF10*	F	AATGGTGGAAGACGAGATGAG
	R	ACGGGGTTCAAAAGTGAAATT
Human *CPT1A*	F	TGAGCACGGCAAGATGAGTC
	R	GTACAACATGGGTTTTCGGC
Human *ACTB*	F	AGAGCTACGAGCTGCCTGAC
	R	AGCACTGTGTTGGCGTACAG
Mouse *Rnf10*	F	CCCTTTAGCTCTTCTTCTAATGGTG
	R	AACAAGTGGTTCAGGTTGATCTTCT
Mouse *Cpt1a*	F	TGGGCATGATTGCAAAGATC
	R	AAGAGGACGCCACTCACGAT
Mouse *Ppara*	F	ACAAGGCCTCAGGGTACCA
	R	GCCGAAAGAAGCCCTTACAG
Mouse *Acadl*	F	GCAGTTACTTGGGAAGAGCA
	R	CGTAATTCGCAATATAGGGC
Mouse *Fasn*	F	GAAGTTGCCCGAGTCAGAGAA
	R	CCGCTTGGGTAATCCATAGAG
Mouse *Srebf1*	F	GGAGCCATGGATTGCACATT
	R	CAGGAAGGCTTCCAGAGAGG
Mouse *Acc1*	F	GAACGTGCAATCCGATTTGT
	R	TTGTTGTTGTTGGGTCCTCC
Mouse *Atgl*	F	GTTCAACCTTCGCAATCTCT
	R	CCATCTCTGTAGCCCTGTTT
Mouse *Hsl*	F	CTGAGATTGAGGTGCTGTCG
	R	CCTGGGATCAGAGGTGAGTG
Mouse *Cd36*	F	TGATTAATGGCACAGACGCAG
	R	GGTCGATTTCAGATCCGAACA
Mouse *Fatp5*	F	TCGAGCTACGATTAAGTGGA
	R	GTGATAAGATGGCTGGCTTT
Mouse *Mttp*	F	CTCTTTCCTACTTTGTGTGCCA
	R	AGCTTGTATAGCCGCTCATTAT
Mouse *Apob*	F	AAAGTTCCAGCGGTGTCCAG
	R	CCTCAGGGTTGAAGCCATAC
Mouse *Ccl2*	F	TACAAGAGGATCACCAGCAGC
	R	ACCTTAGGGCAGATGCAGTT
Mouse *Il1b*	F	CCGTGGACCTTCCAGGATGA
	R	GGGAACGTCACACACCAGCA
Mouse *Cxcl2*	F	GCGCCCAGACAGAAGTCATA
	R	CAGTTAGCCTTGCCTTTGTTCA
Mouse *Tnf*	F	CATCTTCTCAAAATTCGAGTGACAA
	R	TGGGAGTAGACAAGGTACAACCC
Mouse *Il6*	F	TAGTCCTTCCTACCCCAATTTCC

	R	TTGGTCCTTAGCCACTCCTTC
Mouse *Timp1*	F	GCAACTCGGACCTGGTCATAA
	R	CGGCCCGTGATGAGAAACT
Mouse *Acta2*	F	GCTGTTTTCCCATCCATCGT
	R	TCCCCCACATAGCTGTCTTT
Mouse *Col1a1*	F	TGCTAACGTGGTTCGTGACCGT
	R	ACATCTTGAGGTCGCGGCATGT
Mouse *Fn1*	F	TACGGAGAGACAGGAGGAAATA
	R	CATACAGGGTGATGGTGTAGTC
Mouse *Ctgf*	F	TCTTCTGCGATTTCGGCTCC
	R	ACTCACCGCTGCGGTACACC
Mouse *β-actin*	F	TTTTCCAGCCTTCCTTCTTG
	R	TGGCATAGAGGTCTTTACGG

References

1. Juluri R, et al. Generalizability of the nonalcoholic steatohepatitis Clinical Research Network histologic scoring system for nonalcoholic fatty liver disease. J Clin Gastroenterol. 2011;45(1):55–58.
2. Wang R, et al. Sterol-regulatory-element-binding protein 1c mediates the effect of insulin on the expression of Cidea in mouse hepatocytes. Biochem J. 2010;430(2):245–254.
3. Tiscornia G, et al. Production and purification of lentiviral vectors. Nat Protoc. 2006;1(1):241–245.
4. Alkhouri N, et al. Development and validation of a new histological score for pediatric non-alcoholic fatty liver disease. J Hepatol. 2012;57(6):1312–1318.
5. Cazares T, et al. SeedMatchR: identify off-target effects mediated by siRNA seed regions in RNA-seq experiments. Bioinformatics. 2024;40(1):btae011.
