# Supplementary material for: Targeting the E3 ubiquitin ligase RNF10 ameliorates metabolic dysfunction–associated steatohepatitis in mice
Source: J Clin Invest. 2026 Aug 6;136(18):e194148. doi: 10.1172/JCI194148 (PMC13574155; doi:10.1172/JCI194148)

Full unedited gel for Figure 1

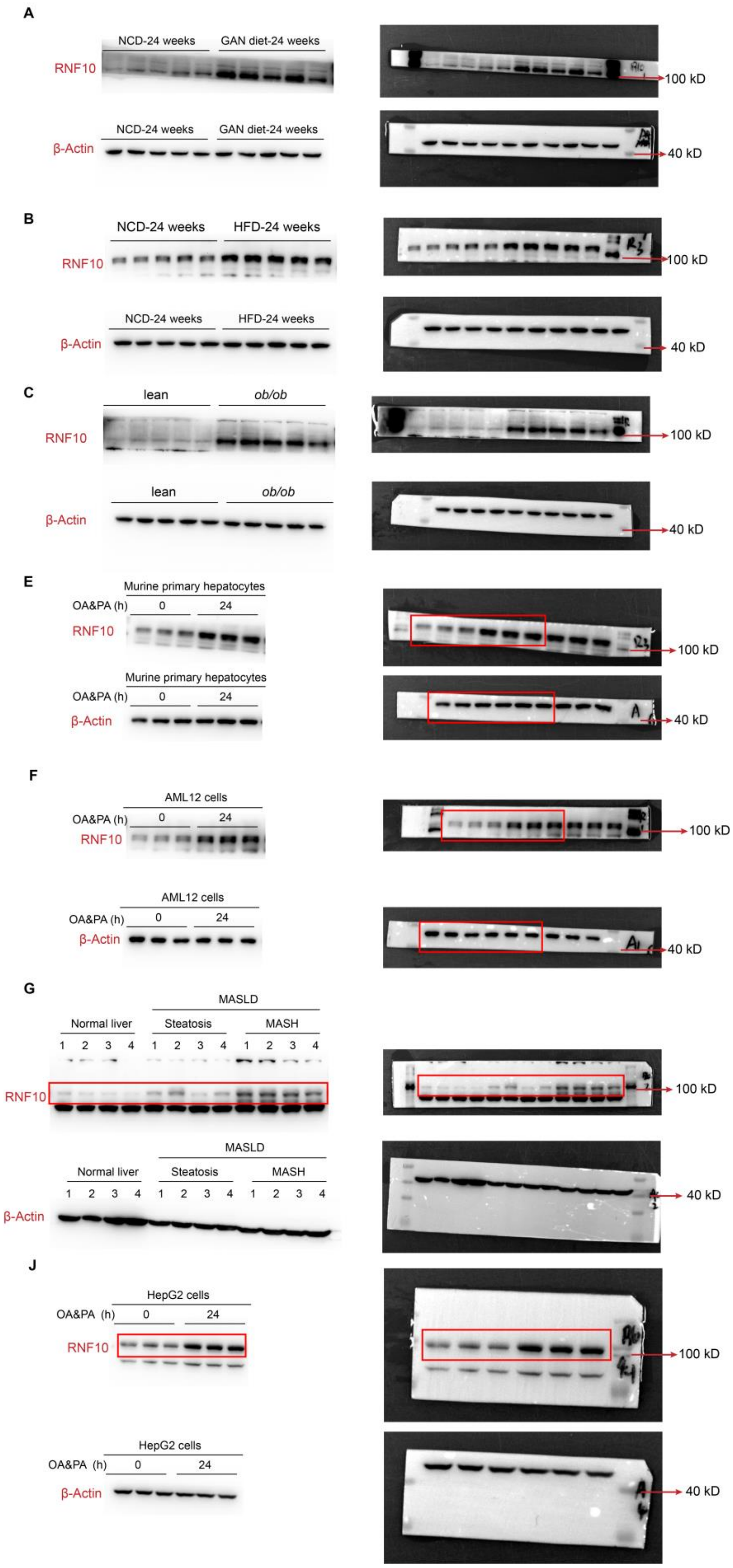

Full unedited gel for Figure 2

H

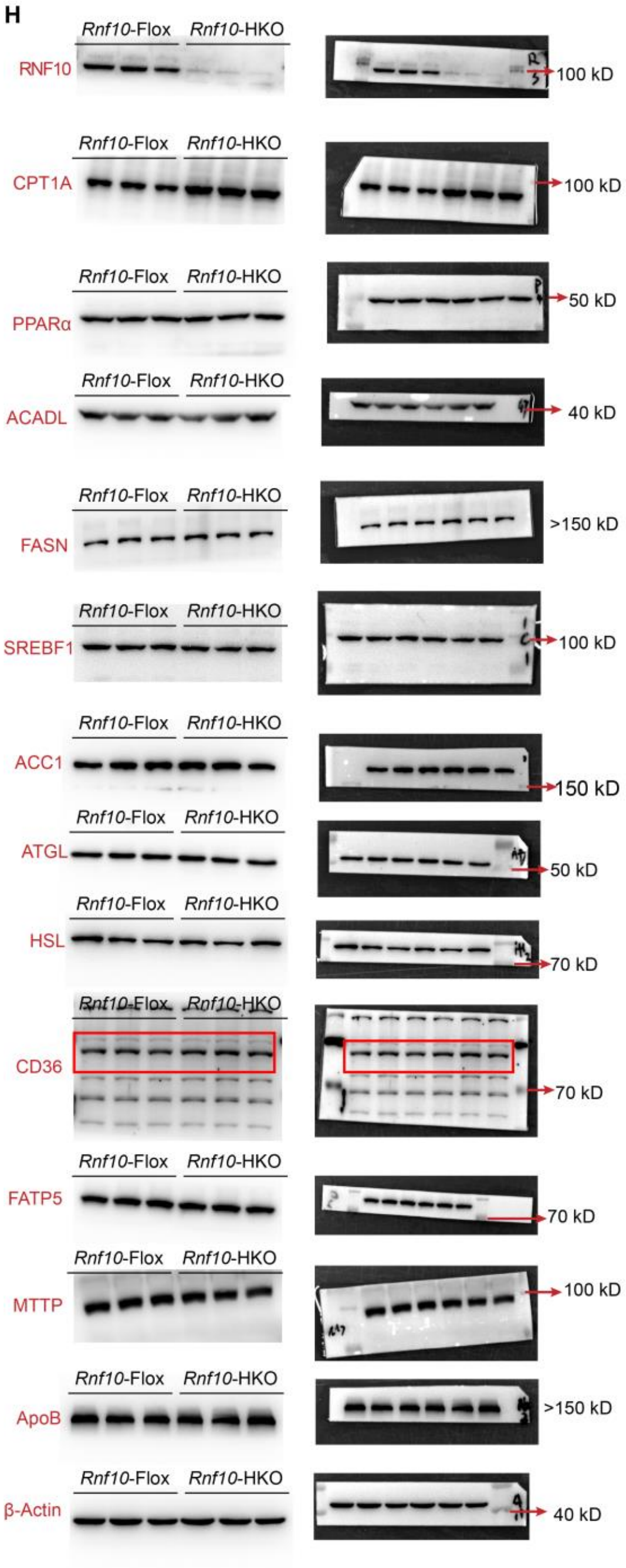

N

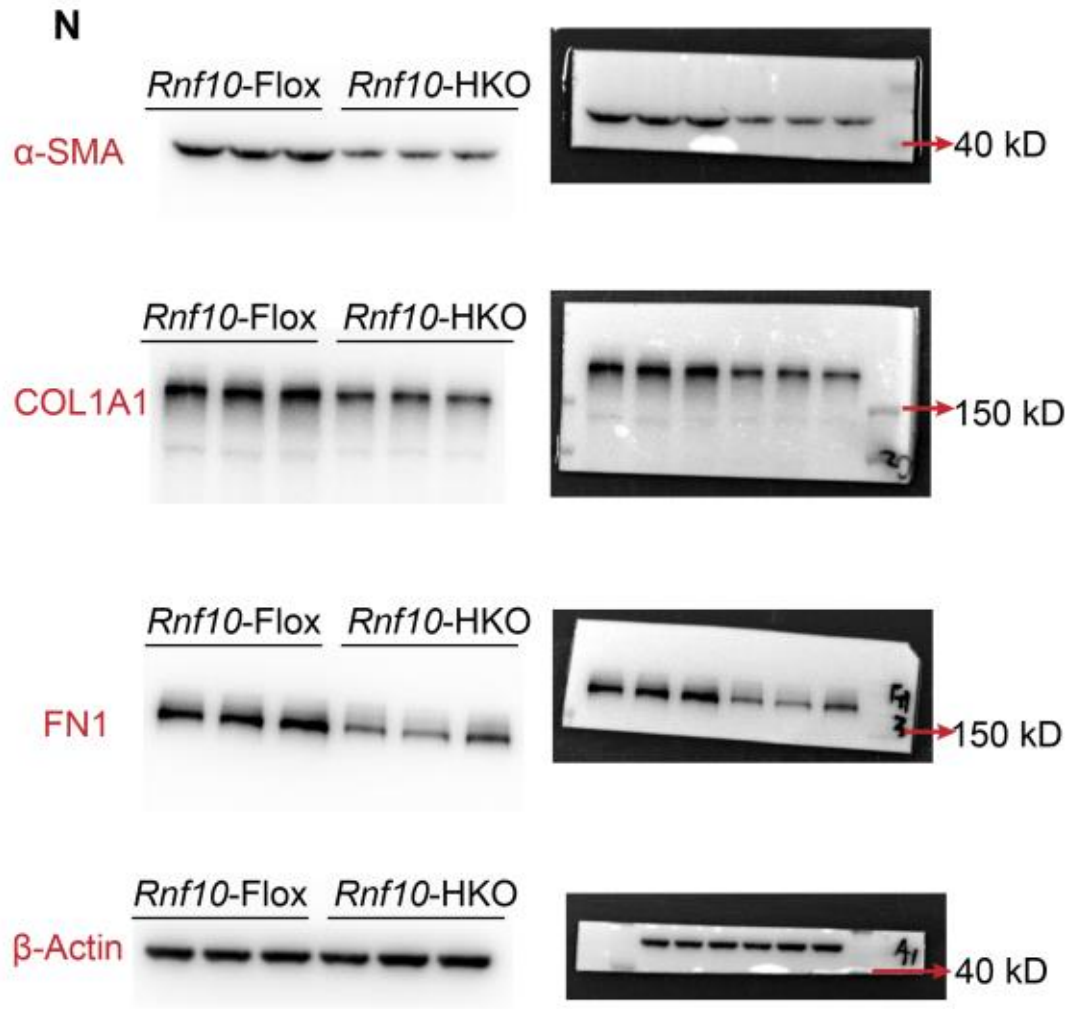

Full unedited gel for Figure 3

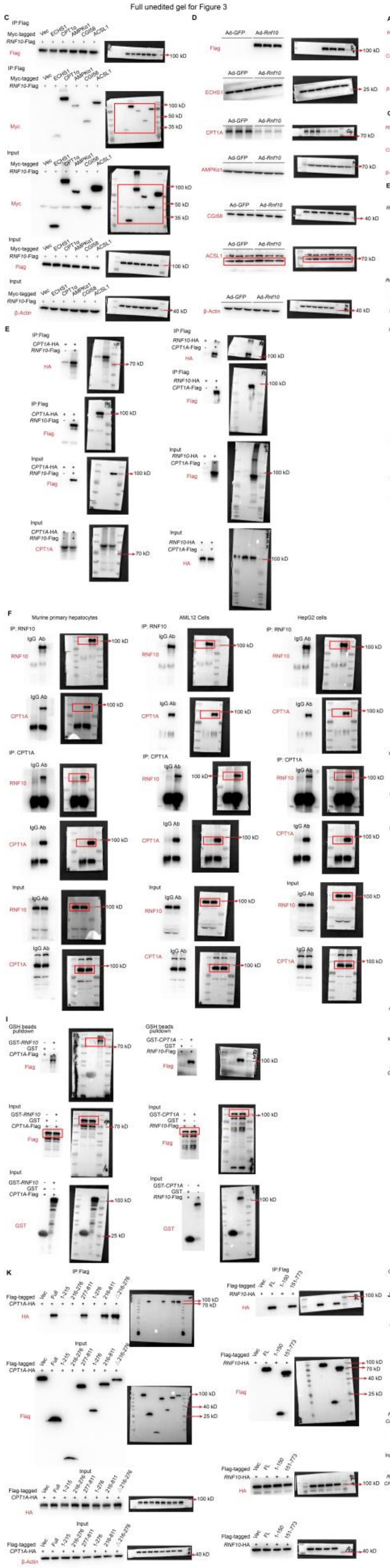

Full unedited gel for Figure 4

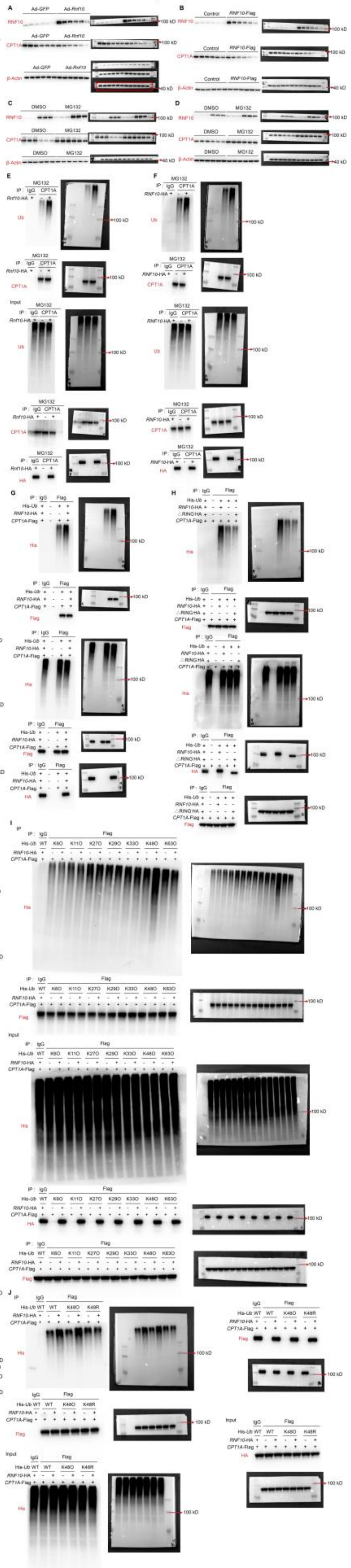

Full unedited gel for Figure 6

B

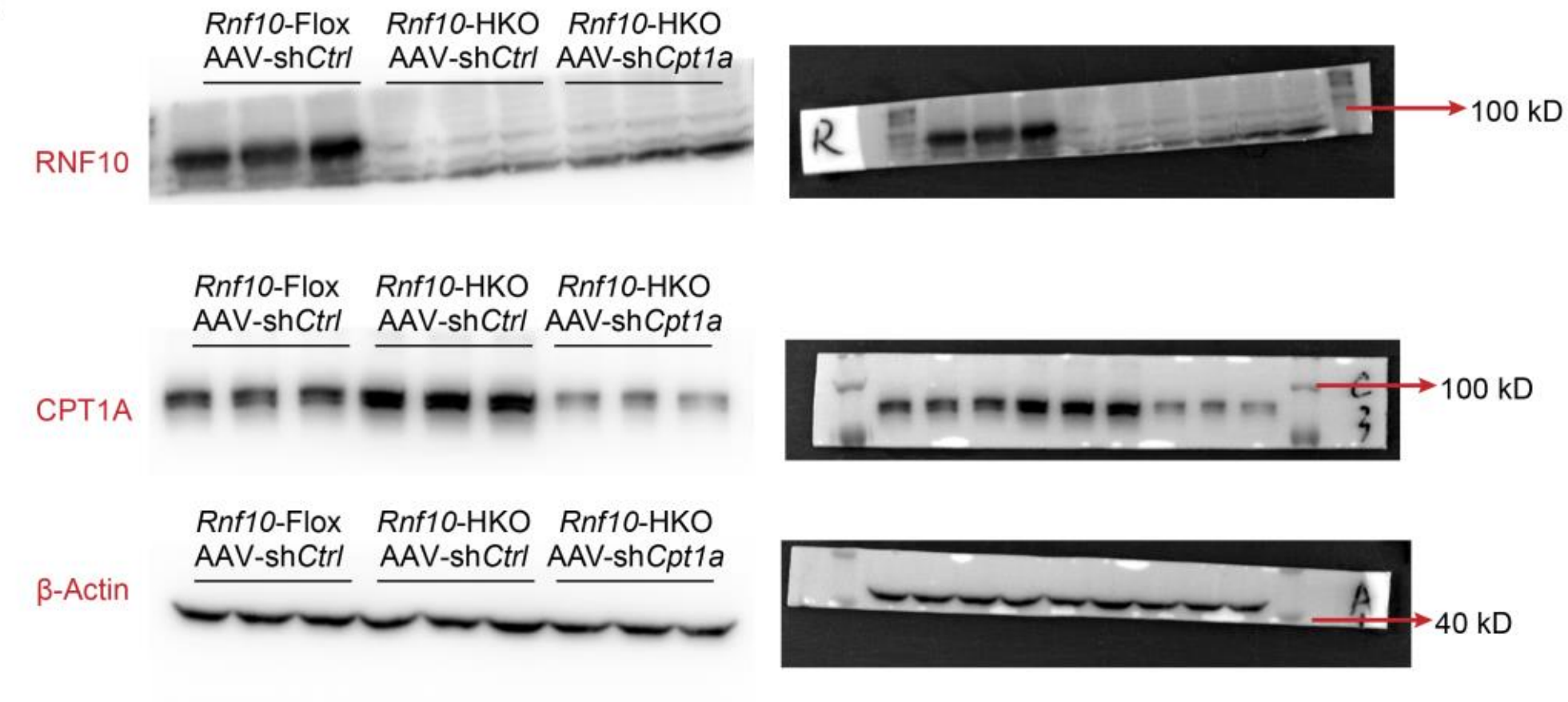

N

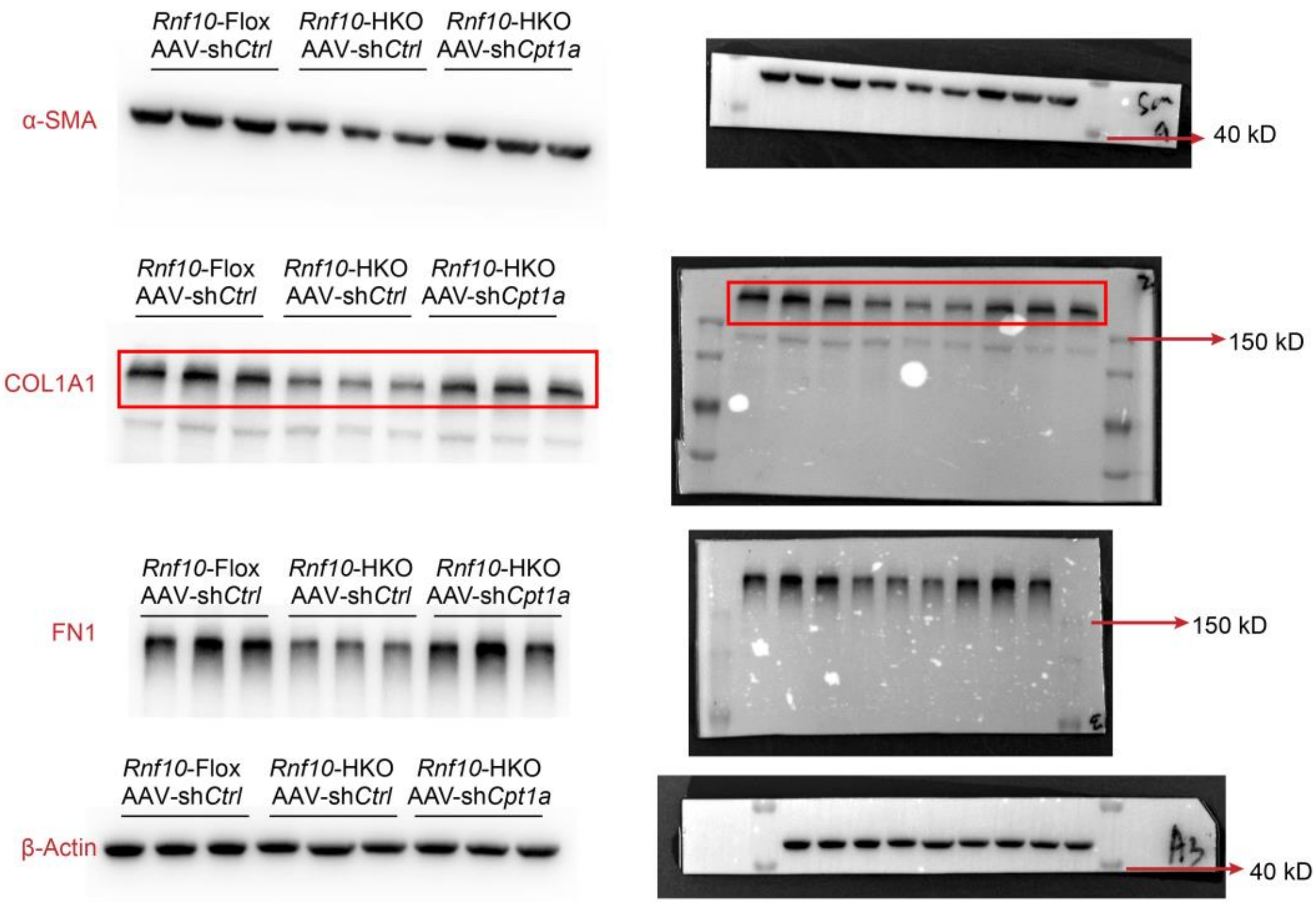

Full unedited gel for Figure 6

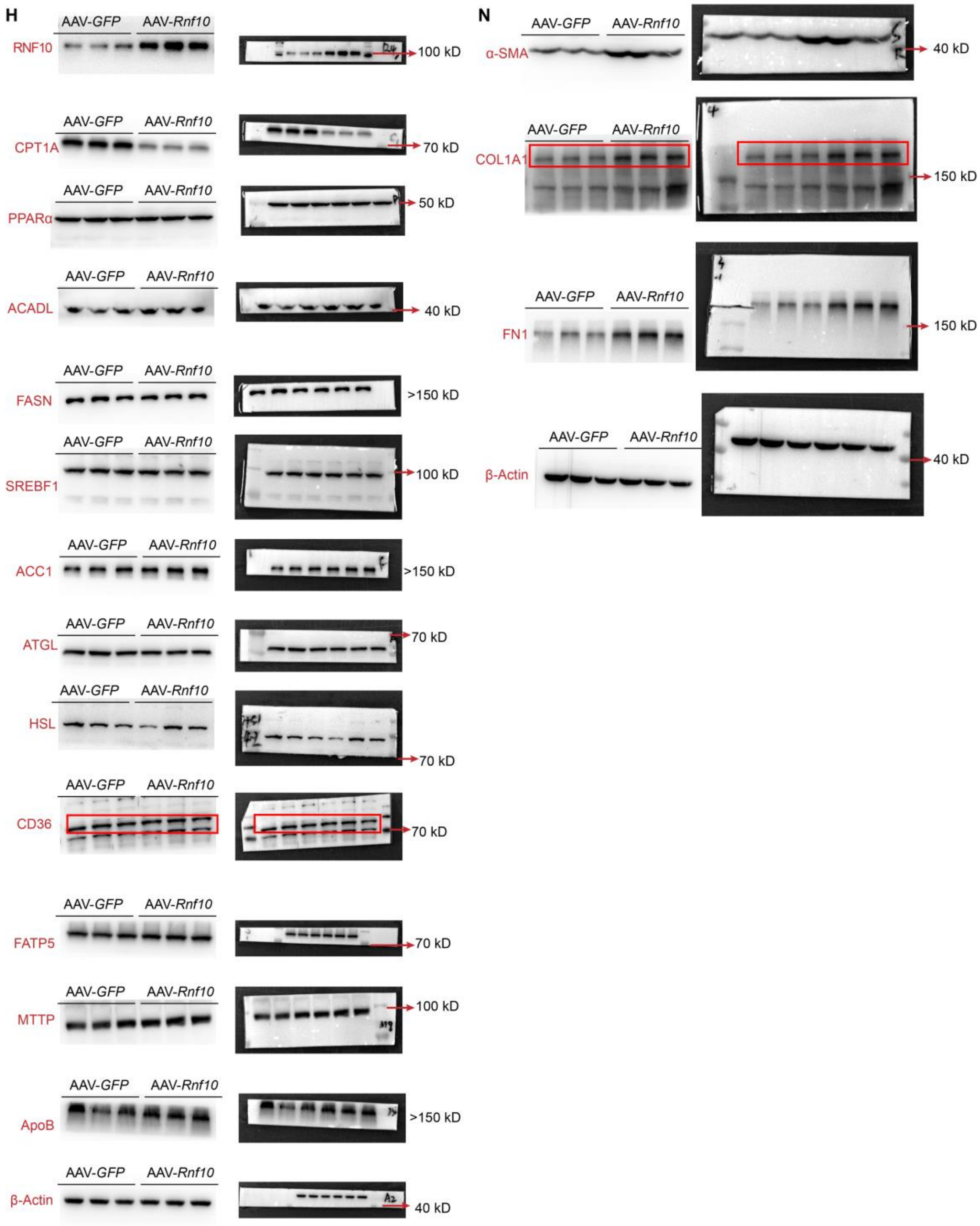

Full unedited gel for Figure 7

B

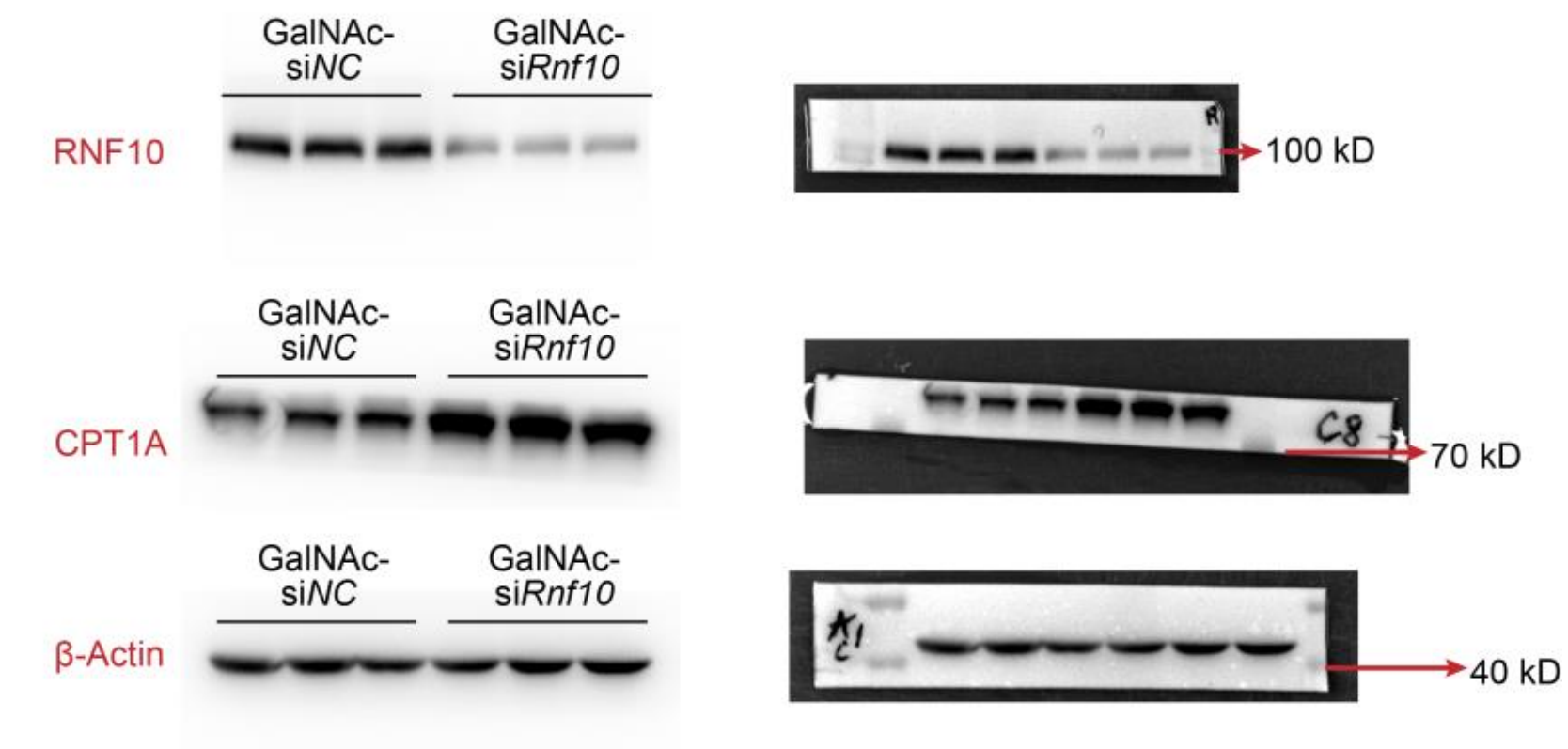

S

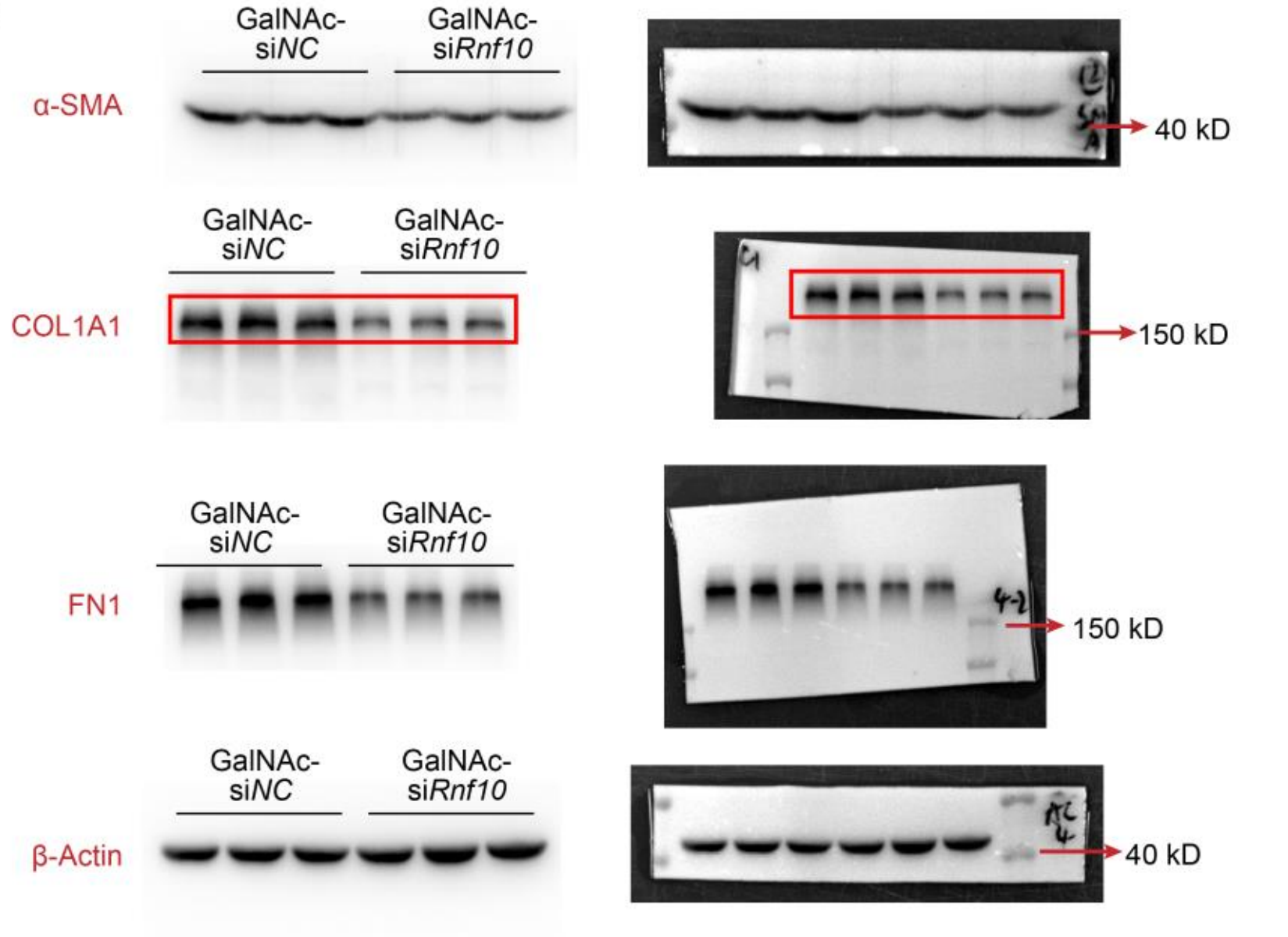

Full unedited gel for supplemental Figure S1

C

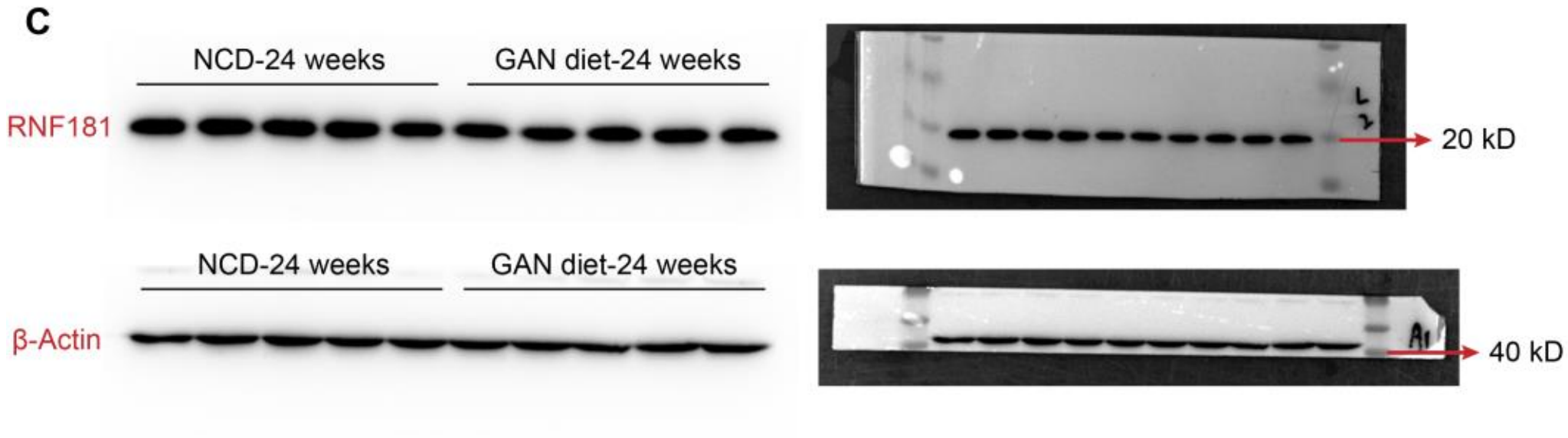

F

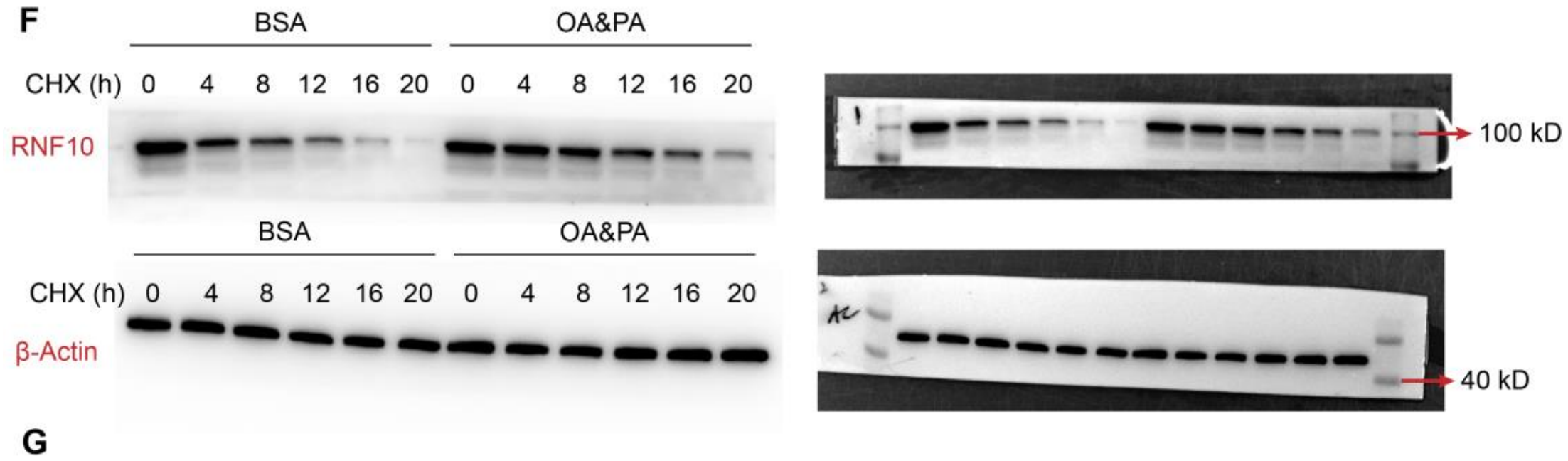

G

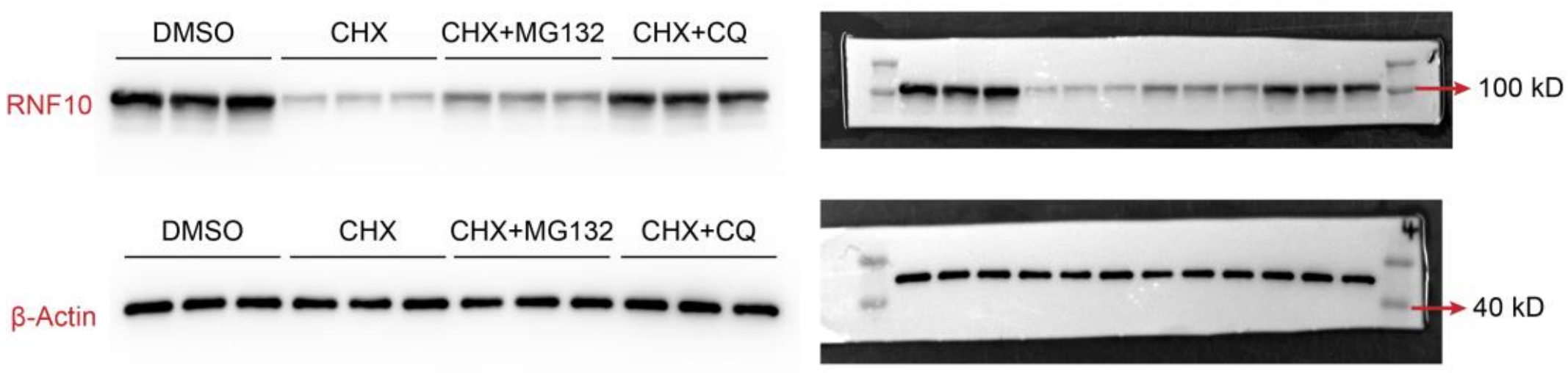

Full unedited gel for supplemental Figure 2

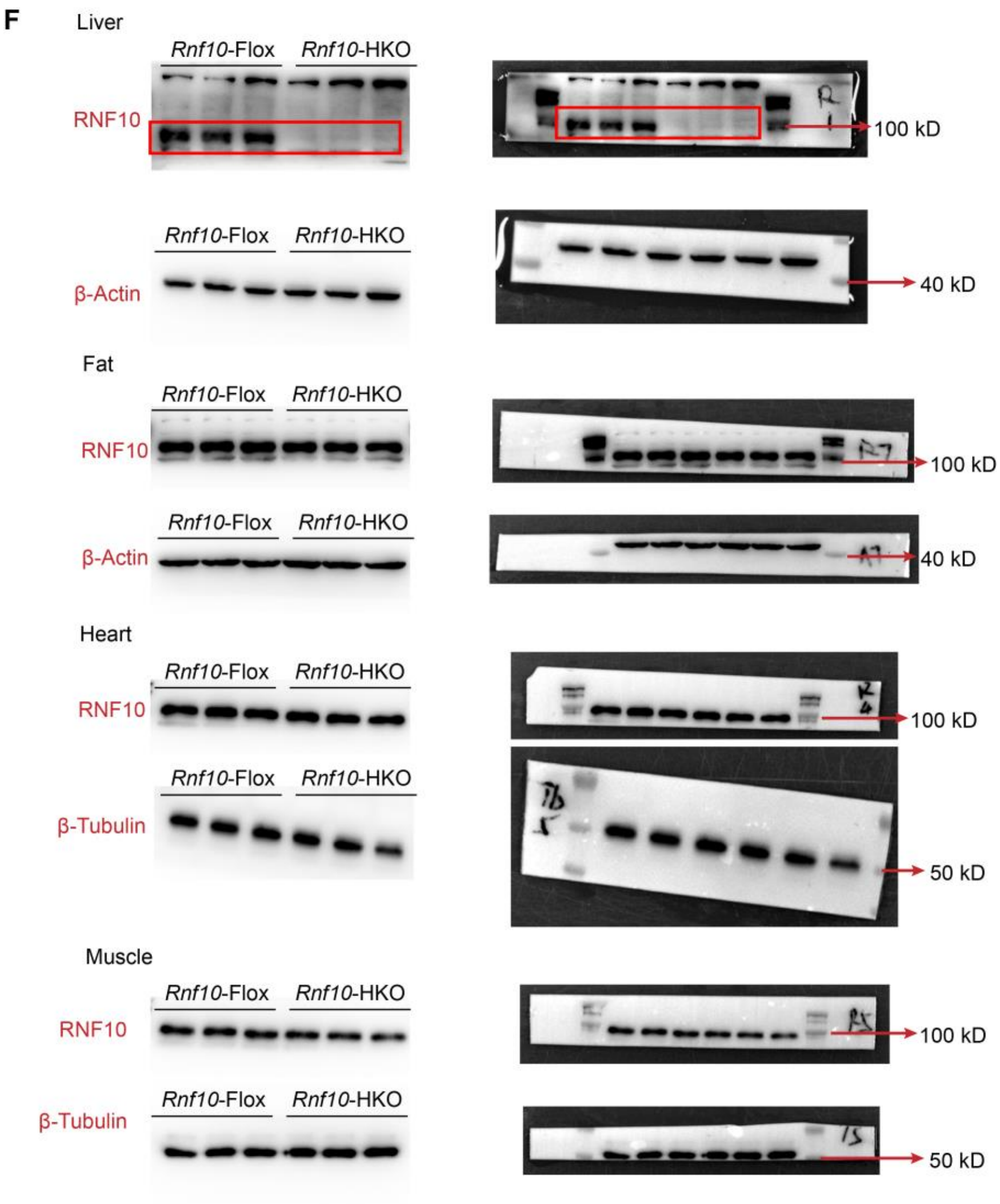

Full unedited gel for supplemental Figure 3

F

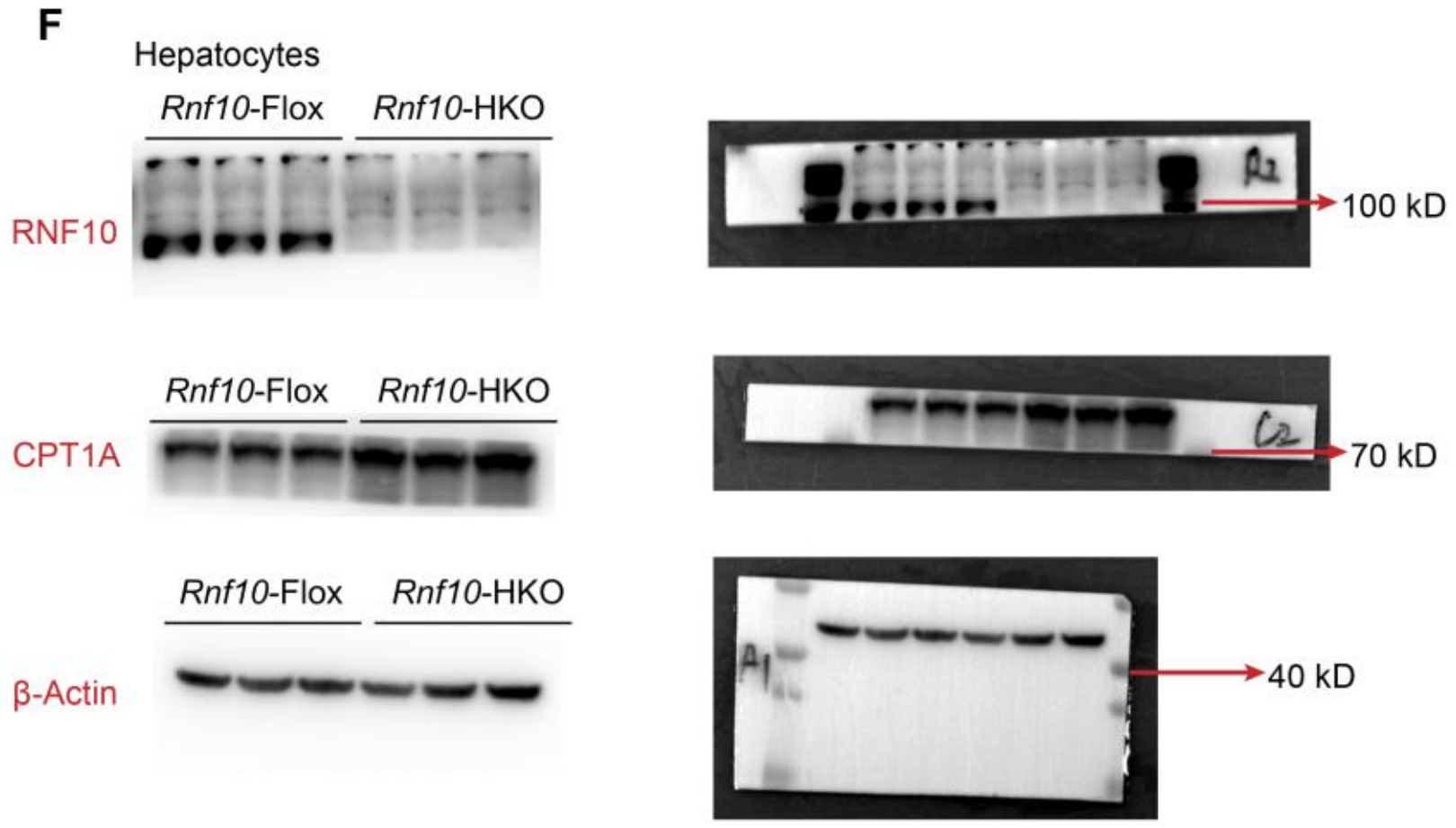

K

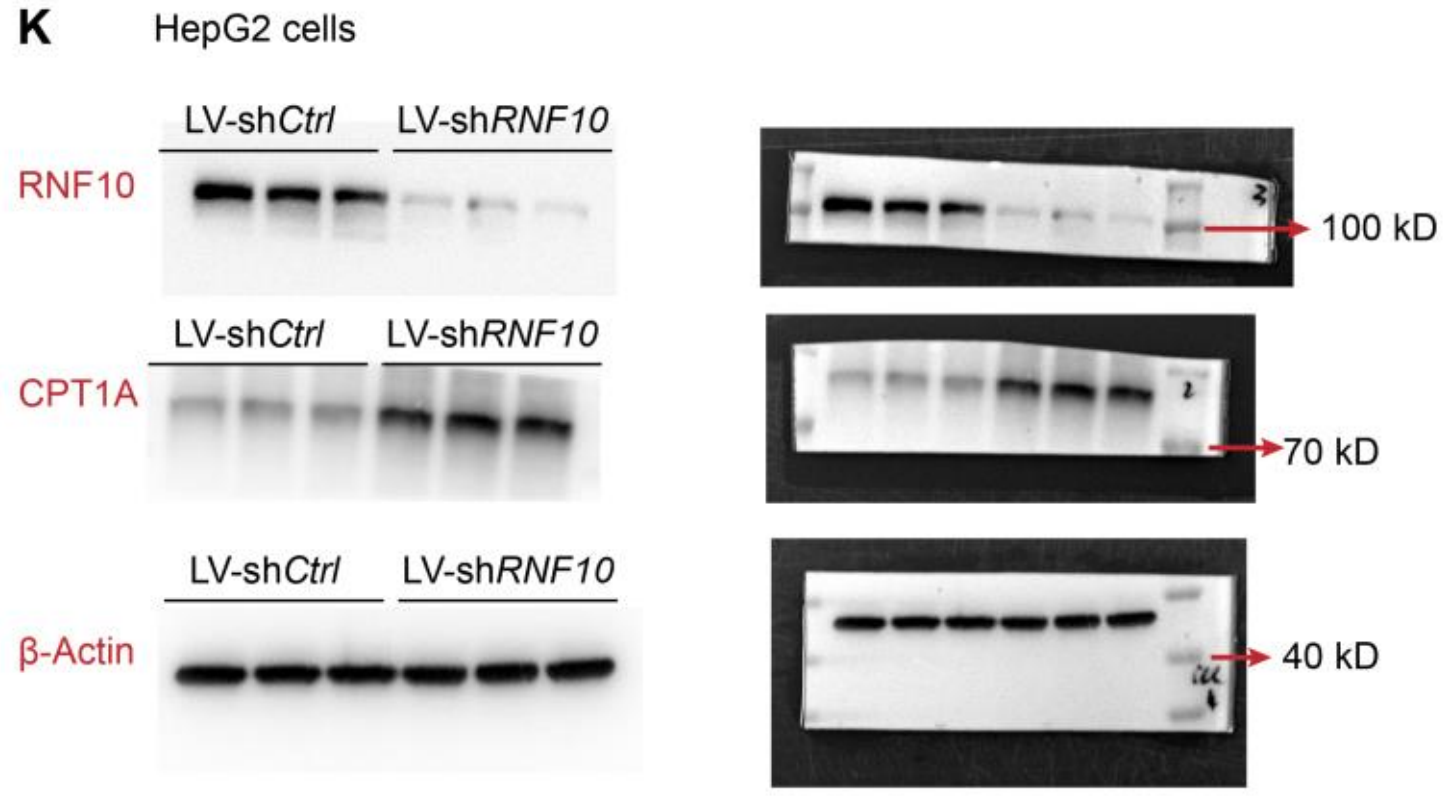

P

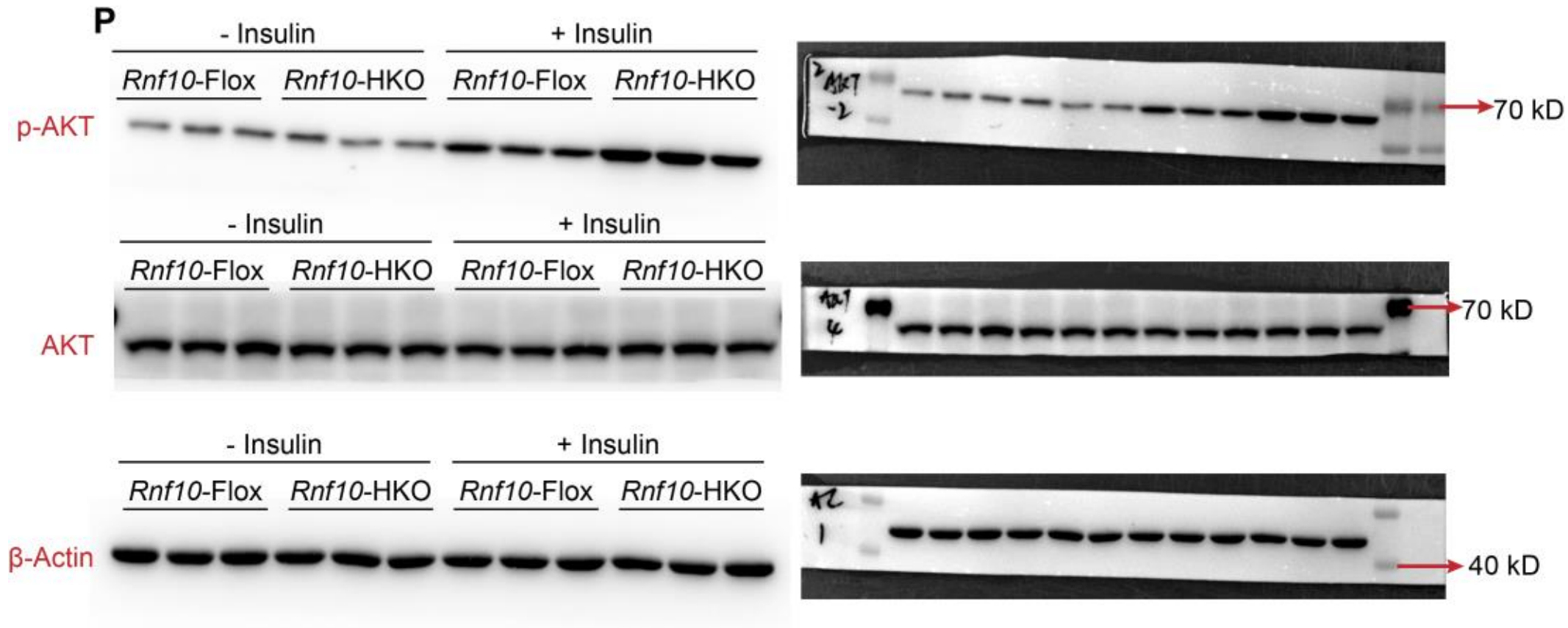

Full unedited gel for supplemental Figure 4

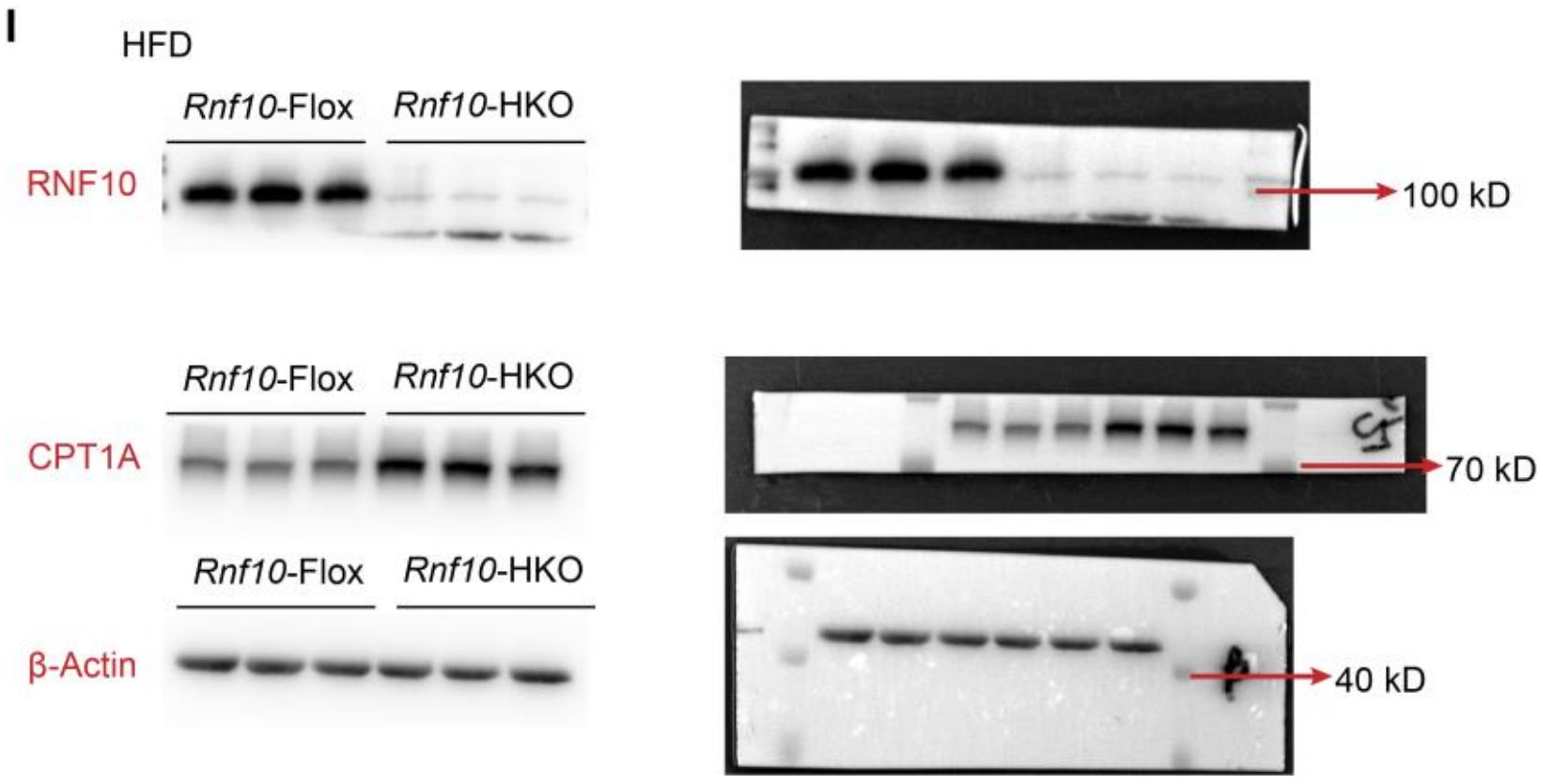

Full unedited gel for supplemental Figure 5

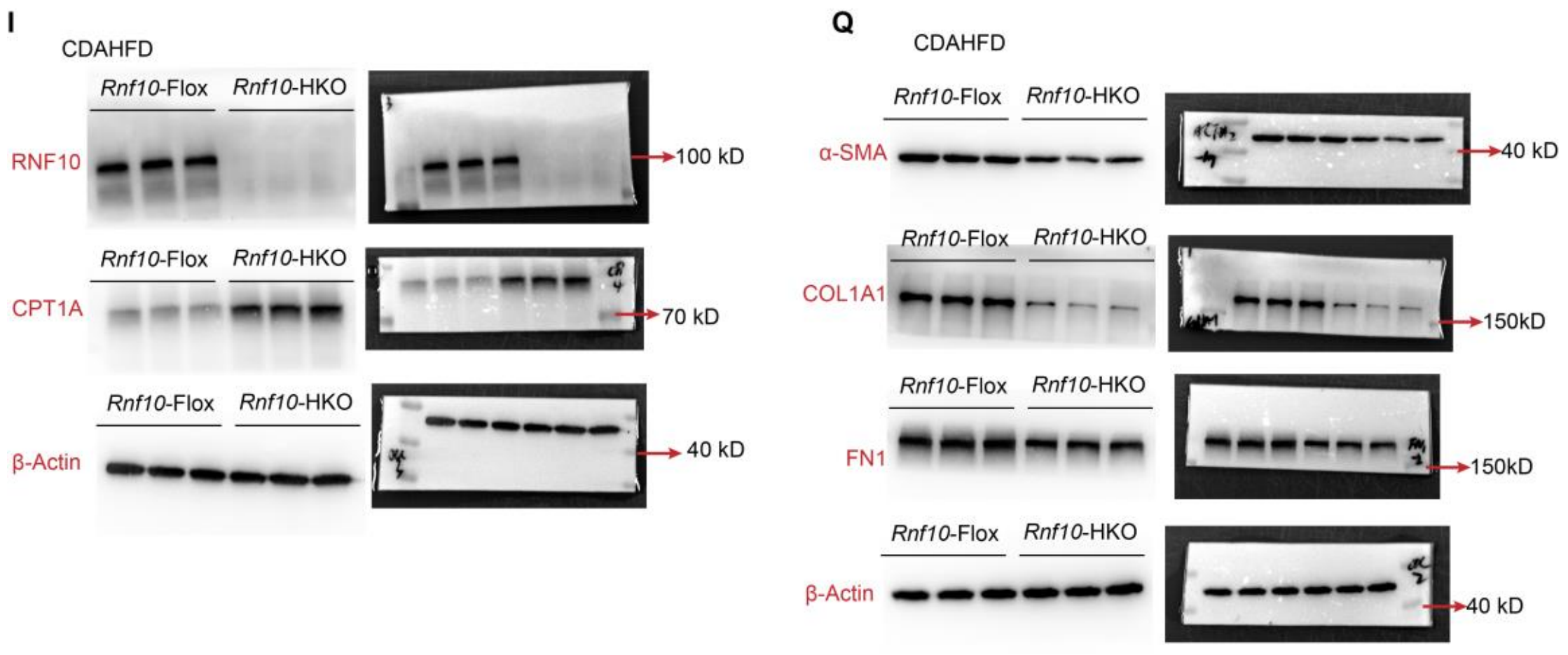

Full unedited gel for supplemental Figure 6

B

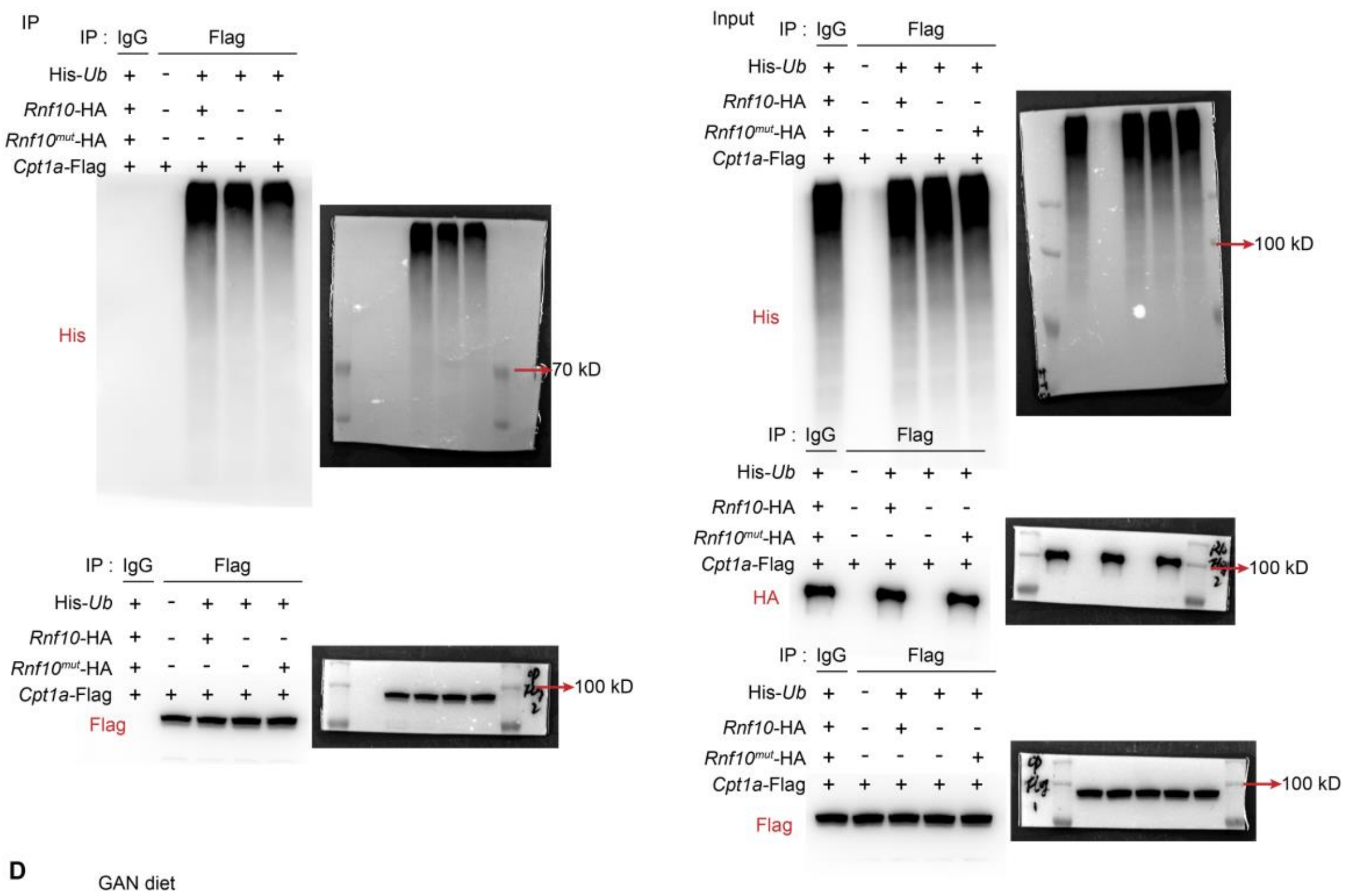

D

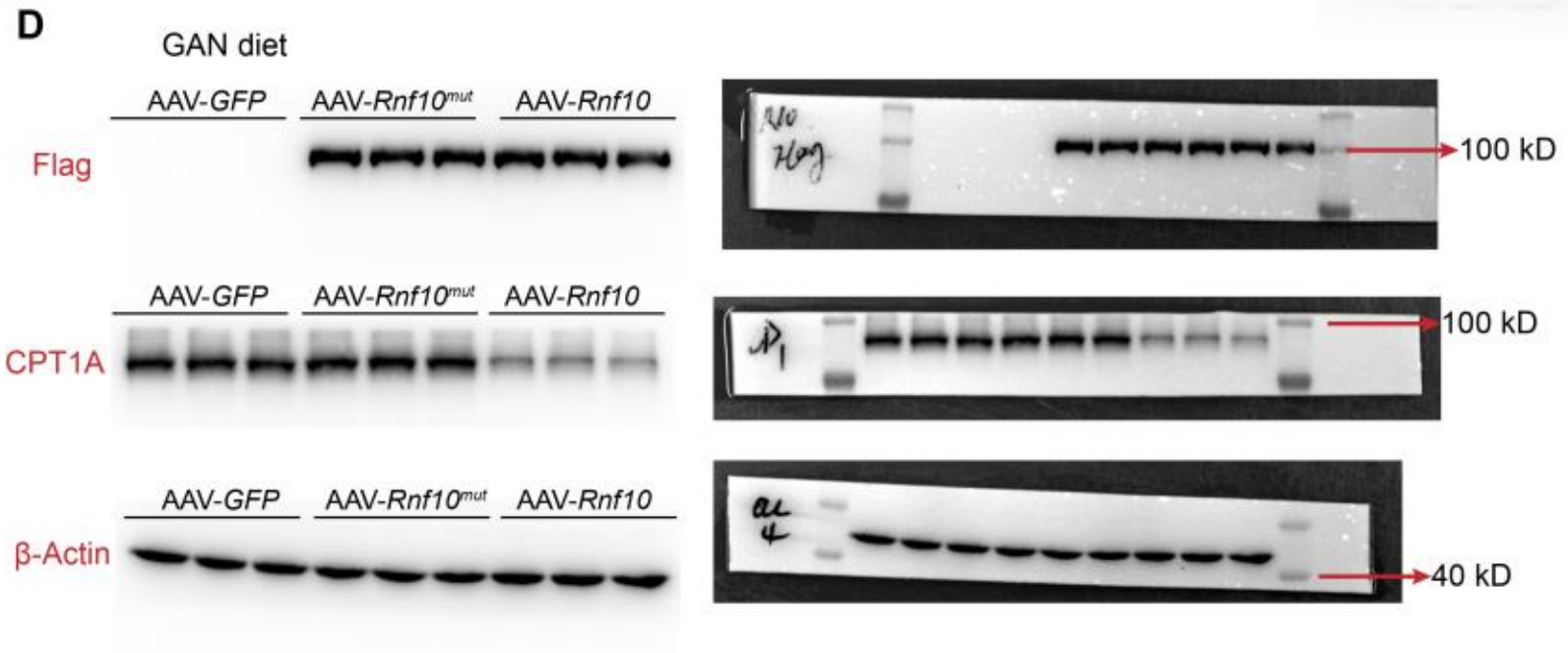

P

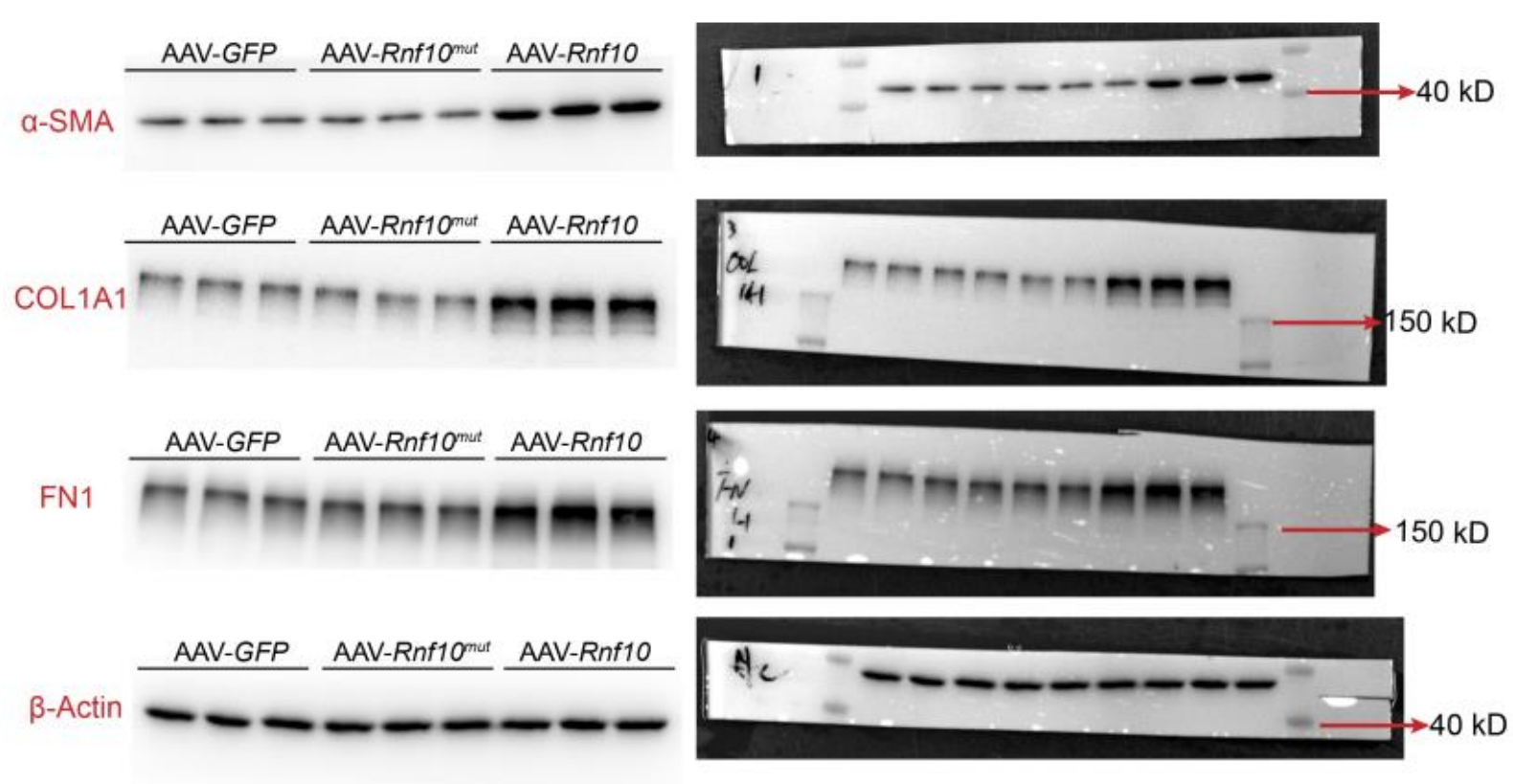

Full unedited gel for supplemental Figure 7

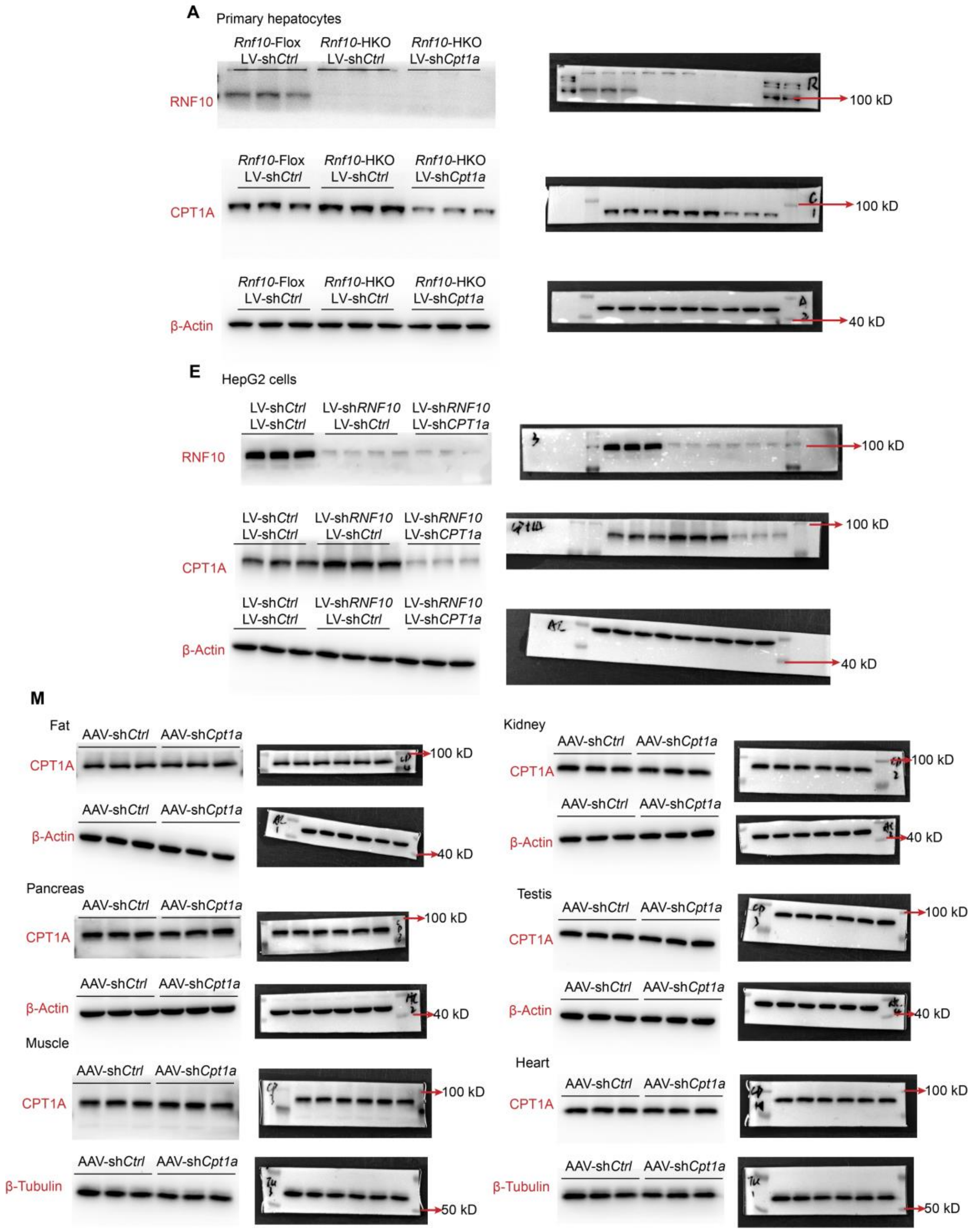

Full unedited gel for supplemental Figure 8

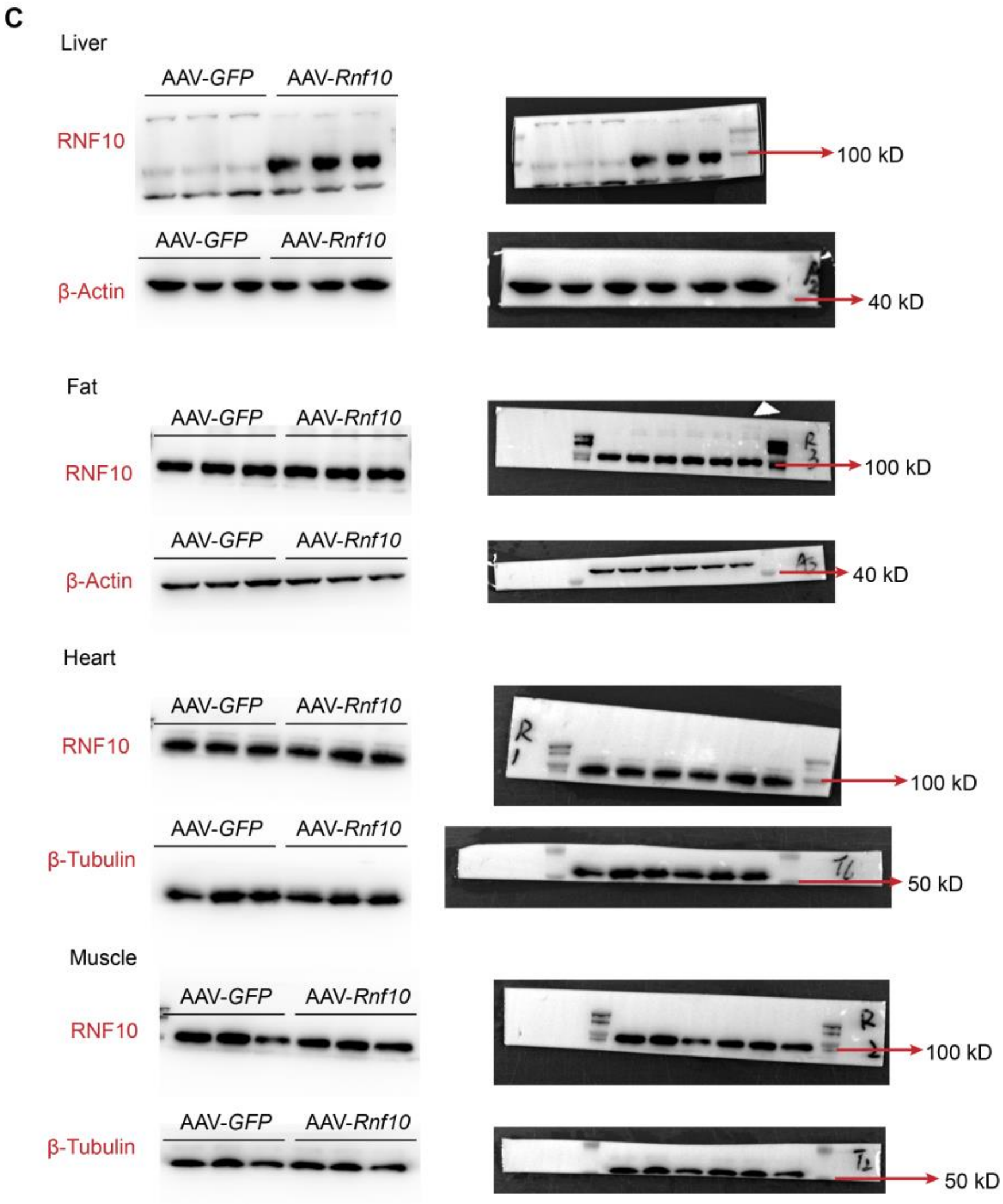

Full unedited gel for supplemental Figure 9

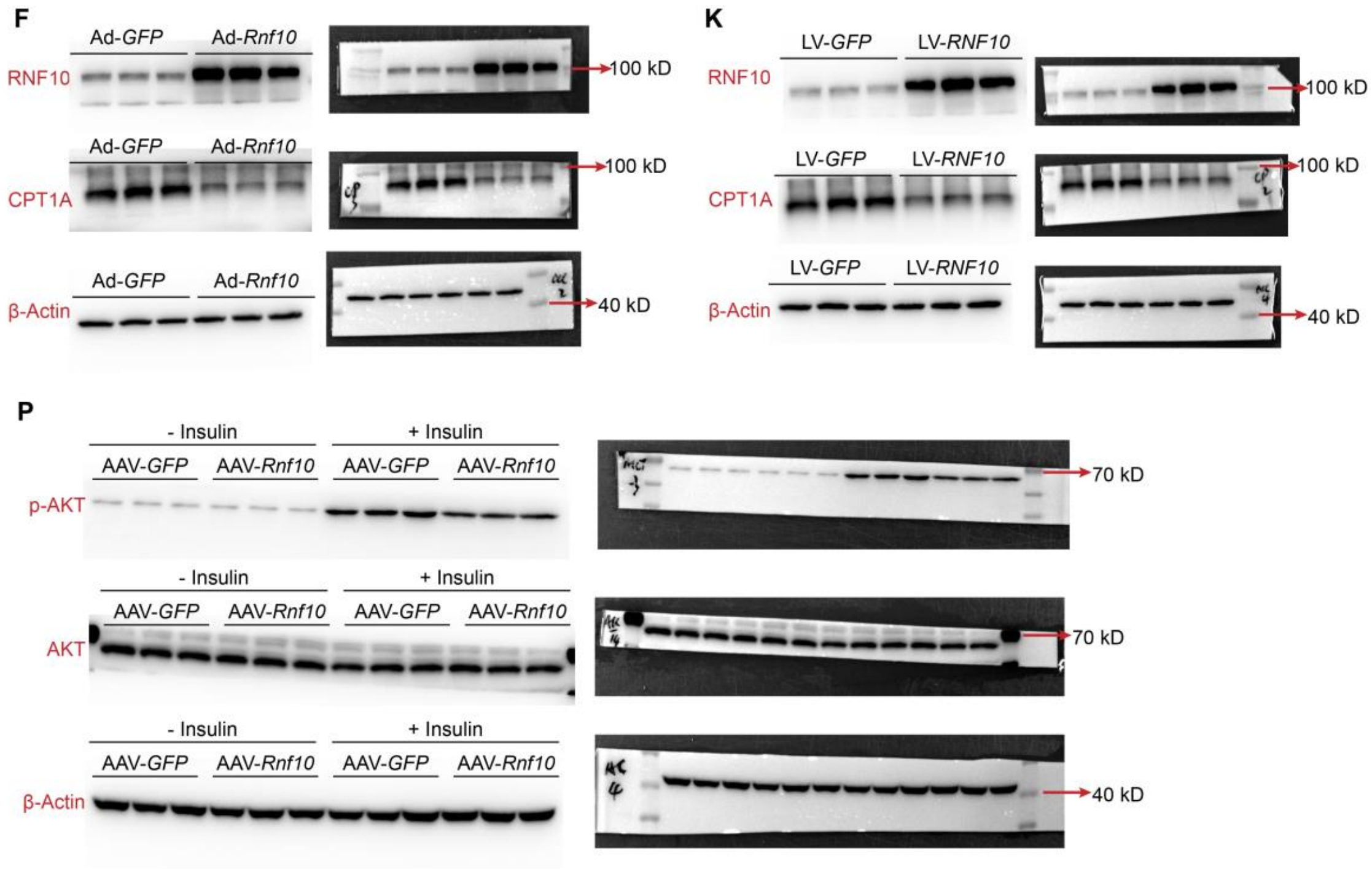

Full unedited gel for supplemental Figure 10

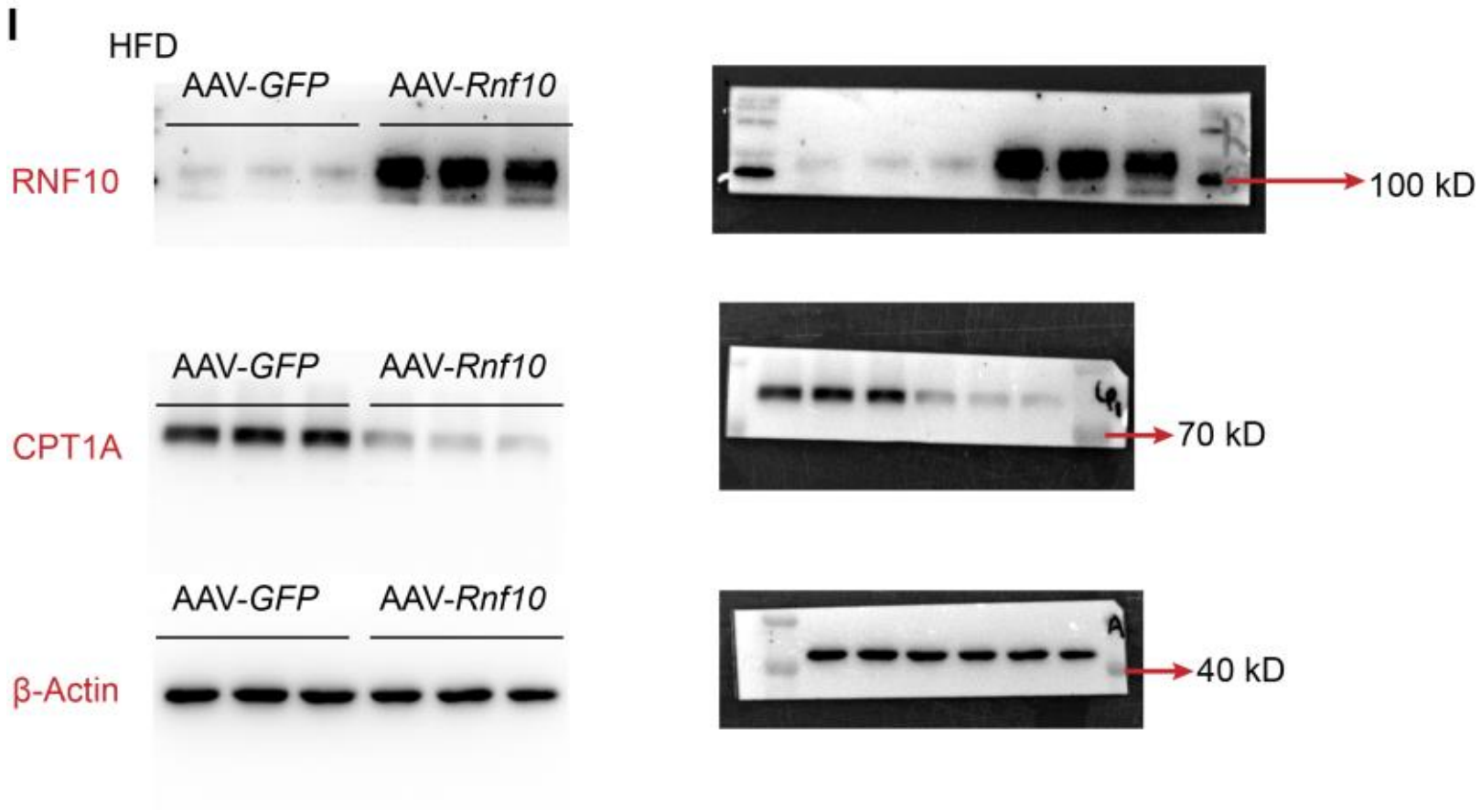

Full unedited gel for supplemental Figure 11

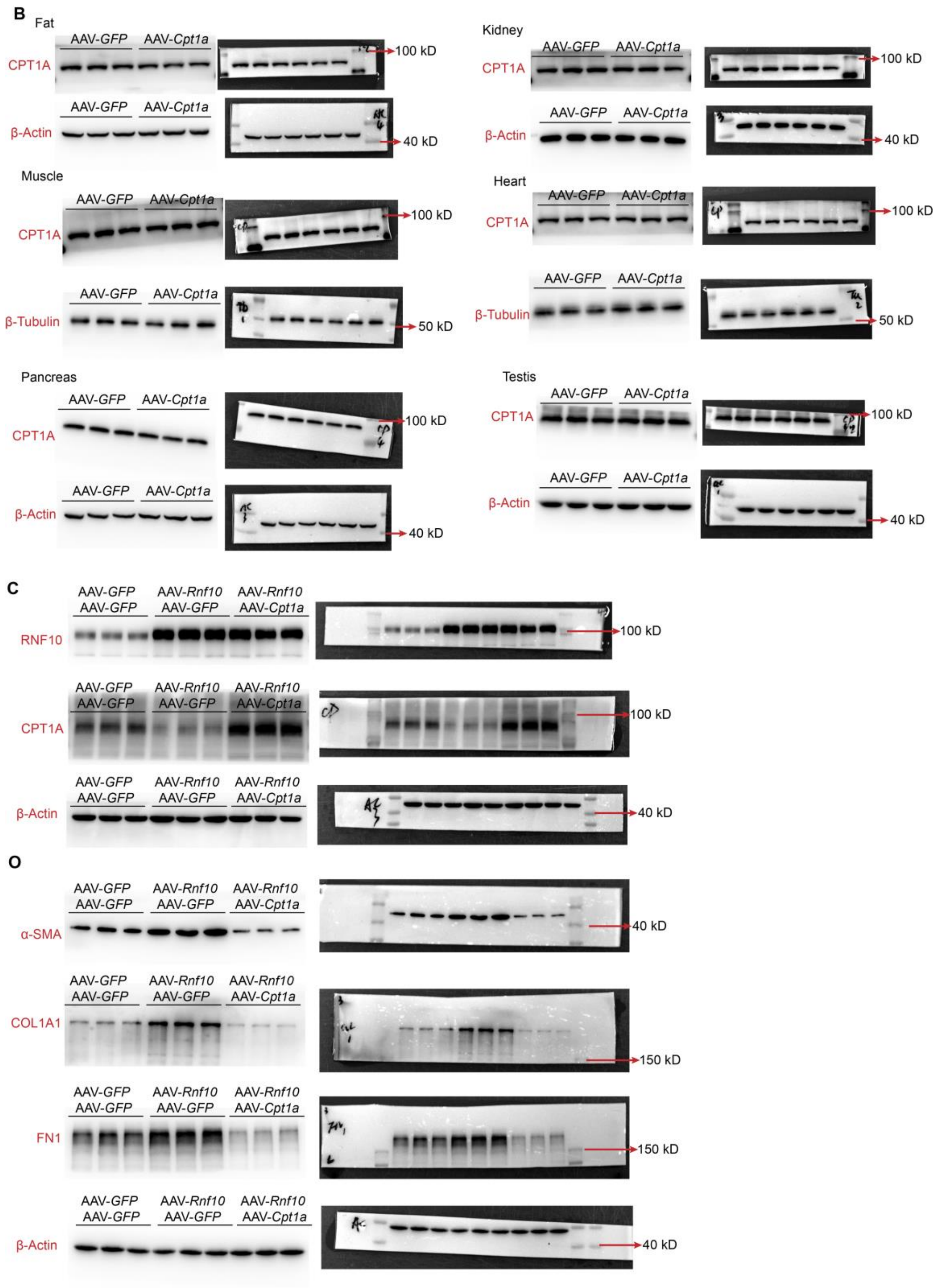

Full unedited gel for supplemental Figure 12

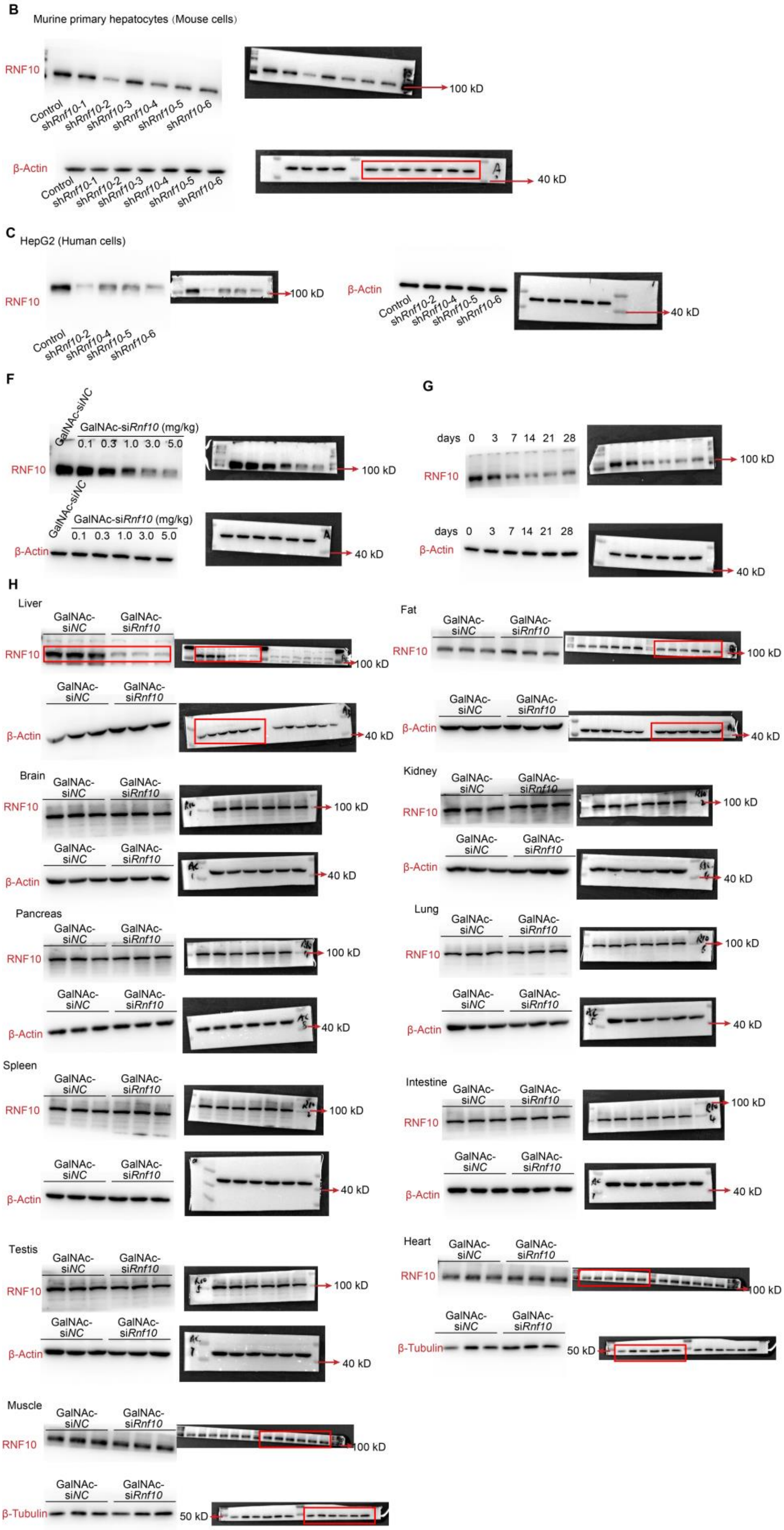

Full unedited gel for supplemental Figure 14

L

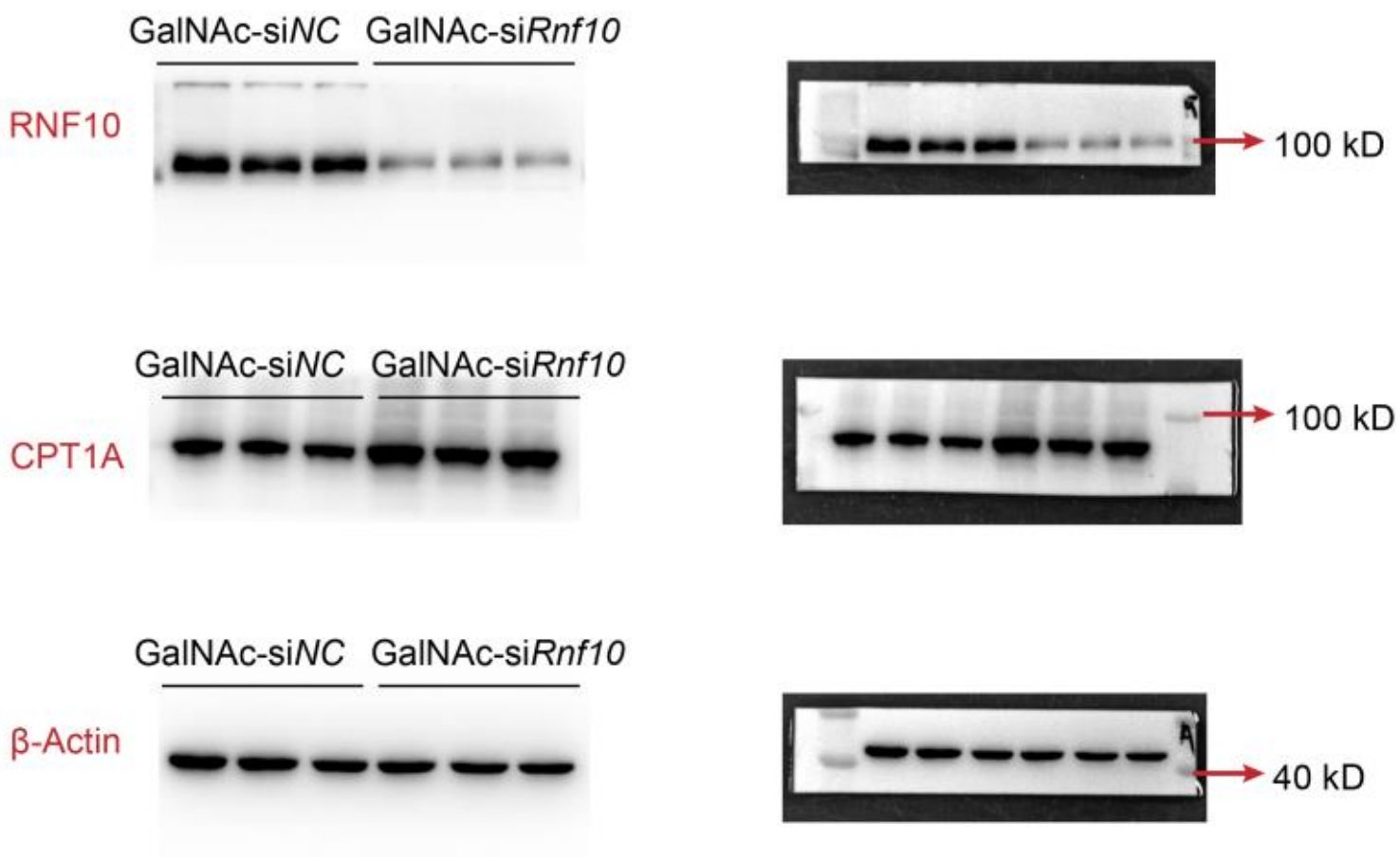

Supplement: Supplementary file 2 [file jci-136-194148-s112.pdf]
